# Neuronal Enriched Extracellular Vesicle miR-122-5p as a Potential Biomarker for Alzheimer’s Disease

**DOI:** 10.3390/cells14221784

**Published:** 2025-11-13

**Authors:** Kumudu Subasinghe, Courtney Hall, Megan Rowe, Zhengyang Zhou, Robert Barber, Nicole Phillips

**Affiliations:** 1 Department of Microbiology, Immunology and Genetics, UNT Health, 3500 Camp Bowie Blvd, Fort Worth, TX 76107, USA; kumudusubasinghe@my.unthsc.edu (K.S.); megan.rowe@unthsc.edu (M.R.); 2 Department of Biomedical Engineering, Johns Hopkins University, 3400 N Charles St, Baltimore, MD 21201, USA; chall106@jh.edu; 3 Department of Population and Community Health, College of Public Health, UNT Health, 3500 Camp Bowie Blvd, Fort Worth, TX 76107, USA; zhengyang.zhou@unthsc.edu; 4 Department of Family Medicine & Manipulative Medicine, Texas College of Osteopathic Medicine, UNT Health, 3500 Camp Bowie Blvd, Fort Worth, TX 76107, USA; robert.barber@unthsc.edu; 5 Institute for Translational Medicine, UNT Health, 3500 Camp Bowie Blvd, Fort Worth, TX 76107, USA

**Keywords:** Alzheimer’s disease (AD), Cognitive Impairment (CI), Extracellular Vesicles (EVs), Neuronal Enriched Extracellular Vesicles (NEEVs), MicroRNA (miRNA), Mexican Americans (MAs), Non-Hispanic Whites (NHWs)

## Abstract

**Highlights:**

**What is the implication of the main finding?**

- hsa-miR-122-5p was significantly underrepresented in cognitively impaired individuals in both Mexican American (MA) and Non-Hispanic White (NHW) groups.
- Population-specific NEEV miRNAs (MAs: hsa-miR-26a-5p, hsa-let-7f-5p, and hsa-miR-139-5p, NHWs: hsa-miR-133a-3p, hsa-miR-125b-5p, and hsa-miR-100-5p) were identified as potential blood-based biomarkers for Alzheimer’s disease (AD) and related cognitive impairment.

**What are the implications of the main findings?**

- Differentially expressed miRNAs were associated with APOE genotype, age, and metabolic burden, and their predicted targets are enriched in NF-κB-inflammatory pathways.
- Dysregulated miRNA networks may provide a mechanistic link between comorbidity burden and AD-related neuroinflammation, supporting their utility in AD precision medicine.

**Abstract:**

Alzheimer’s disease (AD) is the leading cause of dementia and is often prefaced by mild cognitive impairment (MCI). Detection of AD-related changes via blood-based biomarkers would enable critical therapeutic interventions early in disease progression. Neuronal enriched extracellular vesicle (NEEV) miRNAs regulate peripheral genes as a response to early AD brain changes and hence may have biomarker potential. Plasma NEEVs were captured from plasma samples of Mexican Americans (MAs) and Non-Hispanic Whites (NHWs) using an antibody against the neuronal surface marker CD171. miRNAs isolated from NEEVs were sequenced and analyzed using miRDeep2/DEseq2 and QIAGEN RNA-seq portal for differential expression between cognitively impaired (CI) and cognitively unimpaired controls. hsa-miR-122-5p was significantly underrepresented in the CI group in both MAs and NHWs compared to the healthy control. Other population-specific miRNAs (MAs: hsa-miR-26a-5p, hsa-let-7f-5p, and hsa-miR-139-5p, NHWs: hsa-miR-133a-3p, hsa-miR-125b-5p, and hsa-miR-100-5p) identified may have biomarker potential in AD precision medicine. Some of these differentially expressed miRNAs were associated with key AD-related comorbidities such as APOE genotype, age, and metabolic burden and were predicted to target genes within NF-κB -regulated inflammatory pathways. Together, these findings suggest that dysregulated miRNA networks may serve as a mechanistic link between comorbidity burden and AD-related neuroinflammation and neurodegeneration.

## 1. Introduction

Alzheimer’s disease (AD) is a progressive, age-related neurological illness and the leading cause of dementia in the older population [1]. Its development is influenced by genetic, epigenetic, and environmental factors, many of which disproportionately affect specific population groups [2]. The earliest stages of AD include two stages—preclinical AD and mild cognitive impairment (MCI)—which progress insidiously, with clinical symptoms often emerging decades after the onset of neuropathological changes [3,4]. Early detection of AD-related cognitive impairment is essential, as timely interventions may slow disease progression and improve quality of life for both patients and caregivers [5,6,7].

AD disproportionately impacts Mexican Americans (MAs) in the United States (US), who experience earlier onset, rapid age-related cognitive decline, and severe dementia compared to Non-Hispanic Whites (NHWs) [8,9]. This disparity could be attributed to numerous risk factors overrepresented in the MA community, including compromised social determinants of health and higher prevalence of metabolic comorbidities such as Type 2 Diabetes (T2D) [10,11]. As one of the fastest-growing aging demographic groups, MAs constitute a large portion of the US Hispanic population [12]. By the 2060s, this group is projected to face a dramatic increase in AD and related dementias, resulting in profound societal, economic, and healthcare burdens [13]. Despite this urgent need, relatively few studies have focused on MA-specific AD health disparities or biomarker discovery.

Currently, a definitive AD diagnosis requires postmortem confirmation through identification of amyloid plaques and neurofibrillary tangles in brain tissue, while a clinical diagnosis of “probable AD” in living patients relies on invasive and costly procedures such as CSF biomarker testing and MRI/PET imaging. Thus, accurate and accessible early-stage diagnostic tools are critical [14,15,16,17]. Blood-based biomarkers offer a minimally invasive, cost-effective alternative for early AD detection [3]. Among these, extracellular vesicles (EVs) have emerged as promising candidates due to their role in mediating AD-related pathological changes. EVs facilitate cell-to-cell communication by transporting nucleic acids, proteins, lipids, and metabolites [18,19]. Small extracellular vesicles (sEVs), ranging from 30 to 200 nm, can be subtypes using surface markers, including the neuronal marker CD171, to enrich for brain-derived vesicles [20,21,22,23,24]. Because CD171 (L1CAM) is not exclusively neuronal and may co-isolate EVs from other cell types, we refer to the isolated vesicles as neuronal-enriched extracellular vesicles (NEEVs) to more accurately reflect their origin [22].

**Figure 1:**
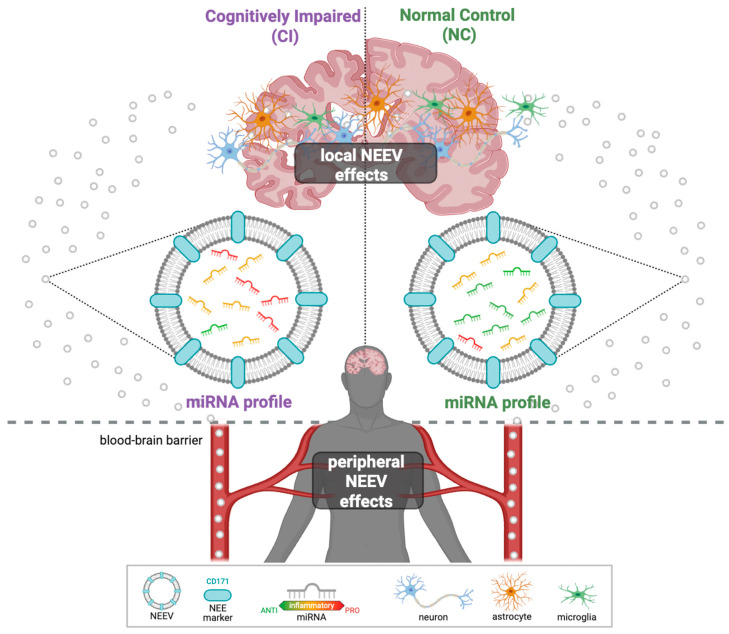
Graphical illustration of NEEVs released into the peripheral blood. NEEVs cross the blood barrier and can be isolated from the peripheral blood using the neuronal-enriched protein marker CD171. Their miRNA cargos can then be used to differentiate AD patients from healthy individuals.

The molecular cargo of NEEVs could provide unique insights into neurodegenerative processes (Figure 1). In particular, NEEV-derived microRNAs (miRNAs) can modulate the expression of inflammatory genes and other target gene networks by either inhibiting messenger RNA (mRNA) translation or promoting mRNA degradation [25]. Encapsulation within the vesicle lipid bilayer protects these miRNAs from degradation, preserving their bioactivity during transport within the brain and across the blood–brain barrier. NEEV miRNAs therefore have the potential to influence oxidative stress responses and other mechanisms central to neurodegeneration [20,26,27,28]. This makes NEEV-miRNA profiling a promising biomarker strategy in AD and related disorders [20].

Longitudinal sampling is critical in AD research, as changes in EV-miRNA cargo may better reflect disease trajectory than single time-point comparisons. Tracking miRNA dynamics over time offers the potential to identify biomarkers that signal cognitive decline progression before clinical impairment becomes more pronounced. Therefore, incorporating multiple follow-up visits is an important step toward establishing EV-miRNAs as temporal markers of AD pathology and disease progression.

**Figure 2:**
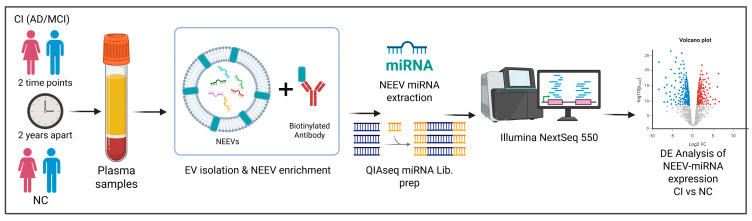
Workflow of the experimental design. Longitudinal plasma samples (two time points, 2 years apart) were processed using a two-step method that involves precipitation of total exosomes followed by NEEV capture with a biotinylated antibody against the neuronal surface marker CD171. RNA was isolated from NEEVs and profiled via next-generation sequencing and analyzed for differential miRNA expression in individuals with cognitive impairment (CI) compared to the normal control (NC) group.

In this study, we investigate the population-specific biomarker potential of NEEV-miRNAs in AD through three complementary approaches: (1) identifying differential expression (DE) patterns associated with cognitive impairment, (2) monitoring longitudinal expression changes with disease progression, and (3) assessing the impact of key covariates such as metabolic comorbidities (T2D, hyperlipidemia, hypertension), gender, APOE ε4 allele status, and age (Table 1). Our analyses use plasma-derived NEEVs from MA and NHW participants clinically diagnosed with AD or MCI but without imaging confirmation, collectively referred to here as cognitively impaired (CI). By integrating this unique sample type with our innovative workflow (Figure 2), we provide novel insights into the role of NEEV-miRNA cargo in AD pathogenesis and establish a conceptual framework for applying blood-based miRNA profiling to other complex diseases.

## 2. Materials and Methods

### 2.1. Participants

Frozen human plasma samples from Mexican American (MA) and non-Hispanic White (NHW) participants diagnosed with cognitive impairment (AD and MCI) as well as normal controls (NCs) were obtained from the Texas Alzheimer’s Research and Care Consortium (TARCC). For each participant, two plasma samples collected at independent visits two years apart (Visit 1 and Visit 2) were available. Each Visit 1–Visit 2 pair was processed together in a single batch across the entire workflow, from NEEV isolation through sequencing.

### 2.2. Total EV Isolation

Plasma samples were stored at −80 °C use. Prior to EV isolation, samples were thawed overnight on ice at 4 °C, inverted to mix, and incubated at room temperature (RT) for 10 min. Up to 1.2 mL of plasma was transferred to 1.5 mL Protein LoBind tubes and centrifuged (3000× *g*, 4 °C, 15 min, acceleration/deceleration: 9/5). The supernatant was collected and kept on ice.

Thrombin (611 U/mL, System Biosciences, Palo Alto, CA, USA, Cat. No. TMEXO-1) was added at 4 µL per 0.5 mL of plasma, mixed by inversion (3×), and rotated at low speed on a MACSmix Tube Rotor (Miltenyi Biotec B.V. & Co. KG, Bergisch Gladbach, Germany) until processing was complete. Samples were then incubated on a HulaMixer (Life Technologies AS, Oslo, Norway) (RT, 10 min, vibro: 5°/05) and centrifuged (1000 rpm, 4 °C, 5 min; acceleration/deceleration: 9/9). ExoQuick (System Biosciences, Palo Alto, CA, USA, Cat. No. EXOQ20A-1) was added according to the manufacturer’s instructions, mixed by inversion (10×), and incubated at 4 °C for 30 min. Samples were centrifuged (1500× *g*, 4 °C, 30 min; acceleration/deceleration: 9/9) to pellet total EVs. The supernatant was removed, and the EV pellet was resuspended in 1× PBS supplemented with 2× Halt Protease and Phosphatase Inhibitor Cocktail (Thermo Fisher Scientific, Waltham, Massachusetts, the United States, Cat. No. 78440) by incubating on a HulaMixer (Life Technologies AS, Oslo, Norway) (RT, vibro: 5°/05) with low-speed vortexing every 30 min [20,21,22,23,24].

### 2.3. NEEV Enrichment

Biotinylated anti-Human CD171 primary antibody (Thermo Fisher Scientific, Waltham, MA, USA, Cat. No. 13-1719-82) was diluted from 0.5 mg/mL stock to 0.1 mg/mL. Exo-Flow Streptavidin Magnetic Beads (System Biosciences, Palo Alto, CA, USA, Cat. No. CSFLOWBASICA-1) were washed twice with 1× Bead Wash Buffer (System Biosciences, Palo Alto, CA, USA, Cat. No. CSFLOWBASICA-1) and incubated with diluted CD171 for 3 h on ice, with gentle flicking every 30 min. After incubation, CD171-conjugated beads were washed three times, magnetically separated, and resuspended in 800 µL of Bead Wash Buffer.

**Figure 3:**
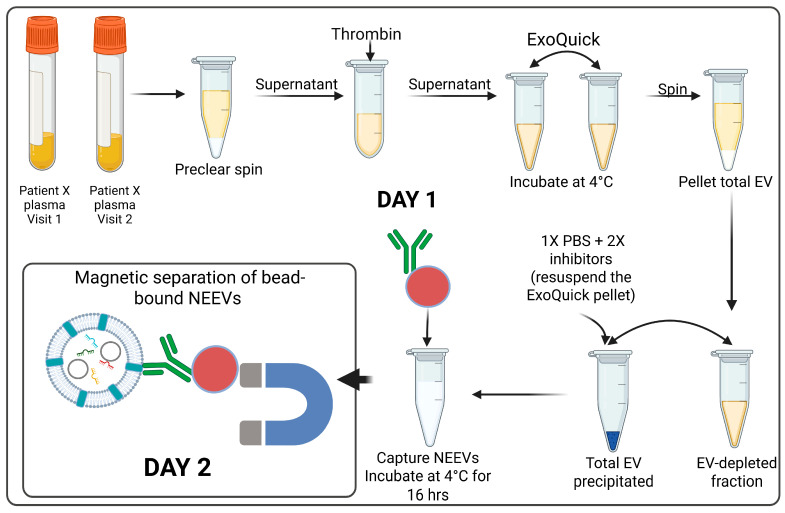
NEEV isolation from plasma samples. Day 1: Total EVs from plasma samples were precipitated using ExoQuick after clearing for impurities with preclear spin and the Thrombin treatment followed by starting the incubation of the EVs with biotinylated CD171 antibody conjugated to magnetic beads for NEEV capture overnight. Day 2: Bead-bound NEEVs were separated on a magnet.

Total EVs were added to the CD171-conjugated bead suspension and incubated for 16 h at 4 °C on a rotating rack. Bead-bound NEEVs were magnetically separated, washed twice with Bead Wash Buffer, and resuspended in 500 µL of 1× PBS. Isolated NEEVs were stored at −80 °C until RNA extraction (Figure 3).

### 2.4. NEEV Characterization

NEEVs were characterized according to the latest MISEV (Minimal Information for Studies of Extracellular Vesicles) guidelines [29]. Western blot was performed for seven protein markers (see Supplementary Document S1, Table S1): transmembrane protein CD9, cytosolic marker PDCD6IP/Alix, non-EV contaminant Calnexin, and three neuronal markers—transmembrane L1 cell adhesion molecule (L1CAM/CD171), neuronal cell adhesion molecule (NCAM), and ATPase Na+/K+ transporting subunit alpha 3 (ATP1A3) [24,30,31,32].

NEEV samples were lysed in 5× RIPA buffer and incubated on ice for 30 min. Protein concentration was determined by BCA assay (37 °C, 30 min incubation, absorbance OD562 nm). Proteins were denatured on metal heating block (3–5 min), separated by 12% SDS-PAGE gel, and transferred to polyvinylidene difluoride (PVDF) membranes. Primary antibodies were used at 1:1000 and secondary antibodies at 1:500.

### 2.5. RNA Extraction

Frozen NEEVs were thawed on ice before RNA extraction. Total RNA was extracted from bead-bound NEEVs using EVery EV RNA Isolation Kit (System Biosciences, Palo Alto, CA, USA, Cat. No. EVery100B-1) following the manufacturer’s protocol, including optional steps. Modifications included the following: (i) adding QIAseq miRNA library QC spike-in control (QIAGEN, Venlo, The Netherlands, Cat. No. 331535) at a 1:3 dilution during the lysis and (ii) elution incubation from 2 min to 5 min. RNA extracts were then concentrated to 5 μL using an Eppendorf 5301 Concentrator System (Eppendorf AG. 22331, Hamburg, Germany).

### 2.6. miRNA Library Preparation and Sequencing

Concentrated RNA was used to prepare libraries following the QIAseq miRNA UDI library protocol (QIAGEN, Venlo, The Netherlands, Cat. No. 331505) and QIAseq miRNA 96 Index Kit IL UDI-E (96) (QIAGEN, Venlo, Netherlands, Cat. No. 331935). Adapters and primers were diluted (3′ adapter: 1:10; 5′ UDI adapter: 1:5; RT primer: 1:10). Reverse-transcribed miRNAs were amplified with 22 PCR cycles on an Eppendorf Mastercycler (Eppendorf AG. 22331, Hamburg, Germany).

Library Quality control was performed using Agilent Tape Station 4200 (High-sensitive D1000 ScreenTape and reagents; Agilent, Santa Clara, CA, USA, Part Nos. 5067-5584, 5067-5585) (Supplementary Document S1, Figure S1). Final libraries were quantified with Qubit™ 1X dsDNA HS Assay (Thermo Fisher Scientific, Waltham, MA, the United States, Cat. No. Q33230) on a Qubit Flex Fluorometer (Thermo Fisher Scientific, Waltham, MA, USA, Cat. No. Q33327), diluted to 4 nM, and sequenced in 20-sample batches on the Illumina NextSeq 550 (72 bp single-end reads, 10 bp dual indexing, ~20 million reads/sample).

### 2.7. Data Analysis

#### 2.7.1. Differential miRNA Expression Analysis

Raw reads were analyzed using two different approaches. First, there was the QIAGEN RNA-seq Analysis portal (QIAGEN RNA-seq Analysis Portal 5.1, QIAGEN, Aarhus, Denmark, https://rnaportal.qiagen.com (accessed on 20 January 2025)) [33] with thresholds of False Discovery Rate (FDR) *p*-value ≤ 0.1 and fold change >1.1 or <−1.1. Second, there was a custom analysis pipeline: sequencing quality was assessed with FastQC v0.11.8 [34] and MultiQC v1.10.1 [35]; 3′ adapters were trimmed with cutadapt v1.15 [23]. Bowtie2 v2.3.5.1 [34] was used to generate index files from the human reference genome (GCF_000001405.40_GRCh38.p14, NCBI) and miRNAs quantification was performed with miRDeep2 v2.0.1.3 using hairpin and mature sequences from miRBase v22 [36,37].

Normalized counts were generated in R with DESeq2 v1.32.0 [38,39], filtering out miRNAs with mean count < 10. Differential expression (DE) analyses compared CI vs. NC groups within each population (MAs and NHWs) and across longitudinal points.

Volcano plots of DESeq2 analyses were created using VolcaNoseR v1.0.3, where the −log10 (*p*-values) are plotted against the log2FC values [40].

#### 2.7.2. Covariate Modeling

miRNA counts were modeled in DESeq2 with covariates including metabolic comorbidities, gender, presence of APOE ε4 allele, and age. A metabolic index (0–3) was defined as the sum of T2D, hyperlipidemia, and hypertension. An APOE4 index (0–2) reflected allele dosage. Both indices and age (60–80 years) were treated as continuous variables; gender (Female = 0, Male = 1) and diagnostic group (CI, NC) were treated as categorical variables. Batch effects were minimal by principal component analysis (PCA) in the QIAGEN RNA-seq portal (v5.1) and were excluded as a covariate (Supplementary Document S1; Figure S2).

For repeated measures, DREAM method [41,42] was applied, using linear mixed models to account for within-individual correlations, thereby improving power and reducing false positives in longitudinal RNA-seq datasets.

#### 2.7.3. Pathway Analysis and Target Genes

The biological relevance of miRNAs was explored using the microRNA Target Filter in Qiagen Ingenuity Pathway Analysis (QIAGEN Inc., https://digitalinsights.qiagen.com/IPA (accessed on 10 February 2025)) [43]. Gene network visualization of top disease-associated miRNAs was performed using miRNET v2.0 [43,44,45].

## 3. Results

### 3.1. EV Characterization

**Figure 4:**
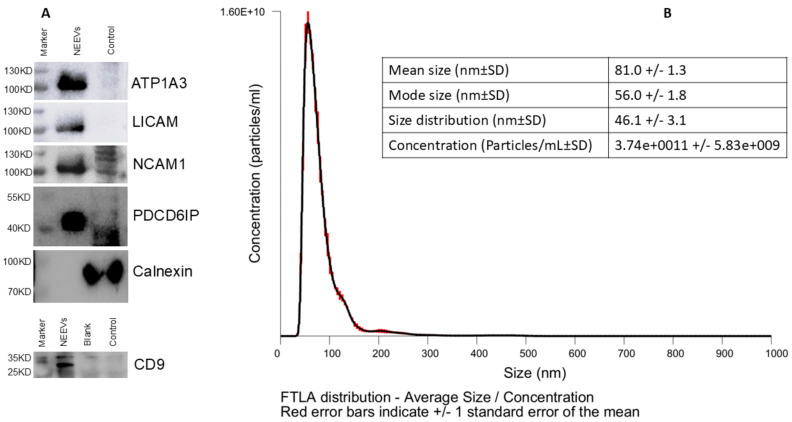
NEEV Characterization. (**A**) Biochemical characterization of NEEVs was performed using western blotting for CD09, Alix (PDCD6IP), and Calnexin, including three neuronal markers (LICAM, NCAM1 and ATP1A3). (**B**) NEEV size and yield was assessed via nanoparticle tracking analysis (NTA).

Western blotting confirmed the presence of EV markers CD9 and Alix and the absence of Calnexin, indicating successful NEEV enrichment with minimal cellular contaminations NEEVs were enriched in ATP1A3 along with L1CAM and NCAM, validating their neuronal origin. NTA revealed particles with a mean size of 81 nm and a mode of 56 nm, consistent with sEVs (Figure 4).

### 3.2. Differential Expression of miRNAs in NEEVs

#### 3.2.1. Quality Control

No clustering by batch was observed after excluding outliers, allowing batch to be excluded from the DESeq model (Supplementary Document S1; Figure S2).

#### 3.2.2. Representative miRNAs in NEEVs

Across all groups, hsa-miR-16-5p was highly expressed. Among the top 10 consistently expressed miRNAs were hsa-miR-122-5p, hsa-miR-423-5p, hsa-miR-126-3p, hsa-let-7i-5p, hsa-let-7b-5p, hsa-let-7a-5p, and hsa-let-7g-5p. miRNA hsa-miR-320a-3p was among the top 10 only in NC groups, while hsa-miR-191-5p was in the top 10 exclusively in the CI group (Supplementary Document S1, Figure S3).

#### 3.2.3. Differential Expression in Cognitive Impairment (CI)

**Figure 5:**
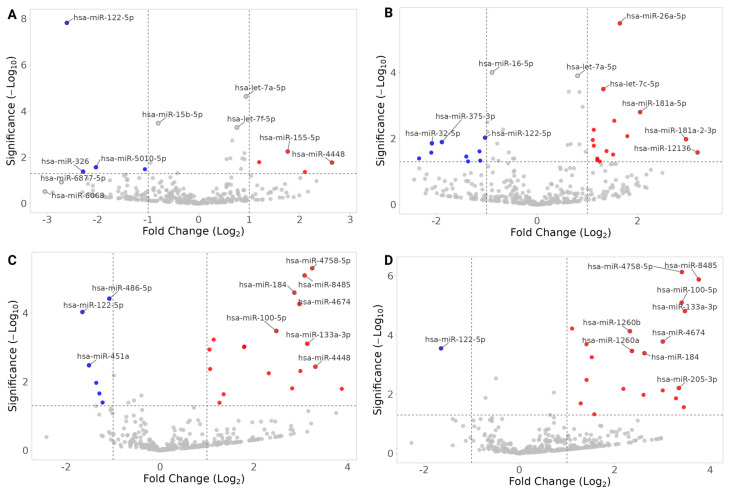
Volcano plots of DESeq2 miRNA expression analyses; CI vs. NC. (**A**) Mexican Americans, Visit 1, CI-vs-NC. (**B**) Mexican Americans, Visit 2, CI-vs-NC. (**C**) Non-Hispanic Whites, Visit 1, CI-vs-NC. (**D**) Non-Hispanic Whites, Visit 2, CI-vs-NC. Fold changes are with respect to CI (i.e., negative values are miRNAs underrepresented in CI-NEEVs when compared to NC). The top 10 hits are labeled in each plot, and miRNAs that have |FC| > 1 are filled with red (overrepresented in CI)/blue (underrepresented in CI). The horizontal dotted line indicates raw *p*-value of 0.05.

**Figure 6:**
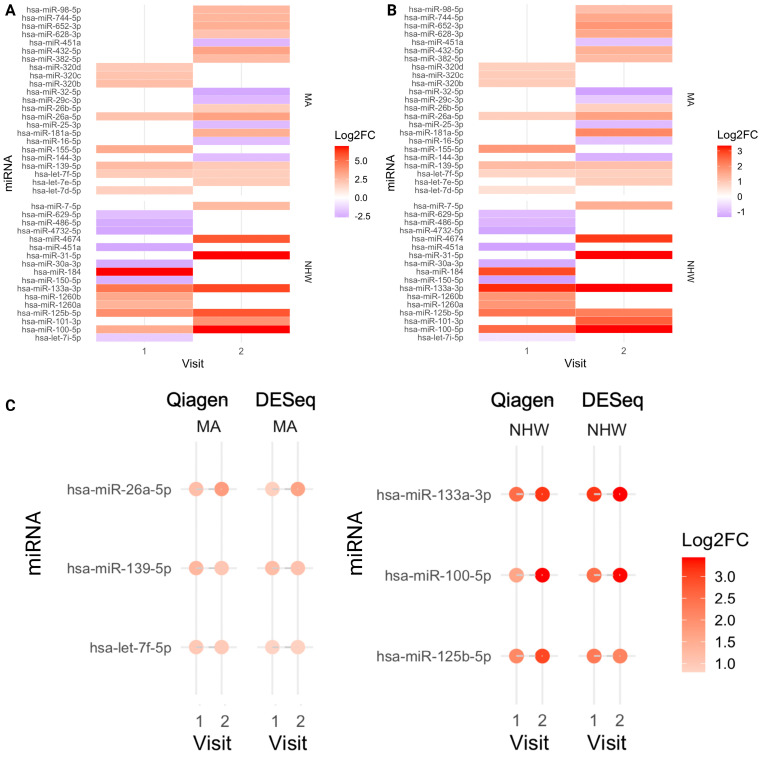
Heatmaps of miRNA expression; CI vs. NC, miRNAs unique to each population group. (**A**) QIAGEN platform; Mexican Americans and Non-Hispanic Whites, Visit 1 and 2, CI-vs-NC. (**B**) DESeq; Mexican Americans and Non-Hispanic Whites, Visit 1 and 2, CI-vs-NC. (**C**) Dot plot of population-specific miRNAs representing each visit expression. hsa-miR-26a-5p, hsa-let-7f-5p, and hsa-miR-139-5p were overrepresented in CI in Mexican Americans; hsa-miR-133a-3p, hsa-miR-125b-5p, and hsa-miR-100-5p were overrepresented in CI in Non-Hispanic Whites, in both visits.

Figure 5 and Figure 6 and Table 2, Table 3 and Table 4 summarize differentially expressed (DE) miRNAs in the CI group compared to NC, identified by both the QIAGEN and DESeq analyses.

##### Cross-Population Patterns

- hsa-miR-122-5p was consistently underrepresented in CI across MAs and NHWs (Table 2), a pattern that remained significant after adjusting for covariates (Figure 5, Table 2, Supplementary Document S2; Tables S1–S6). Males showed higher expression of hsa-miR-122-5p than females across populations, while no significant age association was observed in NHW (Supplementary Documents S3 and S4).
- hsa-let-7a-5p, hsa-let-7g-5p, and hsa-miR-122-5p were significantly DE in both MAs and NHWs when both the visits were analyzed (Supplementary Document S2; Table S6). hsa-miR-122-5p was underrepresented, and hsa-let-7a-5p was overrepresented in both population groups. hsa-let-7a-5p showed significant association with APOE4 index and the metabolic index in NHWs at Visit 2 but not in MAs. Notably, hsa-let-7g-5p was overrepresented in MAs but underrepresented in NHWs (Supplementary Document S1; Tables S2 and S3).
- miRNet linked these miRNAs to AD-, dementia-, and T2D-related genes (Supplementary Document S1; Figure S4).

Common DE miRNAs across both populations (identified by QIAGEN, DESeq, and DREAM) are listed in Supplementary Document S2; Tables S5–S7.

##### Population-Specific Findings:

- MAs: hsa-miR-26a-5p, hsa-let-7f-5p, and hsa-miR-139-5p were consistently overrepresented in CI and linked to AD-, hypertension-, and diabetes-related genes (Figure 6, Table 3; Supplementary Document S2, Tables S8 and S10; Supplementary Document S1; Figure S5). Several showed associations with APOE4, metabolic index, age, and gender (Supplementary Document S3).
- NHWs: hsa-miR-133a-3p, hsa-miR-125b-5p and hsa-miR-100-5p were overrepresented in CI and linked to hypertension-related genes (Figure 6, Table 4; Supplementary Document S2, Tables S9 and S11; Supplementary Document S1; Figure S6). Many NHW-specific miRNAs were strongly associated with metabolic index, APOE4, and demographic variables (e.g., hsa-miR-133a-3p increased with APOE4 index and metabolic index; hsa-miR-30c-1-3p higher in males; hsa-miR-1268a increased with age) (Supplementary Document S4).

#### 3.2.4. Longitudinal Changes

We examined longitudinal EV-miRNA expression between Visit 1 and Visit 2 (approximately two years apart). While a small number of miRNAs were identified as differentially expressed at Visit 2 relative to Visit 1 (Table 5), these findings were not consistent between the QIAGEN and DESeq2 analysis pipelines. Full longitudinal analysis results are provided in (Supplementary Document S2 Tables S12–S16).

## 4. Discussion

Here, we report that hsa-miR-122-5p is underrepresented in the CI group across both populations and shows a negative correlation with age in MAs. After adjusting for covariates, expression levels of hsa-miR-122-5p remained significantly reduced in both MAs and NHWs with CI, suggesting that this miRNA may exert a protective effect against cognitive decline. These findings indicate that hsa-miR-122-5p could serve as a viable blood-based biomarker of early cognitive impairment across populations and highlight the need for further investigation of its suppression in NEEVs.

Our study identified significant downregulation of hsa-miR-122-5p in NEEVs from CI subjects. This is consistent with Boccardi et al. (2023), who also reported reduced miR-122 in NEEVs from AD patients and linked it to inflammation, T2D, and atherosclerosis through their miRNet analysis [46]. Although hsa-miR-122-5p has been studied in various diseases, relatively few reports focus on its expression in EVs [47,48,49]. For example, Zhu et al. (2017) found that hsa-miR-122-5p was underrepresented in the peripheral blood of T2D patients [50], while Hashemi et al. (2023) [51] showed that hsa-miR-122-5p targets multiple hub genes implicated in AD pathology. Given its well-established role in regulating cholesterol and fatty acid metabolism in the adult liver, miR-122 may represent a promising therapeutic target for modulating AD pathogenesis through metabolic pathways [46,50,52].

We also observed high expression of several let-7 family candidates (hsa-let-7i-5p, hsa-let-7b-5p, hsa-let-7a-5p, and hsa-let-7g-5p; Supplementary Document S1; Figure S3). Notably, hsa-let-7g-5p was overrepresented in MAs with CI but underrepresented in NHWs with CI. Let-7a-5p, a member of this family, targets EGFR, a gene with a recognized role in AD and neurodegeneration [53,54,55]. Prior human studies have implicated plasma hsa-let-7a-5p in regulating innate immune genes such as TLR3, RIG-I, and MDA5 in AD patients with allergic conjunctival disease (ACD) [56], and in autoimmune disease (vitiligo) through modulation of autophagy and apoptosis [57]. While such findings underscore the biological relevance of this miRNA family to AD and immunomodulation, their biomarker potential remains inconclusive due to contradictory reports. For example, Kafshdooz et al. (2023) identified hsa-let-7g-5p as a potential biomarker for AD, reporting decreased levels in serum in patients with AD [58], whereas Elham et al. (2022) [59] and Durur et al. (2022) [23] reported increased serum or NEEV expression of hsa-let-7g-5p and related miRNAs in AD. Satoh et al. (2015) also identified 27 differentially expressed plasma miRNAs in AD patients [60] including let-7a-5p, let-7g-5p (common in CI across both populations); let-7e-5p, let-7f-5p, and miR-98–5p (overrepresented in CI of MAs); and miR-15a-5p (underrepresented in CI of MAs) (Supplementary Document S2; Table S8). Together, the literature and our findings suggest an important but complex role for the let-7 miRNA family in AD and AD-like cognitive impairment, potentially shaped by population-specific effects. These factors may underlie the inconsistent results across studies, highlighting the need for further work on their biomarker potential.

The associations we observed between specific miRNAs and comorbid conditions also highlight the interplay between comorbidities and AD progression. For example, hsa-miR-26a-5p, hsa-let-7f-5p, and hsa-miR-139-5p (unique to MA-CI cohort) are strongly associated with genes linked to AD, T2D, and hypertension, suggesting they may act as molecular mediators of comorbidity-related effects on AD onset or severity. Similarly, hsa-miR-133a-3p, hsa-miR-125b-5p, and hsa-miR-100-5p (specific to the NHW-CI cohort) are linked to genes involved in hypertension, underscoring the contribution of vascular dysfunction to AD risk.

**Figure 7:**
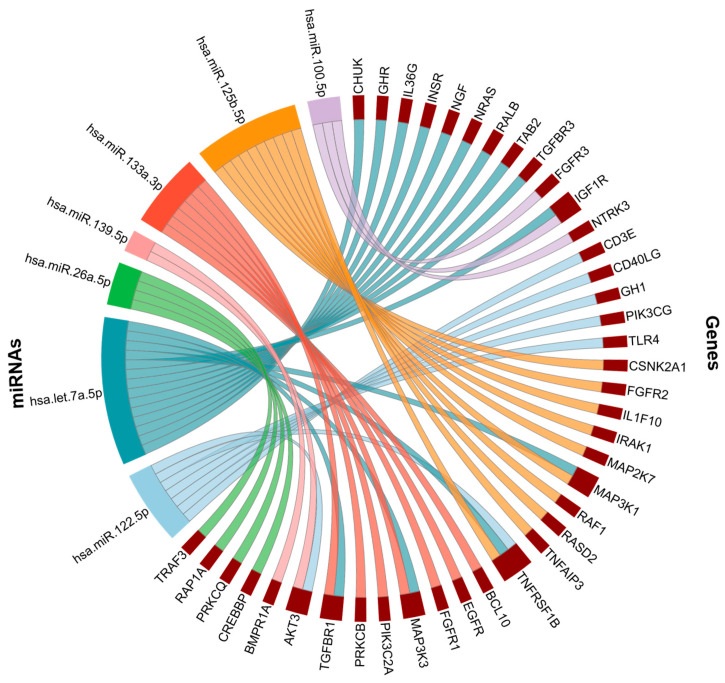
NF-kB signaling pathway associated genes. NF-kB genes targeted by hsa-miR-122-5p, hsa-let-7a-5p, hsa-miR-26a-5p, hsa-miR-139-5p, hsa-miR-133a-3p, hsa-miR-125b-5p, hsa-miR-100-5p.

Many of these miRNAs converge on the NF-κB signaling pathway (Figure 7), a central regulator of inflammation, immune responses, and cellular stress, which is widely implicated in both AD and comorbid conditions such as diabetes and hypertension. This overlap suggests that miRNA-mediated dysregulation of NF-κB signaling may represent a shared mechanism linking comorbidities with AD-related neuroinflammation and neurodegeneration.

Increasingly, many DE miRNAs unique to the NHW-CI cohort were associated with known AD risk factors such as APOE e4 allele and metabolic comorbidities, whereas relatively few MA-specific miRNAs showed similar association. This divergence underscores the potential value of population-specific miRNA profiling in guiding more precise diagnostic and therapeutic approaches to AD. The observed differences between Mexican Americans and non-Hispanic White participants underscore the importance of considering ancestry-specific biology in AD research. The distinct NEEV-miRNA profiles and their associations with metabolic burden and APOE genotype suggest that population-specific genetic and health risk factors may shape molecular signatures of cognitive impairment. Further work in larger, diverse cohorts will be needed to clarify how these ancestry-linked miRNA differences contribute to diagnostic performance and AD health disparities.

Although our pathway analyses highlight the potential biological functions of the differentially expressed miRNAs, these observations remain correlative. It is therefore possible that NEEV-miRNA alterations reflect downstream consequences of neurodegeneration rather than drivers of AD pathology. Distinguishing cause from consequence will require mechanistic validation, including experimental modulation of key miRNAs such as miR-122-5p in relevant neuronal and glial models to assess their effects on AD-related pathways.

Despite the longitudinal design, we did not observe strong or concordant NEEV-miRNA changes over the two-year interval, and cognitive measures show minimal decline during this timeframe. This suggests that the sampling interval may have been too short and/or that more advanced pathology is required to detect temporal miRNA shifts.

### 4.1. Strengths

Our study followed MISEV 2023 guidelines for EV characterization, thereby ensuring proper terminology and methodological rigor. NEEVs were captured from plasma using the neuronal surface marker (L1CAM/CD171), with validation of neuronal enrichment through NCAM and ATP1A3 [24,30,31,32]. Both proteins have strong brain specificity, and their enrichment confirmed the neuronal origin of our isolated EVs.

We optimized protocols to maximize recovery from small plasma volumes (~1 mL). Every EV RNA kit yielded the highest miRNA mapped reads, accurate RNA spike-in representation, and minimal rRNA contamination compared to other alternative methods. For library preparation, we used the QIAGEN miRNA UDI protocol with dual-end adapter ligation, improving mature miRNA capture and minimizing non-specific RNA. Library quality was validated with Agilent TapeStation (Supplementary Document S1, Figure S1).

A key strength of this study is the use of two independent pipelines—QIAGEN RNA-seq Analysis Portal (with built-in parameters based on published work) [33] and miRDeep2/DESeq—both of which identified a shared set of significantly altered miRNAs across groups. These candidates (Table 2, Table 3 and Table 4) remained robust after covariate adjustment, highlighting their potential as reliable biomarker candidates. To further reduce false positives, we employed DREAM for longitudinal analysis, leveraging repeated measures to enhance statistical power [42]. Importantly, several identified candidate miRNAs (hsa-miR-122-5p, miR-15b-3p, let-7a-5p) have also been reported in prior EV-miRNA studies of AD as described earlier, further validating their relevance.

Another major strength is the longitudinal study design, which allowed us to track within-individual miRNA expression changes over time. This approach increases robustness by distinguishing sustained expression differences from transient fluctuations and provides insight into how miRNA profiles evolve during cognitive decline.

### 4.2. Limitations

Despite these strengths, our study has several limitations. First, the sample size within each group (*n* = 23–25) and sex imbalance reduce statistical power and may increase Type I/II error risk; larger and sex-balanced studies are required. Second, participants included only Mexican Americans and non-Hispanic White individuals, omitting populations with well-documented AD disparities [61,62].

Third, metabolic comorbidities were aggregated as a count-based covariate to preserve sample size, but this approach may oversimplify heterogenous clinical effects. Fourth, neuronal EV enrichment using thrombin defibrination and CD171 (L1CAM) immunocapture may alter EV cargo [63] and enable co-isolation of non-neuronal vesicles, as CD171 is expressed by astrocytes, microglia, and peripheral cells [64,65].

Fifth, while cognitive testing supported diagnostic classification, no neuroimaging or CSF biomarkers were available in the parent cohort, preventing neuropathological confirmation and multimodal correlation analyses. Sixth, discrepancies between QIAGEN and DE seq2 pipelines likely reflect differences in normalization and transparency of proprietary algorithms; therefore, conclusions rely on concordant miRNA findings. Seventh, miRNA changes observed here are correlative and may reflect consequences rather than contributors to AD. In particular, mechanistic roles of miR-122-5p and other candidates including regulatory targets relevant to Aβ and tau remain to be defined.

Eighth, limited sample volume prevented qPCR validation, as all extracted RNA was required for sequencing. Finally, the two-year intervals between visits may not have been sufficient to detect strong longitudinal progression, and absence of disease-control groups (e.g., Parkinson’s, frontotemporal dementia) limits claims of AD-specificity.

### 4.3. Future Directions

Future studies will validate key miRNA candidates using qPCR in independent plasma cohorts and assess their presence in AD postmortem brain tissue. Expanding to larger, multi-center, multi-ethnic cohorts will improve generalizability and enable assessment of additive value when combined with established AD biomarkers.

Mechanistic studies are needed to clarify whether NEEV-miRNAs contribute to AD pathogenesis or reflect downstream neurodegeneration, including evaluation of miR-122-5p regulatory networks in relation to Aβ and tau pathology. The longitudinal comparison between the two visits identified miRNAs that were uniquely altered over time, suggesting that NEEV-miRNA dynamics may reflect disease progression. Future studies including more than two time-points and longer follow-up intervals will be essential to define temporal trajectories of miRNA dysregulation and to determine whether specific NEEV-miRNAs ca function as sensitive, progression-based biomarkers in AD.

Lastly, comparative studies across other neurodegenerative disorders will help determine diagnostic specificity and support the development of precise NEEV-miRNA-based biomarkers.

## 5. Conclusions

In summary, our study demonstrates that NEEV-associated miRNAs isolated from plasma NEEVs can distinguish individuals with cognitive impairment from cognitively normal controls and reveal biologically meaningful differences between Mexican American and non-Hispanic White participants. Several differentially expressed miRNAs showed associations with AD-related pathways, vascular and metabolic risk factors, and population-specific genetic influences such as APOE ɛ4, underscoring their relevance to both neurodegeneration and health disparities in AD.

Importantly, the convergence of multiple candidate miRNAs on inflammation and NF-κB-mediated signaling highlights a potentially shared mechanistic axis linking comorbid metabolic disease and AD-related neuroinflammation. These findings support the growing evidence that systemic health factors may influence neurodegenerative trajectories through EV-mediated molecular communication.

Beyond identifying promising biomarker candidates, the longitudinal design of this study provides initial insight into how miRNA cargo changes over time with cognitive decline, an essential step toward developing dynamic EV-based tools for early detection and monitoring. Although further validation in larger and more diverse cohorts is needed, our results lay the groundwork for incorporating population-specific NEEV-miRNA signatures into precision diagnostics for AD.

Continued work to established mechanistic relevance, refine neuronal EV isolation methods, and integrate miRNA signals with established AD biomarkers will be critical for advancing these discoveries toward clinical utility.

## Figures and Tables

**Table 1:** Demographic data for study participants, stratified on cognitive diagnosis.

	CI (AD or MCI)		NC	
Population Group (*n*)	MA (*n* = 24)	NHW (*n* = 24)	MA (*n* = 23)	NHW (*n* = 25)
Metabolic comorbidities, * count 0/1/2/3 comorbidities	13/0/8/3	8/3/8/5	8/3/6/5	14/4/1/6
Gender, count F/count M	14/10	8/16	15/7	10/14
Age, Mean (SD)	70.6 (8.2)	71.2 (5.1)	68.7 (5.4)	72.8 (7.8)
APOE e4 alleles, count 0 alleles/1 allele/2 alleles	9/13/2	10/8/6	17/5/0	13/11/1

Abbreviations: AD, Alzheimer’s Disease; CI (AD/MCI), cognitively impaired group includes both AD and MCI patients without imaging confirmation; NC, Normal Control; MA, Mexican American; NHW, Non-Hispanic White; F/M, Females/Males; Y/N, Yes/No; SD, standard deviation; APOE e4, apolipoprotein ε4 allele. * Metabolic comorbidities include presence of T2D, hyperlipidemia, hypertension.

**Table 2:** CI vs. NC: DE miRNAs common to MAs and NHWs in both QIAGEN and DESeq individual visit analyses.

miRNA	QIAGEN			DESeq		
	Fold Change	FDR *p*-Value	*p*-Value	Fold Change	FDR *p*-Value	*p*-Value
MA–Visit 1						
hsa-miR-122-5p	−2.6589	0.0951	0.006906	−2.6095	1.37 × 10^−6^	1.48793 × 10^−8^
MA–Visit 2						
hsa-miR-122-5p	−3.4067	0.00366	0.0000809	−1.0303	0.084205	0.009356078
hsa-let-7a-5p	1.7354	0.03074	0.001768	0.8062	0.005604	0.000124533
NHW–Visit 1						
hsa-miR-122-5p	−3.5135	0.00019	0.000002973	−1.6541	0.002667	9.30459 × 10^−5^
NHW–Visit 2						
hsa-miR-122-5p	−2.7712	0.00771	0.0001398	−1.6402	0.005497	0.000279485
hsa-let-7a-5p	1.6885	0.0973	0.002646	1.1073	0.002107	5.95071 × 10^−5^

**Table 3:** CI vs. NC: DE miRNAs common to both QIAGEN and DESeq unique to MAs.

miRNA	QIAGEN		DESeq		DESeq Controlled for CV	
	Fold Change	*p*-Value	Fold Change	*p*-Value	Fold Change	*p*-Value
Visit 1						
hsa-miR-26a-5p	2.2595	0.000378	0.8642	0.010922669	0.8726	0.01832371
hsa-let-7f-5p	1.8955	0.000396	0.7564	0.000507709	0.6397	0.006657008
hsa-miR-320b	2.3782	0.001096	0.9056	0.006483572	0.7316	0.045400752
hsa-miR-320c	2.2238	0.001604	0.9066	0.011943306	N/A	N/A
hsa-let-7d-5p	1.7734	0.002541	0.5524	0.011829511	0.5260	0.03158259
hsa-miR-139-5p	2.4935	0.007471	1.1965	0.016015759	N/A	N/A
hsa-miR-155-5p	3.0543	0.007599	1.7612	0.005540475	1.7734	0.009433616
hsa-miR-320d	1.9904	0.009145	0.8377	0.031186727	N/A	N/A
Visit 2						
hsa-miR-26a-5p	3.3986	1.34 × 10^−6^	1.6468	3.22755 × 10^−6^	1.7479	1.77526 × 10^−5^
hsa-miR-98-5p	2.6111	0.000258	1.1289	0.005393586	0.9795	0.038377946
hsa-miR-16-5p	−1.9802	0.000356	−0.8942	9.9069 × 10^−5^	−0.7785	0.002250029
hsa-miR-744-5p	2.7088	0.000648	1.5357	0.002876615	1.5806	0.009644383
hsa-miR-432-5p	3.3424	0.000753	1.3822	0.023778746	N/A	N/A
hsa-miR-25-3p	−2.0924	0.001285	−0.9827	0.003175884	−0.8134	0.033630161
hsa-miR-29c-3p	−2.1563	0.001582	−0.7637	0.046790757	N/A	N/A
hsa-let-7f-5p	1.8117	0.002175	0.8449	0.000388528	0.8964	0.001432407
hsa-let-7e-5p	1.9017	0.002485	0.9063	0.001089766	0.9029	0.005154222
hsa-miR-652-3p	2.9118	0.003041	1.7948	0.008419156	N/A	N/A
hsa-miR-451a	−2.1719	0.003222	−0.8719	0.040413382	N/A	N/A
hsa-miR-181a-5p	2.9416	0.00499	2.0489	0.00157171	1.8985	0.010124267
hsa-miR-382-5p	2.5017	0.006856	1.2004	0.040084269	N/A	N/A
hsa-miR-32-5p	−2.7186	0.006956	−2.0868	0.013834039	−3.0281	0.003596737
hsa-miR-144-3p	−2.0347	0.011515	−1.1413	0.024266645	−1.2024	0.039307561
hsa-miR-26b-5p	1.8652	0.011878	0.8465	0.014921276	0.8327	0.040978652
hsa-miR-139-5p	1.9814	0.014604	1.1088	0.010990028	0.9906	0.048569444
hsa-miR-628-3p	2.2481	0.015016	1.5135	0.030302303	1.8781	0.026313543

Abbreviations: N/A, Differential expression not statistically significant.

**Table 4:** CI vs. NC: DE miRNAs common to both QIAGEN and DESeq unique to NHW.

miRNA	QIAGEN		DESeq		DESeq Controlled for CV	
	Fold Change	*p*-Value	Fold Change	*p*-Value	Fold Change	*p*-Value
Visit 1						
hsa-miR-184	22.8945	1.31 × 10^−12^	2.8623	2.55508 × 10^−5^	2.7454	0.001225839
hsa-miR-486-5p	−2.5550	8.21 × 10^−6^	−1.0808	3.81796 × 10^−5^	−1.1442	1.17529 × 10^−5^
hsa-miR-133a-3p	4.8088	0.000136	3.1374	0.000782628	N/A	N/A
hsa-miR-1260b	3.1867	0.000232	1.7908	0.000977265	1.1588	0.035758812
hsa-miR-125b-5p	4.0213	0.000302	2.3179	0.005661509	2.5050	0.003892795
hsa-miR-1260a	2.7921	0.000428	1.7995	0.000943134	1.1201	0.042932868
hsa-miR-451a	−2.9846	0.000709	−1.5134	0.003312469	−1.3089	0.013690742
hsa-miR-150-5p	−2.3846	0.000724	−1.3585	0.010751915	−1.4706	0.009600561
hsa-miR-30a-3p	−2.5455	0.001874	−1.2236	0.04022529	−1.2556	0.03173225
hsa-miR-4732-5p	−2.8367	0.001951	−1.2917	0.021940416	−1.3245	0.022124385
hsa-miR-629-5p	−1.9755	0.003393	-0.9841	0.006541369	−0.9703	0.008959777
hsa-miR-100-5p	3.0964	0.004191	2.4792	0.000331924	N/A	N/A
hsa-let-7i-5p	−1.5609	0.007761	−0.3969	0.025137485	−0.3864	0.019854645
Visit 2						
hsa-miR-31-5p	15.4400	2.63 × 10^−6^	3.4495	0.027186298	N/A	N/A
hsa-miR-100-5p	7.2821	4.86 × 10^−6^	3.4026	8.0048 × 10^−6^	2.4187	0.002164492
hsa-miR-4674	5.6239	7.35 × 10^−6^	3.0092	0.000164365	2.2139	0.008434245
hsa-miR-133a-3p	6.0388	4.13 × 10^−5^	3.4721	1.53595 × 10^−5^	2.3877	0.00527116
hsa-miR-125b-5p	5.7115	8.03 × 10^−5^	2.1817	0.006523807	N/A	N/A
hsa-miR-101-3p	3.9138	0.000603	2.6056	0.010452733	2.7051	0.015040877
hsa-miR-7-5p	2.5373	0.000813	1.4101	0.003260672	1.4041	0.003602864

Abbreviations: N/A, Expression levels were not statistically significant.

**Table 5:** DE miRNAs in Visit 2 in comparison to Visit 1 within the CI group.

QIAGEN RNA-Seq Portal			DESeq2		
miRNA	Fold Change	*p*-Value	miRNA	Fold Change	*p*-Value
MA with CI					
Overrepresented			Overrepresented		
hsa-miR-499a-5p	21.1916	0.00003385	hsa-miR-324-5p	1.8707	0.00164
			hsa-miR-26a-5p	0.9759	0.00236
			hsa-miR-1307-3p	1.2662	0.01342
			hsa-miR-98-5p	0.9709	0.01389
			hsa-miR-326	2.2103	0.01759
			hsa-miR-744-5p	1.1047	0.02441
			hsa-miR-12136	2.8788	0.03226
			hsa-miR-26b-5p	0.6761	0.03857
			hsa-miR-186-5p	1.8926	0.04413
			hsa-miR-142-3p	0.9846	0.0454
			Underrepresented		
			hsa-miR-184	−2.2055	0.00095
			hsa-miR-1268b	−2.7423	0.00795
			hsa-miR-133b	−3.1213	0.03241
			hsa-miR-4787-5p	−2.4008	0.04179
**NHW with CI**					
Underrepresented			Overrepresented		
hsa-miR-1-3p	−11.80037074	7.598 × 10^−9^	hsa-miR-877-5p	1.419898676	0.01123
hsa-miR-7-5p	−4.325563298	0.0001131	hsa-miR-1908-5p	1.52342791	0.02548
hsa-miR-184	−5.240625245	0.0002988	hsa-miR-483-5p	1.452485598	0.02763
hsa-miR-3182	−5.759964762	0.001183			

## Data Availability

All data and original code generated for analysis will be made available upon reasonable request.

## Supplementary Materials

The following supporting information can be downloaded at: https://www.mdpi.com/article/10.3390/cells14221784/s1, SupplementaryDocuments: Supplementary Document S1, Supplementary Document S2, Supplementary Document S3, Supplementary Document S4.

## Abbreviations

The following abbreviations are used in this manuscript:

DE	Differential Expression
AD	Alzheimer’s disease
CI	Cognitive Impairment
EV	Extracellular Vesicle
NEEV	Neuronal Enriched Extracellular Vesicle
MA	Mexican American
NHW	Non-Hispanic White
