# Supplementary material for: Neuronal Enriched Extracellular Vesicle miR-122-5p as a Potential Biomarker for Alzheimer’s Disease
Source: Cells. 2025 Nov 13;14(22):1784. doi: 10.3390/cells14221784 (PMC12651308; doi:10.3390/cells14221784)
Supplement: Supplementary file 1 [file cells-14-01784-s001.zip › Supplementary Document S1.pdf]

Supplementary Document S1

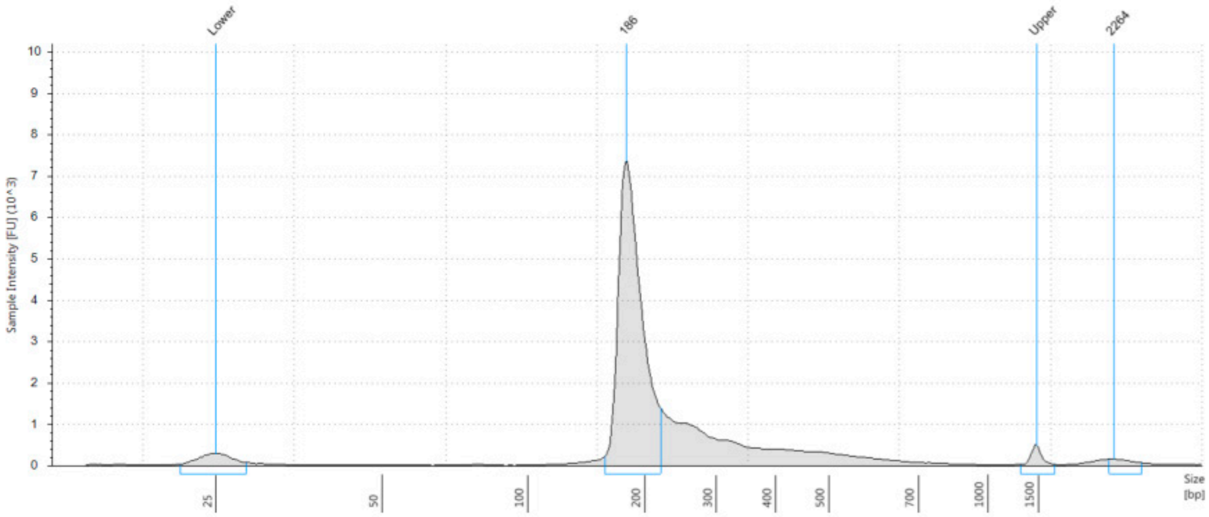

FIGURE S1: miRNA library QC. The library traces taken from Agilent Tape station quality control analysis. The peak length (186bp) indicates the base length of the microRNA library with high purity

Target	Cat#
NCAM1	Thermo Fisher, MA5-31074
PDCD6IP	Thermo Fisher, MA5-38362
ATP1A3	Proteintech, 10868-1-AP
CD81	Abcam, ab109201
CD9	Abcam, ab236630
Goat Anti-Rabbit IgG H&L (HRP)	Abcam, ab97051
Goat Anti-Mouse IgG H&L（HRP）	Abcam, ab205719

TABLE S1: List of primary and secondary antibodies used in the western blot for EV characterization

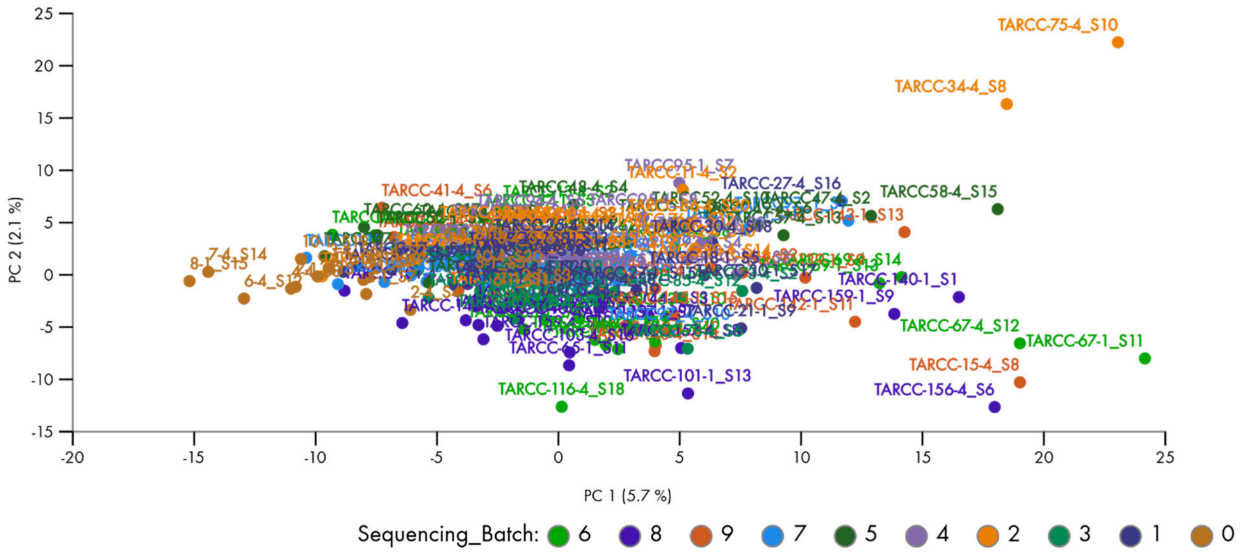

FIGURE S2: Principal component Analysis (PCA); All of the sequencing batches and both visits were included after eliminating the outliers.

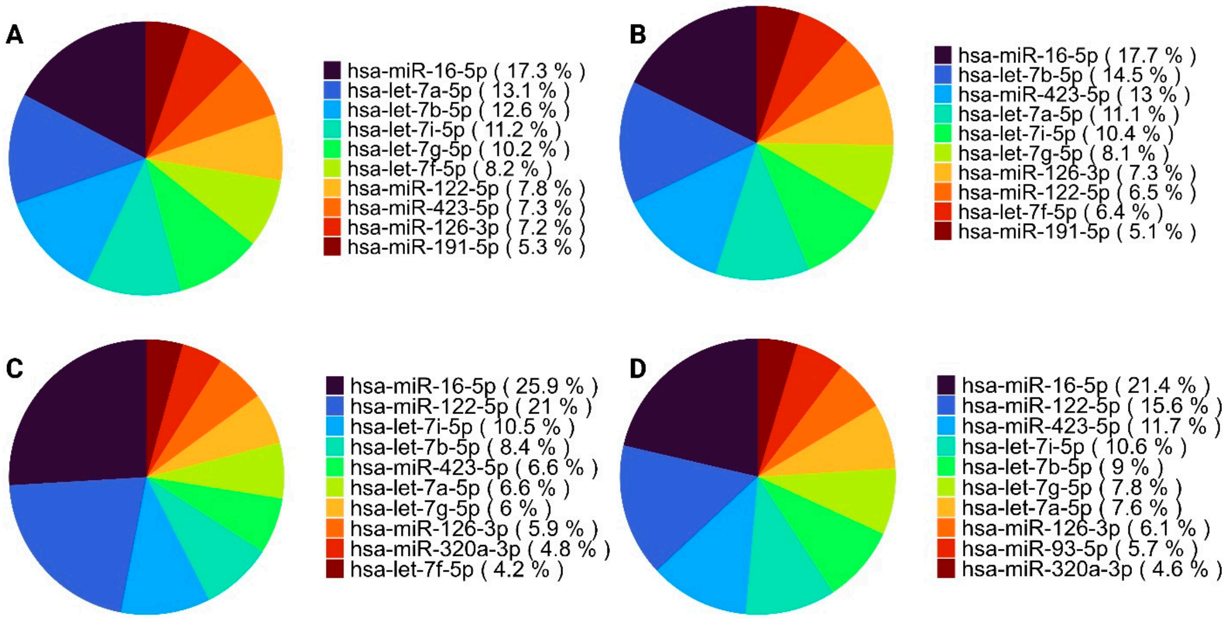

FIGURE S3: The 10 most representative miRNAs in NEEVs in MA-CI (A), MA-NC (C), NHW-CI (B) and NHW-NC (D).

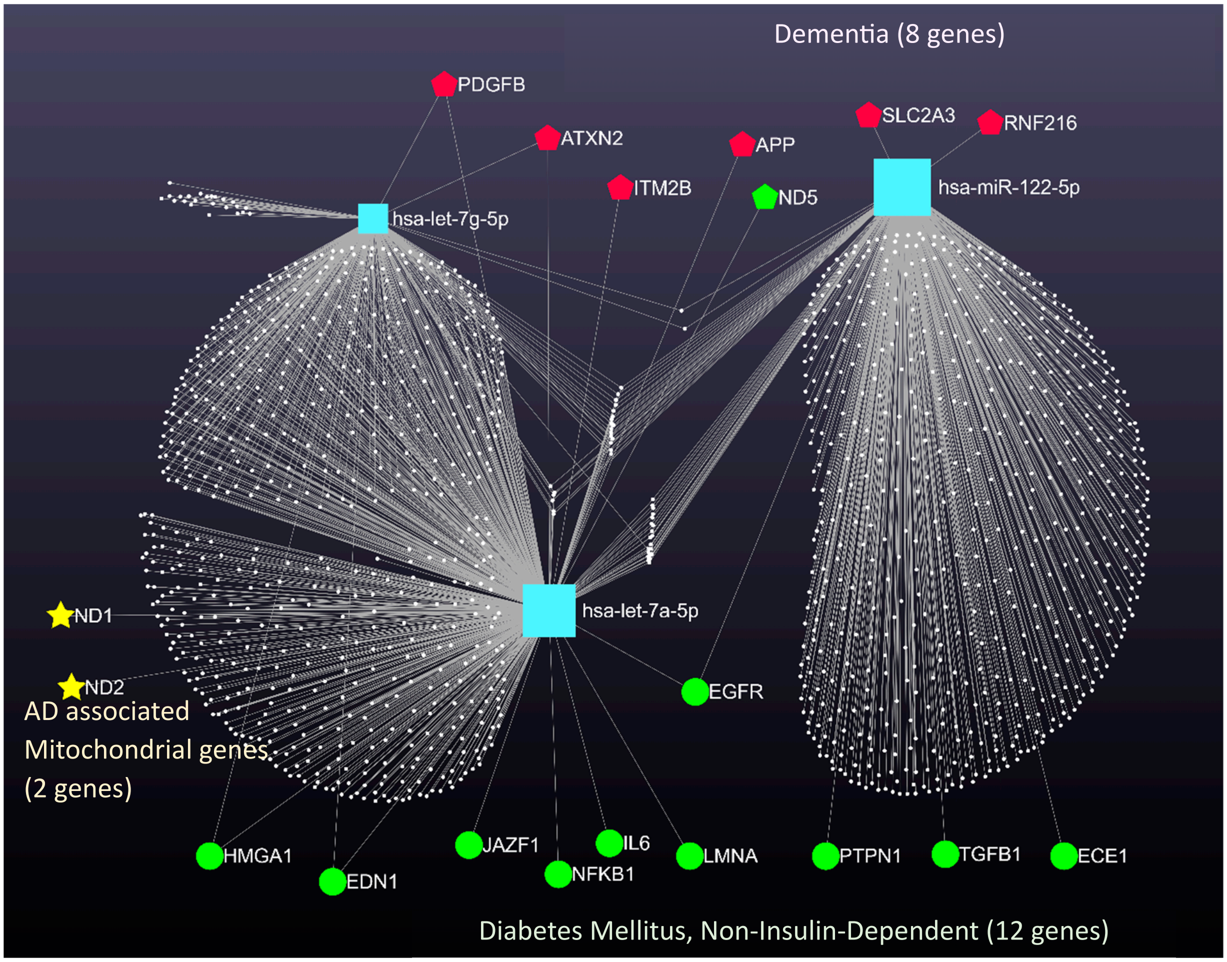

FIGURE S4: miRNet (a miRNA-centric network visual analytics platform, v2.0) for hsa-let-7a-5p, hsa-let-7g-5p and hsa-miR-122-5p. These three miRNAs are interconnected and target genes associated with AD, Dementia and Diabetes Mellitus [31-33].

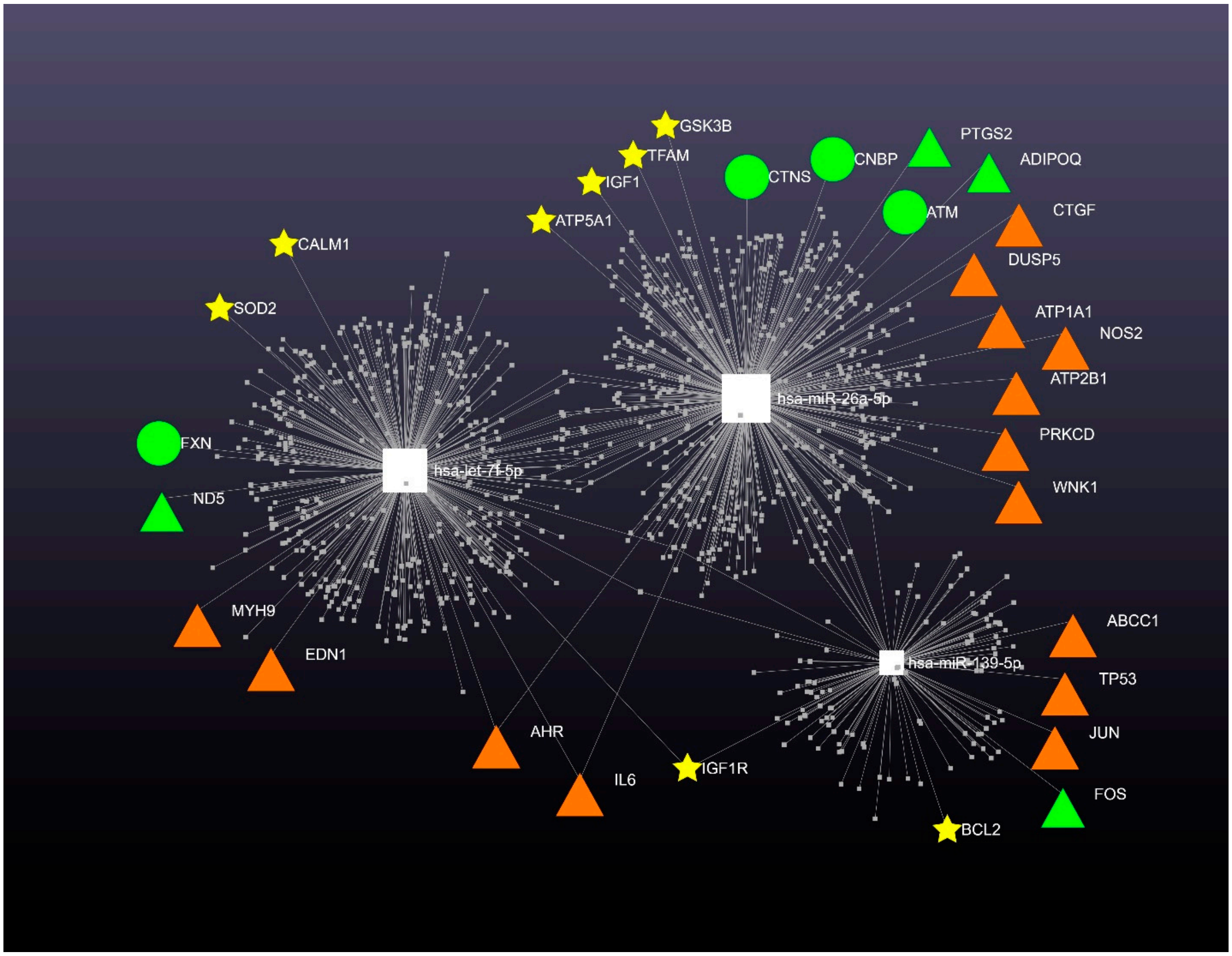

FIGURE S5: miRNet (a miRNA-centric network visual analytics platform, v2.0) for hsa-miR-26a-5p, hsa-let-7f-5p and hsa-miR-139-5p. MA specific DE miRNAs in CI group target 8 genes related to AD (Yellow stars), 18 genes related to Hypertensive disease (Orange triangles) and 8 genes related to Diabetes Mellitus (Green).

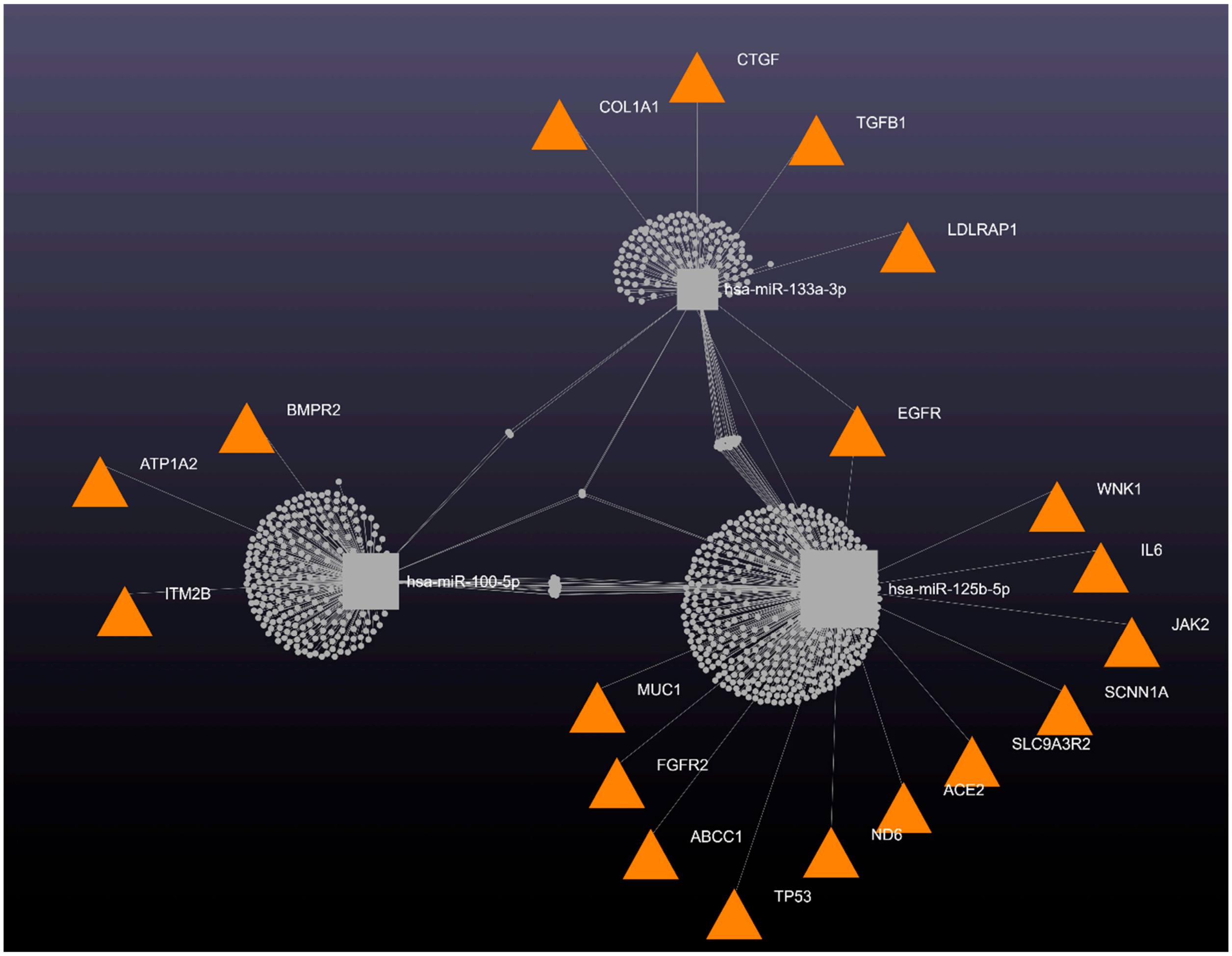

FIGURE S6: miRNet (a miRNA-centric network visual analytics platform, v2.0) for hsa-miR-133a-3p, hsa-miR-125b-5p and hsa-miR-100-5p. NHW specific DE miRNAs in CI group target 19 genes related to Hypertensive disease (orange triangles)

TABLE S2: : hsa-let-7a-5p in NHW: CI vs NC – Visit 2

```
lm(formula = Expression ~ Experimental_Group + Sex + AgeAtVisit +
    APOE4_INDEX + Metabolic_Index, data = miRNA_data)

Residuals:
     Min       1Q   Median       3Q      Max
-1.84011 -0.45955  0.00421  0.37326  1.95415

Coefficients:
                     Estimate Std. Error t value Pr(>|t|)
(Intercept)           8.48887    1.39232   6.097 2.88e-07 ***
Experimental_GroupNC -0.64060    0.25698  -2.493   0.0167 *
```

```
SexMale              -0.25000    0.25371  -0.985   0.3301  
AgeAtVisit            0.01573    0.01900   0.828   0.4122  
APOE4_INDEX           0.37522    0.17504   2.144   0.0379 *
Metabolic_Index       0.22506    0.10097   2.229   0.0312 *
---
Signif. codes:  0 ‘***’ 0.001 ‘**’ 0.01 ‘*’ 0.05 ‘.’ 0.1 ‘ ’ 1
```

TABLE S3: : hsa-let-7a-5p in MA: CI vs NC – Visit 2

```
lm(formula = Expression ~ Experimental_Group + Sex + AgeAtVisit + 
    APOE4_INDEX + Metabolic_Index, data = miRNA_data)

Residuals:
     Min       1Q   Median       3Q      Max 
-2.15576 -0.34974 -0.02991  0.39033  1.38601 

Coefficients:
                      Estimate Std. Error t value Pr(>|t|)    
(Intercept)          10.651986   1.176580   9.053    2e-11 ***
Experimental_GroupNC -0.532774   0.245298  -2.172   0.0356 *  
SexMale               0.342500   0.235749   1.453   0.1537    
AgeAtVisit           -0.001365   0.016188  -0.084   0.9332    
APOE4_INDEX          -0.048937   0.212929  -0.230   0.8193    
Metabolic_Index      -0.026172   0.101340  -0.258   0.7975    
---
Signif. codes:  0 ‘***’ 0.001 ‘**’ 0.01 ‘*’ 0.05 ‘.’ 0.1 ‘ ’ 1
```
