# Supplementary material for: Neuronal Enriched Extracellular Vesicle miR-122-5p as a Potential Biomarker for Alzheimer’s Disease
Source: Cells. 2025 Nov 13;14(22):1784. doi: 10.3390/cells14221784 (PMC12651308; doi:10.3390/cells14221784)
Supplement: Supplementary file 1 [file cells-14-01784-s001.zip › Supplementary Document S2.pdf]

Supplementary Document S2

CI vs NC: Differentially expressed miRNAs between the cognitively impaired (CI) group and the normal control (NC) group for both Mexican Americans (MA) and Non-Hispanic Whites (NHW)

Individual Visit Analyses

Analyses were done both in QIAGEN RNA-seq portal (QIAGEN) and DEseq2 in R programming.

QIAGEN RNA-seq portal: Threshold of False Discovery Rate (FDR) p-value threshold of <=0.1 and the fold change greater than 1.1 or less than -1.1

DEseq2: Threshold of miRNAs with mean count < 10 across samples and the raw p- value <0.05

1. DE miRNAs common to QIAGEN RNA-seq portal, DEseq in R with and without controlling for Covariables

CV: Covariables including Age, presence of *APOE* e4 allele (APOE4_Index), presence of Metabolic comorbidities (Metabolic_Index), Gender (Male/Female)

Table S1: MA Visit 1 only: All DE miRNAs in CI vs NC

miRNA	QIAGEN		DEseq		DEseq controlled for CV	
	Fold Change	P-value	log2Fold Change	P-value	log2Fold Change	P-value
**hsa-let-7a-5p**	2.0738	5.94E-05	0.9366	2.26975E-05	0.7181	0.002487528
**hsa-miR-26a-5p**	2.2595	0.000378	0.8642	0.010922669	0.8726	0.01832371
**hsa-let-7f-5p**	1.8955	0.000396	0.7564	0.000507709	0.6397	0.006657008
**hsa-miR-320b**	2.3782	0.001096	0.9056	0.006483572	0.7316	0.045400752
**hsa-miR-4492**	4.7413	0.001141	N/A	N/A	N/A	N/A
**hsa-miR-320c**	2.2238	0.001604	0.9066	0.011943306	N/A	N/A
**hsa-let-7d-5p**	1.7734	0.002541	0.5524	0.011829511	0.5260	0.03158259
**hsa-let-7g-5p**	1.6923	0.002578	0.6642	0.001840748	0.4750	0.034922442
**hsa-miR-4674**	2.2902	0.005033	N/A	N/A	N/A	N/A
**hsa-miR-26b-5p**	1.8490	0.005789	N/A	N/A	0.7769	0.040178288
**hsa-miR-1180-3p**	3.4409	0.0066	N/A	N/A	N/A	N/A
**hsa-miR-122-5p**	-2.6589	0.006906	-2.6095	1.48793E-08	-2.0191	1.11083E-06
**hsa-miR-139-5p**	2.4935	0.007471	1.1965	0.016015759	N/A	N/A
**hsa-miR-155-5p**	3.0543	0.007599	1.7612	0.005540475	1.7734	0.009433616

**hsa-miR-181a-5p**	2.6280	0.007638	N/A	N/A	N/A	N/A
**hsa-let-7e-5p**	1.9417	0.007845	N/A	N/A	N/A	N/A
**hsa-miR-375-3p**	3.2606	0.008668	N/A	N/A	N/A	N/A
**hsa-miR-320d**	1.9904	0.009145	0.8377	0.031186727	N/A	N/A
**hsa-miR-4508**	2.6836	0.009691	N/A	N/A	N/A	N/A
**hsa-miR-15b-5p**	N/A	N/A	-0.8025	0.000330997	-0.5810	0.004007535
**hsa-let-7b-5p**	N/A	N/A	0.6253	0.010811608	N/A	N/A
**hsa-miR-4448**	N/A	N/A	2.6374	0.016606751	N/A	N/A
**hsa-miR-25-3p**	N/A	N/A	-0.9544	0.017009553	N/A	N/A
**hsa-miR-5010-5p**	N/A	N/A	-2.0339	0.026632023	-3.1314	0.003343942
**hsa-miR-877-5p**	N/A	N/A	-1.0648	0.032456703	-1.6246	0.002800027
**hsa-miR-326**	N/A	N/A	-2.2883	0.040992369	N/A	N/A
**hsa-miR-196a-5p**	N/A	N/A	2.1019	0.042587083	N/A	N/A
**hsa-miR-93-5p**	N/A	N/A	-0.4183	0.04821474	N/A	N/A
**hsa-miR-7-5p**	N/A	N/A	N/A	N/A	-1.0023	0.002853403
**hsa-miR-1-3p**	N/A	N/A	N/A	N/A	-1.7700	0.004918379
**hsa-let-7c-5p**	N/A	N/A	N/A	N/A	0.8467	0.015171433
**hsa-miR-206**	N/A	N/A	N/A	N/A	-2.2773	0.032914663

Table S2: MA Visit 2 only: DE miRNAs in CI vs NC

**miRNA**	**QIAGEN**		**DEseq**		**DEseq controlled for CV**	
	Fold Change	P-value	log2Fold Change	P-value	log2Fold Change	P-value
**hsa-miR-26a-5p**	3.3986	1.34E-06	1.6468	3.22755E-06	1.7479	1.77526E-05
**hsa-miR-15a-5p**	-2.5493	2.91E-05	-0.9740	0.006232953	N/A	N/A
**hsa-let-7c-5p**	2.4912	4.18E-05	1.3187	0.000316008	1.3911	0.000699857
**hsa-miR-499a-5p**	17.3416	6.32E-05	N/A	N/A	N/A	N/A
**hsa-miR-122-5p**	-3.4067	8.09E-05	-1.0303	0.009356078	-1.1677	0.021559905
**hsa-miR-98-5p**	2.6111	0.000258	1.1289	0.005393586	0.9795	0.038377946
**hsa-miR-4492**	6.2474	0.000282	N/A	N/A	N/A	N/A
**hsa-miR-16-5p**	-1.9802	0.000356	-0.8942	9.9069E-05	-0.7785	0.002250029
**hsa-miR-744-5p**	2.7088	0.000648	1.5357	0.002876615	1.5806	0.009644383
**hsa-miR-432-5p**	3.3424	0.000753	1.3822	0.023778746	N/A	N/A
**hsa-miR-25-3p**	-2.0924	0.001285	-0.9827	0.003175884	-0.8134	0.033630161
**hsa-miR-29c-3p**	-2.1563	0.001582	-0.7637	0.046790757	N/A	N/A
**hsa-let-7a-5p**	1.7354	0.001768	0.8062	0.000124533	0.8037	0.001107139
**hsa-let-7f-5p**	1.8117	0.002175	0.8449	0.000388528	0.8964	0.001432407
**hsa-let-7e-5p**	1.9017	0.002485	0.9063	0.001089766	0.9029	0.005154222
**hsa-miR-660-5p**	-1.9711	0.003001	N/A	N/A	N/A	N/A

**hsa-miR-652-3p**	2.9118	0.003041	1.7948	0.008419156	N/A	N/A
**hsa-miR-451a**	-2.1719	0.003222	-0.8719	0.040413382	N/A	N/A
**hsa-miR-181a-5p**	2.9416	0.00499	2.0489	0.00157171	1.8985	0.010124267
**hsa-miR-2110**	-2.0202	0.005172	N/A	N/A	N/A	N/A
**hsa-miR-382-5p**	2.5017	0.006856	1.2004	0.040084269	N/A	N/A
**hsa-miR-32-5p**	-2.7186	0.006956	-2.0868	0.013834039	-3.0281	0.003596737
**hsa-miR-193a-5p**	-2.2167	0.007816	N/A	N/A	N/A	N/A
**hsa-miR-155-5p**	2.4315	0.008101	N/A	N/A	N/A	N/A
**hsa-miR-15b-3p**	-1.9915	0.008343	N/A	N/A	N/A	N/A
**hsa-miR-1304-3p**	3.4236	0.009259	N/A	N/A	N/A	N/A
**hsa-miR-1299**	3.8711	0.011285	N/A	N/A	N/A	N/A
**hsa-miR-144-3p**	-2.0347	0.011515	-1.1413	0.024266645	-1.2024	0.039307561
**hsa-miR-26b-5p**	1.8652	0.011878	0.8465	0.014921276	0.8327	0.040978652
**hsa-miR-4508**	2.6845	0.012422	N/A	N/A	N/A	N/A
**hsa-miR-885-5p**	-3.3102	0.012982	N/A	N/A	N/A	N/A
**hsa-let-7g-5p**	1.4913	0.014111	0.6326	0.000377611	0.6074	0.004023353
**hsa-miR-139-5p**	1.9814	0.014604	1.1088	0.010990028	0.9906	0.048569444
**hsa-miR-628-3p**	2.2481	0.015016	1.5135	0.030302303	1.8781	0.026313543
**hsa-miR-15b-5p**	N/A	N/A	-0.6741	0.005321549	N/A	N/A
**hsa-miR-181a-2-3p**	N/A	N/A	2.9620	0.010437132	3.8786	0.007400934
**hsa-miR-375-3p**	N/A	N/A	-1.8883	0.012713886	-2.0479	0.026744802
**hsa-miR-107**	N/A	N/A	-0.7879	0.014161867	N/A	N/A
**hsa-let-7b-5p**	N/A	N/A	0.6712	0.014563326	N/A	N/A
**hsa-miR-151a-5p**	N/A	N/A	0.6102	0.015505416	N/A	N/A
**hsa-miR-1260a**	N/A	N/A	1.1299	0.016408055	1.1071	0.035951484
**hsa-miR-1260b**	N/A	N/A	0.9687	0.024910108	N/A	N/A
**hsa-miR-142-5p**	N/A	N/A	-0.5583	0.026119837	N/A	N/A
**hsa-miR-12136**	N/A	N/A	3.1911	0.026178592	3.7294	0.043644821
**hsa-let-7b-3p**	N/A	N/A	-2.0985	0.026517284	N/A	N/A
**hsa-miR-184**	N/A	N/A	-1.4032	0.034754247	-1.7414	0.020055082
**hsa-miR-199a-5p**	N/A	N/A	-2.3408	0.039685967	N/A	N/A
**hsa-miR-30e-5p**	N/A	N/A	-0.4767	0.039928668	N/A	N/A
**hsa-miR-29a-3p**	N/A	N/A	-0.4484	0.040197397	N/A	N/A
**hsa-miR-17-5p**	N/A	N/A	1.1972	0.044607461	N/A	N/A
**hsa-miR-4758-5p**	N/A	N/A	-1.1263	0.046337179	-1.8630	0.00487304
**hsa-miR-1294**	N/A	N/A	-1.3681	0.048902538	N/A	N/A
**hsa-miR-128-3p**	N/A	N/A	1.2509	0.049621298	1.4980	0.032851181
**hsa-miR-4433b-5p**	N/A	N/A	N/A	N/A	1.9155	0.038845763
**hsa-miR-197-3p**	N/A	N/A	N/A	N/A	1.8854	0.04800532

Table S3: NHW Visit 1 only: DE miRNAs in CI vs NC

miRNA	QIAGEN		DEseq		DEseq controlled for CV	
	Fold Change	P-value	log2Fold Change	P-value	log2Fold Change	P-value
**hsa-miR-184**	22.8945	1.312E-12	2.8623	2.55508E-05	2.7454	0.00123
**hsa-miR-7-5p**	7.7655	1.623E-07	N/A	N/A	2.1950	0.0001
**hsa-miR-122-5p**	-3.5135	0.000002973	-1.6541	9.30459E-05	-1.3735	0.00068
**hsa-miR-486-5p**	-2.5550	0.000008209	-1.0808	3.81796E-05	-1.1442	1.2E-05
**hsa-miR-4674**	4.3840	0.000008482	2.9703	5.44972E-05	2.2058	0.00341
**hsa-miR-133a-3p**	4.8088	0.0001364	3.1374	0.000782628	N/A	N/A
**hsa-miR-34c-5p**	-31.4911	0.0001661	N/A	N/A	N/A	N/A
**hsa-miR-1260b**	3.1867	0.0002318	1.7908	0.000977265	1.1588	0.03576
**hsa-miR-125b-5p**	4.0213	0.0003018	2.3179	0.005661509	2.5050	0.00389
**hsa-miR-206**	-5.7964	0.0003538	N/A	N/A	-1.7151	0.04527
**hsa-miR-1260a**	2.7921	0.0004284	1.7995	0.000943134	1.1201	0.04293
**hsa-miR-451a**	-2.9846	0.0007091	-1.5134	0.003312469	-1.3089	0.01369
**hsa-miR-150-5p**	-2.3846	0.0007236	-1.3585	0.010751915	-1.4706	0.0096
**hsa-miR-30a-3p**	-2.5455	0.001874	-1.2236	0.04022529	-1.2556	0.03173
**hsa-miR-4732-5p**	-2.8367	0.001951	-1.2917	0.021940416	-1.3245	0.02212
**hsa-miR-193b-5p**	-4.2995	0.002044	N/A	N/A	-1.7588	0.03336
**hsa-miR-629-5p**	-1.9755	0.003393	-0.9841	0.006541369	-0.9703	0.00896
**hsa-miR-100-5p**	3.0964	0.004191	2.4792	0.000331924	N/A	N/A
**hsa-let-7i-5p**	-1.5609	0.007761	-0.3969	0.025137485	-0.3864	0.01985
**hsa-miR-877-5p**	-2.1754	0.010954209	N/A	N/A	N/A	N/A
**hsa-miR-320a-3p**	-1.5346	0.011325802	N/A	N/A	-0.4178	0.0408
**hsa-miR-4497**	-2.9445	0.011590859	N/A	N/A	N/A	N/A
**hsa-let-7g-5p**	-1.4987	0.011954556	N/A	N/A	N/A	N/A
**hsa-miR-532-3p**	-3.3811	0.012396709	N/A	N/A	N/A	N/A
**hsa-miR-1-3p**	3.3965	0.012458044	1.3559	0.023108334	N/A	N/A
**hsa-miR-4758-5p**	N/A	N/A	3.2434	4.9839E-06	1.9254	0.00609
**hsa-miR-8485**	N/A	N/A	3.0806	8.05965E-06	N/A	N/A
**hsa-let-7c-5p**	N/A	N/A	1.1435	0.000593043	1.2258	0.00017
**hsa-let-7a-5p**	N/A	N/A	1.0540	0.001152873	0.8001	0.00922
**hsa-miR-4448**	N/A	N/A	3.3099	0.003649933	N/A	N/A
**hsa-miR-574-5p**	N/A	N/A	1.0681	0.004262209	N/A	N/A
**hsa-miR-133b**	N/A	N/A	2.9909	0.004867668	N/A	N/A
**hsa-miR-31-5p**	N/A	N/A	2.8166	0.015609174	N/A	N/A
**hsa-miR-9-5p**	N/A	N/A	3.8735	0.016090398	N/A	N/A
**hsa-miR-130b-3p**	N/A	N/A	-0.5553	0.035086515	N/A	N/A
**hsa-miR-151b**	N/A	N/A	-0.6747	0.040676486	-0.7097	0.03065
**hsa-miR-375-3p**	N/A	N/A	1.2674	0.040826957	N/A	N/A
**hsa-miR-205-5p**	N/A	N/A	N/A	N/A	1.7013	0.00881

**hsa-miR-483-5p**	N/A	N/A	N/A	N/A	-1.6300	0.03048

Table S4: NHW Visit 2 only: DE miRNAs in CI vs NC

miRNA	QIAGEN		DEseq		DEseq controlled for CV	
	Fold Change	P-value	log2Fold Change	P-value	log2Fold Change	P-value
**hsa-miR-1260b**	4.7414	0.000001603	2.3191	7.3687E-05	1.5694	0.010232
**hsa-miR-31-5p**	15.4400	0.000002626	3.4495	0.027186298	N/A	N/A
**hsa-miR-1260a**	4.7440	0.000002829	2.3622	0.000342645	1.5064	0.02883
**hsa-miR-100-5p**	7.2821	0.000004862	3.4026	8.0048E-06	2.4187	0.002164
**hsa-miR-4674**	5.6239	0.00000735	3.0092	0.000164365	2.2139	0.008434
**hsa-miR-133a-3p**	6.0388	0.0000413	3.4721	1.53595E-05	2.3877	0.005271
**hsa-miR-125b-5p**	5.7115	0.00008027	2.1817	0.006523807	N/A	N/A
**hsa-miR-122-5p**	-2.7712	0.0001398	-1.6402	0.000279485	-1.1570	0.006312
**hsa-miR-184**	4.3026	0.0003384	2.6236	0.000407254	1.8960	0.010581
**hsa-miR-101-3p**	3.9138	0.0006031	2.6056	0.010452733	2.7051	0.015041
**hsa-miR-7-5p**	2.5373	0.0008131	1.4101	0.003260672	1.4041	0.003603
**hsa-let-7a-5p**	1.6885	0.002646	1.1073	5.95071E-05	0.6956	0.009574
**hsa-miR-4758-5p**	N/A	N/A	3.4091	7.37441E-07	2.1887	0.002596
**hsa-miR-8485**	N/A	N/A	3.7627	1.31693E-06	1.8719	0.020363
**hsa-let-7c-5p**	N/A	N/A	1.4081	0.000202893	0.9724	0.006443
**hsa-miR-574-5p**	N/A	N/A	1.5202	0.000557694	N/A	N/A
**hsa-let-7g-5p**	N/A	N/A	-0.4880	0.002889346	-0.4820	0.005854
**hsa-miR-205-3p**	N/A	N/A	3.3456	0.006158853	2.6655	0.046604
**hsa-miR-133b**	N/A	N/A	3.0073	0.007385247	2.5440	0.037948
**hsa-let-7b-5p**	N/A	N/A	0.7246	0.008701518	0.5604	0.036054
**hsa-miR-151a-5p**	N/A	N/A	-0.7063	0.013193751	-0.6278	0.025858
**hsa-miR-4448**	N/A	N/A	3.2860	0.01364363	2.8576	0.04155
**hsa-miR-1-3p**	N/A	N/A	1.2883	0.020301958	1.3479	0.015764
**hsa-miR-375-3p**	N/A	N/A	1.5729	0.047160134	N/A	N/A
**hsa-miR-107**	N/A	N/A	0.6485	0.049576815	N/A	N/A
**hsa-miR-15a-5p**	N/A	N/A	N/A	N/A	0.9833	0.018055

Table S5: All DE miRNAs in CI vs NC Common to both MAs and NHWs

miRNA	MA		NHW	
	Fold Change	P-value	Fold Change	P-value

Visit 1: QIAGEN Analysis				
**hsa-let-7g-5p**	1.6923	0.002578	-1.4987	0.011954556
**hsa-miR-4674**	2.2902	0.005033	4.3840	0.000008482
**hsa-miR-122-5p**	-2.6589	0.006906	-3.5135	0.000002973
Visit 1: DESeq Analysis				
**hsa-let-7a-5p**	0.9366	2.27E-05	1.0540	0.001152873
**hsa-miR-122-5p**	-2.6095	1.488E-08	-1.6541	9.30459E-05
**hsa-miR-4448**	2.6374	0.0166068	3.3099	0.003649933
Visit 2: QIAGEN Analysis				
**hsa-miR-122-5p**	-3.4067	8.09E-05	-2.7712	0.0001398
**hsa-let-7a-5p**	1.7354	0.001768	1.6885	0.002646
Visit 2: DESeq Analysis				
**hsa-let-7c-5p**	1.3187	0.000316	1.4081	0.000202893
**hsa-miR-122-5p**	-1.0303	0.0093561	-1.6402	0.000279485
**hsa-let-7a-5p**	0.8062	0.0001245	1.1073	5.95071E-05
**hsa-let-7g-5p**	0.6326	0.0003776	-0.4880	0.002889346
**hsa-miR-375-3p**	-1.8883	0.0127139	1.5729	0.047160134
**hsa-miR-107**	-0.7879	0.0141619	0.6485	0.049576815
**hsa-let-7b-5p**	0.6712	0.0145633	0.7246	0.008701518
**hsa-miR-151a-5p**	0.6102	0.0155054	-0.7063	0.013193751
**hsa-miR-1260a**	1.1299	0.0164081	2.3622	0.000342645
**hsa-miR-1260b**	0.9687	0.0249101	2.3191	7.3687E-05
**hsa-miR-184**	-1.4032	0.0347542	2.6236	0.000407254
**hsa-miR-4758-5p**	-1.1263	0.0463372	3.4091	7.37441E-07

Differential expression analysis including both visits

QIAGEN RNA-seq portal: Repeated measures were not accounted for. The Visit is controlled. Both visits per individual were included. Threshold of False Discovery Rate (FDR) p-value threshold of <=0.1 and the fold change greater than 1.1 or less than -1.1

Table S6: CI vs NC. Common miRNAs for both NHW and MA in QIAGEN without controlling for the covariables.

miRNA	NHW			MA		
	Fold change	FDR p-value	P-value	Fold change	FDR p-value	P-value
**hsa-miR-4674**	4.422874	1.07E-06	1.46E-08	1.624752	0.074756	0.014752

**hsa-miR-122-5p**	-3.00954	2.33E-06	4.69E-08	-2.81622	0.000576	3.32E-05
**hsa-let-7g-5p**	-1.45823	0.006291	0.000429	1.667666	0.00052	2.55E-05
**hsa-let-7a-5p**	1.523416	0.019984	0.001529	1.963905	1.04E-05	1.5E-07
**hsa-miR-151a-5p**	-1.55738	0.019984	0.001632	1.401541	0.085861	0.018531

DREAM analysis

Differential expression testing with linear mixed models for repeated measures

Dream analysis was used to account for the repeated measures when using the datasets containing both the visits for each individual

DREAM: Filtered by the raw p- value <0.05.

CV: Covariables including Age, presence of APOE4 allele (APOE4_Index), presence of Metabolic comorbidities (Metabolic_Index), Gender (Male/Female)

Table S7: CI vs NC. Common miRNAs for both NHW and MA with Dream analysis without controlling for the covariables.

	**NHW**			**MA**		
**miRNA**	logFC	P.Value	adj.P.Val	logFC	P.Value	adj.P.Val
**hsa-miR-6515-5p**	1.309781	2.31E-05	0.001428	-0.59514	0.028117	0.205152
**hsa-miR-199a-5p**	1.609544	7.01E-05	0.001873	-0.94228	0.000725	0.032697
**hsa-let-7i-3p**	1.520819	9.92E-05	0.001873	-0.59141	0.00833	0.109394
**hsa-miR-4665-5p**	1.460256	0.000105	0.001873	-0.47151	0.037054	0.235472
**hsa-let-7g-3p**	1.465153	0.000149	0.001873	-0.80751	0.000261	0.017166
**hsa-miR-106a-5p**	1.411409	0.000199	0.002046	-0.51222	0.034299	0.232995
**hsa-miR-1294**	1.476543	0.000222	0.002113	-0.72995	0.034978	0.23358
**hsa-miR-98-3p**	1.342657	0.000285	0.002276	-0.64499	0.006699	0.094264
**hsa-miR-7-1-3p**	1.271636	0.000296	0.002276	-0.62597	0.011821	0.125883
**hsa-miR-21-3p**	1.295106	0.000351	0.002503	-0.564	0.010437	0.117496
**hsa-miR-320e**	1.165284	0.000407	0.002687	-0.61039	0.015124	0.148972
**hsa-miR-6877-5p**	1.337153	0.000485	0.00294	-0.93581	9.15E-05	0.01392
**hsa-miR-7113-5p**	1.508184	0.00051	0.00294	-0.58581	0.030694	0.215957
**hsa-let-7f-1-3p**	1.349351	0.000645	0.00294	-0.84496	0.00065	0.032697
**hsa-miR-381-3p**	1.337787	0.00072	0.002988	-0.44096	0.047138	0.258878
**hsa-miR-19a-3p**	1.417723	0.000933	0.003327	-0.86742	0.000131	0.01392
**hsa-let-7a-3p**	1.400138	0.001127	0.00363	-1.06683	0.000157	0.01392
**hsa-miR-1303**	1.337229	0.001132	0.00363	-0.69393	0.001949	0.051187
**hsa-miR-3613-5p**	1.32593	0.001181	0.003714	-0.72399	0.00321	0.074397
**hsa-miR-192-5p**	1.215705	0.001425	0.004163	-0.99523	0.013134	0.13618
**hsa-miR-3611**	1.237589	0.001446	0.004191	-0.85749	0.000747	0.032697

**hsa-miR-7847-3p**	1.357394	0.00168	0.004516	-0.46494	0.049946	0.265927
**hsa-miR-885-5p**	1.231249	0.001717	0.004517	-0.70448	0.009996	0.117496
**hsa-miR-3928-3p**	1.091063	0.002027	0.005088	-0.49421	0.022073	0.193262
**hsa-miR-195-5p**	1.162974	0.002172	0.005301	-0.80716	0.000982	0.038693
**hsa-miR-2278**	1.080848	0.003221	0.007024	-0.73971	0.001183	0.042387
**hsa-miR-3149**	1.341912	0.003238	0.007024	-0.65206	0.013662	0.138022
**hsa-miR-376b-3p**	1.184701	0.003748	0.007812	-0.64153	0.011146	0.121984
**hsa-miR-32-5p**	1.141407	0.004666	0.009211	-0.84992	0.006238	0.094264
**hsa-let-7c-5p**	0.833584	0.005786	0.010896	0.941748	0.001727	0.048595
**hsa-miR-627-5p**	1.095948	0.006741	0.012441	-0.65355	0.021685	0.193262
**hsa-miR-19b-3p**	1.277699	0.007286	0.013147	-1.10034	0.004391	0.086494
**hsa-miR-651-5p**	1.016122	0.010241	0.017509	-0.59035	0.04087	0.246279
**hsa-miR-760**	0.987368	0.010291	0.017514	-0.56464	0.025899	0.205152
**hsa-miR-431-3p**	0.851694	0.012622	0.020999	-0.77155	0.001307	0.04291
**hsa-miR-5010-5p**	0.912084	0.015508	0.025234	-0.77667	0.041255	0.246279
**hsa-miR-122-5p**	-1.02404	0.021588	0.033805	-1.43108	0.006097	0.094264
**hsa-miR-181a-5p**	0.870568	0.028313	0.042874	0.983468	0.00581	0.094264
**hsa-miR-376c-3p**	0.89943	0.047995	0.069015	-0.59132	0.036117	0.235472

Table S8: CI vs NC. DE miRNAs unique to MAs in Dream analysis with and without controlling for the covariables (Filtered by raw p-value <0.05)

**miRNA**	**DREAM**			**DREAM controlled for CV**		
	logFC	P.Value	adj.P.Val	logFC	P.Value	adj.P.Val
**hsa-let-7e-5p**	1.1427	0.000101	0.01392	1.1057	0.000787	0.056385
**hsa-miR-26a-5p**	1.3499	0.000177	0.01392	1.3955	0.0009	0.056385
**hsa-miR-139-5p**	1.3678	0.001425	0.043177	1.1128	0.018717	0.206413
**hsa-miR-15b-5p**	-0.7235	0.002515	0.06194	#N/A	#N/A	#N/A
**hsa-miR-3688-3p**	-0.5953	0.003895	0.085253	-0.7235	0.001742	0.057196
**hsa-miR-132-3p**	-0.6497	0.004183	0.086494	-0.7751	0.002046	0.061838
**hsa-miR-25-3p**	-1.0533	0.005968	0.094264	#N/A	#N/A	#N/A
**hsa-let-7a-5p**	0.7007	0.006201	0.094264	0.6321	0.030287	0.222497
**hsa-let-7f-5p**	0.6584	0.006401	0.094264	0.6357	0.023644	0.211098
**hsa-miR-26b-5p**	0.9320	0.006605	0.094264	0.8198	0.031822	0.225579
**hsa-miR-4669**	-0.6128	0.007915	0.107532	-0.6127	0.02091	0.206413
**hsa-miR-1260b**	0.9815	0.00945	0.117496	1.2173	0.002841	0.066749
**hsa-miR-154-5p**	-0.8645	0.009602	0.117496	-0.8177	0.025105	0.211098
**hsa-let-7g-5p**	0.5883	0.010162	0.117496	0.5514	0.032062	0.225579
**hsa-miR-15a-5p**	-0.7929	0.017057	0.163915	#N/A	#N/A	#N/A
**hsa-miR-379-5p**	-0.5484	0.0184	0.172605	-0.6041	0.024747	0.211098
**hsa-miR-92b-5p**	-0.6260	0.018907	0.173241	-0.8079	0.004912	0.087961
**hsa-miR-4747-5p**	-0.5384	0.022733	0.194715	-0.5499	0.040423	0.265446
**hsa-miR-92a-1-5p**	-0.5353	0.023693	0.198614	-0.6533	0.012992	0.168608

**hsa-miR-744-5p**	0.9106	0.026375	0.205152	0.9605	0.042363	0.265843
**hsa-miR-96-5p**	-0.5079	0.026517	0.205152	-0.6173	0.018511	0.206413
**hsa-let-7b-5p**	0.4924	0.026762	0.205152	#N/A	#N/A	#N/A
**hsa-miR-1260a**	1.0338	0.027892	0.205152	1.2181	0.019517	0.206413
**hsa-miR-30e-3p**	0.7393	0.027962	0.205152	#N/A	#N/A	#N/A
**hsa-miR-155-5p**	1.0206	0.030071	0.215418	#N/A	#N/A	#N/A
**hsa-miR-766-5p**	-0.4938	0.033271	0.229977	-0.7710	0.002197	0.061838
**hsa-miR-425-3p**	-0.9225	0.036798	0.235472	#N/A	#N/A	#N/A
**hsa-miR-144-3p**	-0.9948	0.038112	0.238354	#N/A	#N/A	#N/A
**hsa-miR-769-5p**	-0.4299	0.040581	0.246279	-0.5883	0.012317	0.167336
**hsa-miR-365a-3p**	-0.4878	0.043853	0.254088	-0.5426	0.045733	0.273013
**hsa-miR-365b-3p**	-0.4878	0.043853	0.254088	-0.5426	0.045733	0.273013
**hsa-miR-98-5p**	0.8913	0.04593	0.258878	#N/A	#N/A	#N/A
**hsa-miR-4510**	-0.4660	0.046827	0.258878	-0.5556	0.039712	0.265446
**hsa-miR-150-5p**	0.8756	0.047308	0.258878	#N/A	#N/A	#N/A
**hsa-miR-4738-3p**	-0.4473	0.0481	0.25961	-0.5516	0.03004	0.222497

Table S9: CI vs NC. DE miRNAs unique to NHWs in Dream analysis with and without controlling for the covariables (Filtered by raw p-value <0.05)

**miRNA**	**DREAM**			**DREAM controlled for CV**		
	logFC	P.Value	adj.P.Val	logFC	P.Value	adj.P.Val
**hsa-miR-3679-5p**	1.3886	2.18E-06	0.000809	1.2901	3.85526E-05	0.008015
**hsa-miR-99b-3p**	1.4697	7.04E-06	0.000944	1.3805	6.48151E-05	0.008015
**hsa-miR-5187-5p**	1.5996	9.08E-06	0.000944	1.3437	0.000349681	0.010609
**hsa-miR-1275**	1.6618	1.02E-05	0.000944	1.5167	4.44569E-05	0.008015
**hsa-miR-9-5p**	1.8817	2.17E-05	0.001428	1.6304	0.000153825	0.009512
**hsa-miR-200a-3p**	1.6312	2.98E-05	0.00158	1.4472	0.00013547	0.009512
**hsa-miR-548j-5p**	1.5401	4.78E-05	0.001873	1.3512	0.000283952	0.010609
**hsa-miR-3150b-3p**	1.6661	5.29E-05	0.001873	1.3969	0.00055375	0.010609
**hsa-miR-590-5p**	1.4643	5.92E-05	0.001873	1.3572	0.000194511	0.010309
**hsa-miR-299-3p**	1.5616	6.13E-05	0.001873	1.4961	0.000145847	0.009512
**hsa-miR-374a-3p**	1.5785	6.94E-05	0.001873	1.4108	0.00042309	0.010609
**hsa-miR-503-5p**	1.5389	8.46E-05	0.001873	1.4293	0.000557152	0.010609
**hsa-miR-548d-5p**	1.6110	9.18E-05	0.001873	1.4289	0.000749507	0.010609
**hsa-miR-4667-5p**	1.4707	0.000106	0.001873	1.2546	0.000886428	0.010658
**hsa-miR-548ad-5p**	1.5926	0.000106	0.001873	1.3686	0.000772069	0.010609
**hsa-miR-548ae-5p**	1.5926	0.000106	0.001873	1.3686	0.000772069	0.010609
**hsa-miR-1224-5p**	1.4351	0.000117	0.001873	1.1539	0.000708879	0.010609
**hsa-miR-548ay-5p**	1.5867	0.000118	0.001873	1.3884	0.000999416	0.010658
**hsa-miR-27b-5p**	1.4917	0.000128	0.001873	1.2470	0.000511531	0.010609
**hsa-let-7b-3p**	1.3875	0.000133	0.001873	1.1850	0.000913393	0.010658
**hsa-miR-4750-5p**	1.2821	0.000134	0.001873	0.9865	0.002609663	0.015147

**hsa-miR-374b-5p**	1.5133	0.000134	0.001873	1.3633	0.000540035	0.010609
**hsa-miR-374b-3p**	1.4240	0.000145	0.001873	1.1961	0.000991322	0.010658
**hsa-miR-7111-5p**	1.7791	0.000151	0.001873	1.4458	0.001149131	0.010658
**hsa-miR-654-3p**	1.4414	0.000158	0.001873	1.3522	0.000733101	0.010609
**hsa-miR-1306-5p**	1.1303	0.000159	0.001873	0.9450	0.004112054	0.015654
**hsa-miR-127-3p**	1.4708	0.000162	0.001873	1.2782	0.000764173	0.010609
**hsa-miR-326**	1.3799	0.00017	0.001914	1.3886	0.000492671	0.010609
**hsa-miR-6124**	1.5278	0.000186	0.002032	1.2892	0.001123463	0.010658
**hsa-miR-664b-5p**	1.3969	0.000193	0.002046	1.1851	0.000404548	0.010609
**hsa-miR-493-3p**	1.4059	0.000207	0.00208	1.2111	0.001137419	0.010658
**hsa-miR-7977**	1.3949	0.000216	0.002109	1.1555	0.001415943	0.01204
**hsa-miR-590-3p**	1.4469	0.000231	0.002138	1.3241	0.001075298	0.010658
**hsa-miR-10a-3p**	1.4847	0.000237	0.002146	1.2746	0.001040214	0.010658
**hsa-miR-3150a-3p**	1.4510	0.000243	0.00215	1.2588	0.001215302	0.010997
**hsa-miR-6089**	1.5236	0.000261	0.002202	1.2295	0.001752336	0.012551
**hsa-miR-625-3p**	1.4074	0.000267	0.002202	1.2394	0.001672545	0.012551
**hsa-miR-491-5p**	1.3895	0.000267	0.002202	1.2068	0.00146897	0.012111
**hsa-miR-652-5p**	1.4324	0.000295	0.002276	1.2244	0.001619096	0.012551
**hsa-miR-3940-3p**	1.3545	0.000301	0.002276	1.1422	0.001821124	0.012748
**hsa-miR-500a-3p**	1.4405	0.000327	0.002425	1.1860	0.001524547	0.012296
**hsa-miR-148b-3p**	1.3886	0.00035	0.002503	1.1931	0.002104474	0.014196
**hsa-miR-197-5p**	1.3314	0.000392	0.002687	1.0775	0.003885581	0.015595
**hsa-miR-4787-5p**	1.3906	0.000393	0.002687	1.1250	0.001752345	0.012551
**hsa-miR-378d**	1.3717	0.000413	0.002687	1.0934	0.001671432	0.012551
**hsa-miR-411-5p**	1.4572	0.000419	0.002687	1.4537	0.000947852	0.010658
**hsa-miR-16-2-3p**	1.2987	0.00042	0.002687	1.0304	0.006363166	0.018159
**hsa-miR-152-3p**	1.4192	0.00043	0.002707	1.2327	0.001333658	0.011781
**hsa-miR-4492**	1.3871	0.000466	0.002881	1.0858	0.002742335	0.015147
**hsa-miR-423-3p**	1.3927	0.000496	0.00294	1.2452	0.002349969	0.015036
**hsa-miR-6511b-3p**	1.2548	0.000522	0.00294	1.0288	0.003576892	0.015147
**hsa-miR-454-3p**	1.4023	0.000523	0.00294	1.2365	0.002391241	0.015036
**hsa-miR-6741-5p**	1.2867	0.000536	0.00294	1.0054	0.003751261	0.015351
**hsa-let-7f-2-3p**	1.3106	0.000558	0.00294	1.0913	0.002940282	0.015147
**hsa-miR-3615**	1.4030	0.000562	0.00294	1.1614	0.003276609	0.015147
**hsa-miR-378c**	1.4982	0.000572	0.00294	1.2468	0.002554839	0.015147
**hsa-miR-6735-5p**	1.3809	0.000572	0.00294	1.0714	0.004916705	0.01689
**hsa-miR-205-3p**	1.6831	0.000581	0.00294	1.4149	0.005635903	0.017174
**hsa-miR-874-3p**	1.3847	0.000582	0.00294	1.1934	0.003592812	0.015147
**hsa-miR-125b-5p**	2.2100	0.000582	0.00294	1.8551	0.003014484	0.015147
**hsa-miR-222-3p**	1.3866	0.000592	0.00294	1.1989	0.003282857	0.015147
**hsa-miR-140-5p**	1.3955	0.000609	0.00294	1.1327	0.004501023	0.016533
**hsa-miR-6764-5p**	1.2586	0.000625	0.00294	1.0224	0.005193622	0.017174
**hsa-miR-6512-5p**	1.3745	0.000638	0.00294	1.1267	0.003765326	0.015351
**hsa-miR-1250-5p**	1.3260	0.00064	0.00294	1.1472	0.002651249	0.015147

**hsa-miR-369-3p**	1.3490	0.000659	0.00294	1.2078	0.002249129	0.0149
**hsa-miR-22-5p**	1.3682	0.000661	0.00294	1.1561	0.00238076	0.015036
**hsa-miR-1255b-5p**	1.3160	0.000674	0.00294	1.0493	0.002617553	0.015147
**hsa-miR-518e-5p**	1.2302	0.000689	0.00294	0.9747	0.003487698	0.015147
**hsa-miR-519b-5p**	1.2302	0.000689	0.00294	0.9747	0.003487698	0.015147
**hsa-miR-519c-5p**	1.2302	0.000689	0.00294	0.9747	0.003487698	0.015147
**hsa-miR-522-5p**	1.2302	0.000689	0.00294	0.9747	0.003487698	0.015147
**hsa-miR-523-5p**	1.2302	0.000689	0.00294	0.9747	0.003487698	0.015147
**hsa-miR-340-5p**	1.4251	0.000723	0.002988	1.2634	0.002785998	0.015147
**hsa-miR-4674**	2.4269	0.000751	0.002988	2.2643	0.001066755	0.010658
**hsa-miR-548c-5p**	1.3223	0.000751	0.002988	1.0292	0.005382559	0.017174
**hsa-miR-548o-5p**	1.3223	0.000751	0.002988	1.0292	0.005382559	0.017174
**hsa-miR-93-3p**	1.2055	0.000754	0.002988	0.9899	0.004177184	0.015654
**hsa-miR-3125**	1.4067	0.000757	0.002988	1.1119	0.005122879	0.017174
**hsa-miR-4659b-5p**	1.3770	0.000801	0.003127	1.1683	0.003507176	0.015147
**hsa-miR-6777-5p**	1.4344	0.00081	0.00313	1.0897	0.008089665	0.021136
**hsa-miR-6813-5p**	1.3152	0.00083	0.003176	1.1022	0.00397418	0.015595
**hsa-miR-502-3p**	1.3464	0.00085	0.003191	1.1085	0.005181537	0.017174
**hsa-miR-1292-5p**	1.3110	0.000859	0.003191	0.9838	0.008362225	0.021396
**hsa-let-7c-3p**	1.3957	0.000866	0.003191	1.1442	0.003464832	0.015147
**hsa-miR-374a-5p**	1.3610	0.000869	0.003191	1.1422	0.00467438	0.016562
**hsa-miR-6876-5p**	1.2722	0.00089	0.003236	1.0642	0.004156073	0.015654
**hsa-miR-486-5p**	-0.8078	0.000907	0.003268	-0.8250	0.000498743	0.010609
**hsa-miR-17-3p**	1.2728	0.000993	0.003459	1.1750	0.003287079	0.015147
**hsa-miR-3605-3p**	1.2894	0.00101	0.003459	1.0599	0.005705778	0.017174
**hsa-miR-485-5p**	1.1425	0.001012	0.003459	0.9316	0.007699513	0.020697
**hsa-miR-548am-5p**	1.2800	0.001012	0.003459	0.9509	0.007814353	0.020697
**hsa-miR-3659**	1.3105	0.001016	0.003459	1.0734	0.003572875	0.015147
**hsa-miR-1185-1-3p**	1.2568	0.001031	0.003478	1.1176	0.004413388	0.016374
**hsa-miR-148a-3p**	1.3059	0.001055	0.003527	1.0163	0.008566012	0.021767
**hsa-miR-199b-5p**	1.3268	0.001081	0.003573	1.1036	0.004133559	0.015654
**hsa-miR-3617-5p**	1.3317	0.001088	0.003573	1.0674	0.005704094	0.017174
**hsa-miR-1268a**	1.2983	0.001135	0.00363	1.0820	0.003378186	0.015147
**hsa-miR-31-5p**	1.9487	0.001147	0.003638	1.5763	0.004751788	0.016562
**hsa-miR-338-3p**	1.3124	0.001244	0.003879	1.0379	0.008359046	0.021396
**hsa-miR-3187-3p**	1.2514	0.001288	0.003983	0.9129	0.012086644	0.026691
**hsa-miR-671-5p**	1.3272	0.001319	0.00402	1.1325	0.005434341	0.017174
**hsa-miR-337-3p**	1.2656	0.001332	0.00402	1.0750	0.005611714	0.017174
**hsa-miR-1301-3p**	1.0510	0.001333	0.00402	0.7877	0.016048557	0.032934
**hsa-miR-6780a-5p**	1.2853	0.001394	0.004163	1.0702	0.006434942	0.018224
**hsa-miR-6512-3p**	1.3228	0.001407	0.004163	1.0542	0.009245956	0.022868
**hsa-miR-4433b-5p**	1.0474	0.001418	0.004163	0.9303	0.006979432	0.019469
**hsa-miR-550a-5p**	1.1056	0.001492	0.004259	0.9291	0.008767765	0.021979
**hsa-miR-550a-3-5p**	1.1056	0.001492	0.004259	0.9291	0.008767765	0.021979

**hsa-miR-106b-5p**	1.2213	0.001527	0.004312	0.9996	0.009342989	0.022872
**hsa-miR-345-5p**	1.3083	0.001534	0.004312	1.0749	0.007358171	0.019926
**hsa-miR-1270**	1.2101	0.00159	0.004436	0.9314	0.016983119	0.033875
**hsa-miR-6873-3p**	1.4321	0.001605	0.004444	1.1427	0.007866103	0.020697
**hsa-miR-548au-5p**	1.3331	0.001625	0.004464	1.0549	0.007195727	0.01963
**hsa-miR-30c-1-3p**	1.2577	0.001649	0.004474	1.1884	0.002814948	0.015147
**hsa-miR-641**	1.2026	0.001652	0.004474	0.9965	0.006188552	0.017798
**hsa-miR-766-3p**	1.1982	0.00171	0.004517	0.9571	0.01083907	0.024671
**hsa-miR-324-3p**	1.0962	0.001712	0.004517	0.8808	0.009746234	0.023179
**hsa-miR-146b-5p**	1.2457	0.001807	0.004721	1.1379	0.005591498	0.017174
**hsa-miR-584-5p**	1.2130	0.001843	0.004782	1.0489	0.003710726	0.015351
**hsa-miR-889-3p**	1.1911	0.001891	0.004873	1.2936	0.001879203	0.012911
**hsa-miR-101-3p**	1.2222	0.001926	0.004927	1.1337	0.00716721	0.01963
**hsa-miR-487b-3p**	1.1439	0.002024	0.005088	1.0761	0.005623254	0.017174
**hsa-miR-30b-3p**	1.1957	0.00203	0.005088	1.0005	0.009616524	0.023018
**hsa-miR-499a-5p**	1.1841	0.002083	0.005185	0.9034	0.010400993	0.024269
**hsa-miR-136-3p**	1.2352	0.002126	0.005257	1.1164	0.005655698	0.017174
**hsa-miR-3605-5p**	1.2196	0.002145	0.005271	1.0113	0.01049488	0.024335
**hsa-miR-134-5p**	1.1358	0.002207	0.005343	1.0654	0.007785971	0.020697
**hsa-miR-4488**	1.3013	0.002218	0.005343	1.1346	0.006162036	0.017798
**hsa-miR-4429**	1.2267	0.002271	0.005423	1.0411	0.007034558	0.019476
**hsa-miR-181c-3p**	1.2165	0.00228	0.005423	0.9860	0.008268814	0.021396
**hsa-miR-92b-3p**	1.3633	0.0023	0.005434	0.8831	0.04188919	0.067277
**hsa-miR-369-5p**	1.1458	0.002432	0.005711	1.0576	0.004771684	0.016562
**hsa-miR-95-3p**	1.4225	0.002534	0.005912	1.2193	0.012830543	0.027837
**hsa-miR-200c-3p**	1.3761	0.002607	0.006044	1.2000	0.010660341	0.024565
**hsa-miR-505-3p**	1.1153	0.002718	0.006262	0.9019	0.013449715	0.028677
**hsa-miR-150-3p**	1.2145	0.002802	0.006405	0.9922	0.013342398	0.028613
**hsa-miR-184**	2.0127	0.002814	0.006405	1.4845	0.021780949	0.040607
**hsa-miR-141-3p**	1.3526	0.002851	0.006449	1.3380	0.003993204	0.015595
**hsa-miR-505-5p**	0.9411	0.002913	0.006549	0.8321	0.010793013	0.024671
**hsa-miR-1304-3p**	1.0070	0.002945	0.006582	0.9595	0.009563143	0.023018
**hsa-miR-125a-5p**	0.9642	0.002974	0.006608	0.7612	0.032465429	0.055384
**hsa-miR-26b-3p**	1.1603	0.003031	0.006693	0.9598	0.013335857	0.028613
**hsa-miR-1299**	1.1435	0.003143	0.0069	0.9139	0.019192605	0.037086
**hsa-miR-337-5p**	1.2227	0.003298	0.007114	1.0877	0.009811292	0.023185
**hsa-miR-10399-5p**	1.1745	0.003369	0.007225	0.9571	0.01579103	0.032913
**hsa-miR-4758-5p**	2.0556	0.003391	0.00723	1.7124	0.011297338	0.025513
**hsa-miR-10401-3p**	1.1603	0.003415	0.00724	0.9221	0.018809249	0.036728
**hsa-miR-100-5p**	2.0861	0.003532	0.007445	1.5318	0.027389824	0.04862
**hsa-miR-32-3p**	1.2494	0.003631	0.00761	1.1993	0.005933883	0.017472
**hsa-miR-29c-5p**	1.0863	0.00383	0.007938	0.7827	0.019816312	0.037896
**hsa-miR-187-3p**	1.0837	0.003944	0.008129	0.8195	0.023430603	0.043033
**hsa-miR-4446-3p**	1.0443	0.004044	0.008253	0.8258	0.013768102	0.029188

**hsa-miR-18a-5p**	1.0761	0.004049	0.008253	0.8036	0.019144645	0.037086
**hsa-miR-181a-3p**	1.0911	0.004089	0.00829	0.8550	0.02451684	0.04437
**hsa-miR-6788-5p**	1.1071	0.004222	0.008514	0.8653	0.019654612	0.037782
**hsa-miR-323a-3p**	1.0272	0.004563	0.009151	0.9538	0.011346766	0.025513
**hsa-miR-548ax**	1.1382	0.004636	0.009211	1.0628	0.012223824	0.026835
**hsa-miR-642a-3p**	1.2392	0.004667	0.009211	0.9805	0.025092869	0.045192
**hsa-miR-671-3p**	1.0804	0.004768	0.009359	0.8482	0.017806238	0.03527
**hsa-miR-942-5p**	1.0743	0.004816	0.009392	0.8474	0.017872512	0.03527
**hsa-miR-421**	1.0012	0.004835	0.009392	0.9703	0.009399876	0.022872
**hsa-miR-6815-5p**	1.1885	0.005304	0.010249	0.8838	0.022091961	0.040891
**hsa-miR-133a-3p**	2.1880	0.005373	0.010329	1.6511	0.032850471	0.055398
**hsa-miR-431-5p**	0.9649	0.005412	0.01035	0.7758	0.018299275	0.035921
**hsa-miR-1273h-5p**	0.9666	0.005679	0.01075	0.8563	0.020972469	0.039297
**hsa-miR-145-5p**	1.1877	0.005679	0.01075	1.1450	0.011957401	0.026564
**hsa-miR-25-5p**	1.1046	0.006522	0.012221	#N/A	#N/A	#N/A
**hsa-miR-1283**	1.0985	0.006609	0.012322	0.8722	0.016150554	0.032934
**hsa-miR-194-5p**	1.1834	0.006727	0.012441	1.0346	0.016222987	0.032934
**hsa-miR-363-3p**	1.0890	0.007134	0.013054	0.8166	0.032543817	0.055384
**hsa-miR-3120-3p**	0.9403	0.007143	0.013054	0.8459	0.031889429	0.055028
**hsa-miR-133b**	1.5724	0.007299	0.013147	1.1729	0.038504818	0.06349
**hsa-miR-424-3p**	1.0249	0.007332	0.013147	#N/A	#N/A	#N/A
**hsa-miR-196a-5p**	1.2810	0.007335	0.013147	1.0499	0.037130024	0.061497
**hsa-miR-532-3p**	0.8999	0.007805	0.013922	#N/A	#N/A	#N/A
**hsa-miR-424-5p**	0.9168	0.008148	0.014464	1.0552	0.004776674	0.016562
**hsa-miR-17-5p**	0.9143	0.00821	0.014504	0.8017	0.029870575	0.052771
**hsa-miR-484**	1.0760	0.008327	0.014641	1.0301	0.020501913	0.03861
**hsa-miR-191-3p**	0.9168	0.009128	0.015973	0.7952	0.035047941	0.058571
**hsa-miR-28-5p**	0.9633	0.009365	0.016311	0.8134	0.032727731	0.055398
**hsa-miR-501-3p**	1.0798	0.009582	0.016612	0.7610	0.049944098	0.078514
**hsa-miR-4508**	0.9822	0.009897	0.017078	#N/A	#N/A	#N/A
**hsa-miR-7-5p**	1.0781	0.010135	0.017408	0.8896	0.030770556	0.053345
**hsa-miR-497-5p**	0.8909	0.010614	0.017981	0.9204	0.016462624	0.033194
**hsa-miR-4286**	1.0010	0.011706	0.019741	0.9077	0.030606805	0.053345
**hsa-miR-20a-5p**	1.0203	0.011989	0.020127	0.8247	0.044665332	0.071426
**hsa-miR-186-5p**	1.2222	0.012063	0.02016	1.1979	0.020204054	0.03844
**hsa-miR-181a-2-3p**	1.0523	0.013692	0.022677	0.8967	0.039011295	0.063759
**hsa-miR-324-5p**	0.8525	0.013974	0.023041	0.9428	0.014057768	0.029633
**hsa-miR-6891-5p**	1.0429	0.014348	0.023554	0.9023	0.040407844	0.065464
**hsa-miR-486-3p**	0.9449	0.015293	0.024995	#N/A	#N/A	#N/A
**hsa-miR-205-5p**	1.6291	0.016029	0.025969	1.8011	0.009979222	0.023432
**hsa-miR-11400**	0.8552	0.016614	0.026799	0.8303	0.030640586	0.053345
**hsa-miR-183-5p**	1.0056	0.017145	0.027485	#N/A	#N/A	#N/A
**hsa-miR-181b-5p**	0.8649	0.017187	0.027485	#N/A	#N/A	#N/A
**hsa-miR-629-5p**	-0.7689	0.017377	0.02767	-0.6775	0.045406749	0.071991

**hsa-miR-151b**	-0.7104	0.020861	0.033074	-0.6752	0.026070342	0.046725
**hsa-miR-15b-3p**	0.9239	0.021291	0.033612	#N/A	#N/A	#N/A
**hsa-miR-23b-3p**	1.4725	0.021595	0.033805	1.5037	0.026238075	0.0468
**hsa-miR-941**	0.9458	0.022444	0.034986	#N/A	#N/A	#N/A
**hsa-miR-3143**	0.8789	0.023213	0.036033	#N/A	#N/A	#N/A
**hsa-miR-203a-3p**	1.1739	0.023898	0.036942	#N/A	#N/A	#N/A
**hsa-miR-375-3p**	1.1379	0.02453	0.037761	#N/A	#N/A	#N/A
**hsa-miR-323b-3p**	0.8958	0.025985	0.039837	0.9098	0.033663802	0.056513
**hsa-miR-409-3p**	0.8695	0.026349	0.040228	1.0638	0.009432497	0.022872
**hsa-miR-1180-3p**	0.8530	0.026544	0.04036	#N/A	#N/A	#N/A
**hsa-miR-4448**	2.0454	0.028891	0.043571	#N/A	#N/A	#N/A
**hsa-miR-8485**	1.6642	0.029852	0.044763	#N/A	#N/A	#N/A
**hsa-miR-598-3p**	0.7800	0.029922	0.044763	0.8143	0.040084014	0.065224
**hsa-miR-3138**	0.8064	0.033966	0.050607	#N/A	#N/A	#N/A
**hsa-miR-139-3p**	0.7905	0.034426	0.051088	#N/A	#N/A	#N/A
**hsa-miR-140-3p**	0.8486	0.035148	0.051846	#N/A	#N/A	#N/A
**hsa-miR-589-5p**	0.7547	0.035216	0.051846	#N/A	#N/A	#N/A
**hsa-miR-151a-5p**	-0.6107	0.038042	0.055785	#N/A	#N/A	#N/A
**hsa-miR-28-3p**	-0.8159	0.039886	0.058259	#N/A	#N/A	#N/A
**hsa-miR-652-3p**	0.7612	0.041224	0.059977	#N/A	#N/A	#N/A
**hsa-miR-4516**	0.8479	0.046499	0.067156	#N/A	#N/A	#N/A
**hsa-miR-4433b-3p**	0.7211	0.046521	0.067156	0.9350	0.023791002	0.043267

Table S10: CI vs NC. DE miRNAs unique to MAs: DE miRNAs common to QIAGEN RNA-seq portal and Dream analysis with and without controlling for the covariables

miRNA	QIAGEN		DREAM		DREAM controlled for CVs	
	Fold change	P-value	logFC	P.Value	logFC	P.Value
**hsa-miR-26a-5p**	2.935283	8.72E-10	1.349862	0.000177	1.395504	0.0009
**hsa-let-7f-5p**	1.926349	1.41E-06	0.658384	0.006401	0.635749	0.023644
**hsa-let-7e-5p**	1.998463	2.62E-05	1.142666	0.000101	1.105747	0.000787
**hsa-miR-98-5p**	2.216536	3.85E-05	0.891332	0.04593	#N/A	#N/A
**hsa-miR-139-5p**	2.315688	0.000145	1.367835	0.001425	1.11284	0.018717
**hsa-miR-155-5p**	2.805184	0.000171	1.020638	0.030071	#N/A	#N/A
**hsa-miR-26b-5p**	1.915892	0.000174	0.931972	0.006605	0.81978	0.031822
**hsa-miR-15a-5p**	-1.68893	0.000699	-0.79289	0.017057	#N/A	#N/A
**hsa-miR-25-3p**	-1.61422	0.001489	-1.05329	0.005968	#N/A	#N/A
**hsa-let-7b-5p**	1.451954	0.002433	0.492351	0.026762	#N/A	#N/A
**hsa-miR-30e-3p**	1.59172	0.004804	0.739263	0.027962	#N/A	#N/A
**hsa-miR-744-5p**	1.855302	0.005264	0.910639	0.026375	0.960467	0.042363
**hsa-miR-144-3p**	-1.57101	0.01791	-0.9948	0.038112	#N/A	#N/A

Table S11: CI vs NC. miRNAs only significant to NHWs: DE miRNAs common to QIAGEN RNA-seq portal and Dream analysis with and without controlling for the covariables.

miRNA	QIAGEN		DREAM		DREAM controlled for CVs	
	Fold change	P-value	logFC	P.Value	logFC	P.Value
**hsa-miR-184**	9.2766	3.14E-12	2.0127	0.002814	1.5318	0.02739
**hsa-miR-7-5p**	4.4521	2.87E-09	1.0781	0.010135	1.5763	0.004752
**hsa-miR-125b-5p**	4.5835	4.7E-07	2.2100	0.000582	-0.6775	0.045407
**hsa-miR-133a-3p**	4.7488	6.13E-07	2.1880	0.005373	#N/A	#N/A
**hsa-miR-100-5p**	4.1458	3.26E-06	2.0861	0.003532	#N/A	#N/A
**hsa-miR-31-5p**	4.8501	1.29E-05	1.9487	0.001147	#N/A	#N/A
**hsa-miR-486-5p**	-1.7962	4.66E-05	-0.8078	0.000907	#N/A	#N/A
**hsa-miR-629-5p**	-1.6041	0.00293	-0.7689	0.017377	#N/A	#N/A
**hsa-miR-589-5p**	-2.1599	0.007879	0.7547	0.035216	#N/A	#N/A
**hsa-miR-1180-3p**	-2.4419	0.013726	0.8530	0.026544	#N/A	#N/A

Visit 2 vs Visit 1: Differentially expressed miRNAs between Visit 2 (V2) and Visit 1 (V1) for both Mexican Americans (MA) and Non-Hispanic Whites (NHW)

Analyses were done both in QIAGEN RNA-seq portal (QIAGEN) and DEseq2 in R programming

2. DE miRNAs common to QIAGEN RNA-seq portal, DEseq in R with and without controlling for Covariables

QIAGEN RNA-seq portal: Threshold of False Discovery Rate (FDR) p-value threshold of <=0.1 and the fold change greater than 1.1 or less than -1.1

DEseq2: Threshold of miRNAs with mean count < 10 across samples and the raw p- value <0.05

CV: Covariables including Age, presence of APOE4 allele (APOE4_Index), presence of Metabolic comorbidities (Metabolic Index), Gender (Male/Female)

Table S12: MA: CI group V2 vs V1

miRNA	QIAGEN		DEseq		DEseq controlled for CV	
	Fold Change	P-value	log2Fold Change	P-value	log2Fold Change	P-value
**hsa-miR-499a-5p**	21.1916	0.00003385	N/A	N/A	N/A	N/A
**hsa-miR-184**	N/A	N/A	-2.2055	0.000951269	-1.8236	0.0491992

**hsa-miR-324-5p**	N/A	N/A	1.8707	0.001643094	1.7638	0.0030071
**hsa-miR-26a-5p**	N/A	N/A	0.9759	0.002362528	0.9931	0.0026158
**hsa-miR-1268b**	N/A	N/A	-2.7423	0.007950215	-2.5602	0.0205507
**hsa-miR-1307-3p**	N/A	N/A	1.2662	0.01341954	1.5379	0.0035893
**hsa-miR-98-5p**	N/A	N/A	0.9709	0.013888829	0.9490	0.0208642
**hsa-miR-326**	N/A	N/A	2.2103	0.017594625	2.3671	0.017768
**hsa-miR-744-5p**	N/A	N/A	1.1047	0.024409022	1.1052	0.0286994
**hsa-miR-12136**	N/A	N/A	2.8788	0.032261188	N/A	N/A
**hsa-miR-133b**	N/A	N/A	-3.1213	0.032410029	N/A	N/A
**hsa-miR-26b-5p**	N/A	N/A	0.6761	0.03856742	N/A	N/A
**hsa-miR-4787-5p**	N/A	N/A	-2.4008	0.041791317	N/A	N/A
**hsa-miR-186-5p**	N/A	N/A	1.8926	0.044134346	N/A	N/A
**hsa-miR-142-3p**	N/A	N/A	0.9846	0.045404186	1.0186	0.0463333
**hsa-miR-203a-3p**	N/A	N/A	N/A	N/A	-2.4884	0.0085673
**hsa-miR-4732-5p**	N/A	N/A	N/A	N/A	-1.3998	0.0187912
**hsa-miR-425-5p**	N/A	N/A	N/A	N/A	1.2121	0.0300818
**hsa-miR-191-5p**	N/A	N/A	N/A	N/A	0.4630	0.040304
**hsa-miR-4758-5p**	N/A	N/A	N/A	N/A	-1.1168	0.0458644
**hsa-miR-1-3p**	N/A	N/A	N/A	N/A	1.2349	0.0497056

Table S13: NHW: CI group V2 vs V1

**miRNA**	**QIAGEN**		**DEseq**		**DEseq controlled for CV**	
	Fold Change	P-value	log2Fold Change	P-value	log2Fold Change	P-value
**hsa-miR-1-3p**	-11.8004	7.6E-09	N/A	N/A	N/A	N/A
**hsa-miR-7-5p**	-4.3256	0.00011	N/A	N/A	-1.1518	0.04997
**hsa-miR-184**	-5.2406	0.0003	N/A	N/A	N/A	N/A
**hsa-miR-3182**	-5.7600	0.00118	N/A	N/A	N/A	N/A
**hsa-miR-877-5p**	N/A	N/A	1.4199	0.011227479	1.7341	0.00198
**hsa-miR-1908-5p**	N/A	N/A	1.5234	0.025475819	1.3359	0.03834
**hsa-miR-483-5p**	N/A	N/A	1.4525	0.027634056	1.2872	0.04603
**hsa-miR-221-3p**	N/A	N/A	N/A	N/A	1.7292	0.02078
**hsa-miR-23a-3p**	N/A	N/A	N/A	N/A	0.8657	0.03047

Table S14: MA: NC group V2 vs V1

**miRNA**	**QIAGEN**		**DEseq**		**DEseq controlled for CV**	
	Fold Change	P-value	log2Fold Change	P-value	log2Fold Change	P-value

**hsa-miR-335-5p**	-1.9741	0.004743	-1.1192	0.00316681	-1.1116	0.00262096
**hsa-miR-5010-5p**	N/A	N/A	-2.1865	0.017184524	-2.3015	0.0194329
**hsa-miR-382-5p**	N/A	N/A	-1.3916	0.017610319	-1.3148	0.02789662
**hsa-miR-17-5p**	N/A	N/A	-1.7403	0.021818753	-1.5963	0.0418573
**hsa-miR-196a-5p**	N/A	N/A	2.2901	0.029995055	2.2699	0.0308393
**hsa-miR-323b-3p**	N/A	N/A	-3.2190	0.041314128	N/A	N/A
**hsa-miR-154-5p**	N/A	N/A	N/A	N/A	-2.2499	0.0203025
**hsa-miR-155-5p**	N/A	N/A	N/A	N/A	1.4541	0.0235081
**hsa-miR-224-5p**	N/A	N/A	N/A	N/A	-2.3347	0.0490161

Table S15: NHW: NC group V2 vs V1

**miRNA**	**QIAGEN**		**DEseq**		**DEseq controlled for CV**	
	Fold Change	P-value	log2Fold Change	P-value	log2Fold Change	P-value
hsa-miR-1307-3p	4.2250	2.8E-05	2.0452	0.005773088	2.1342	0.006283
hsa-miR-34c-5p	-26.1719	9.5E-05	N/A	N/A	N/A	N/A
hsa-miR-451a	-3.4438	0.00018	-1.5720	0.005450206	-1.6012	0.003815
hsa-miR-486-5p	-2.0461	0.00025	-0.9926	0.000301182	-1.0316	0.000185
hsa-miR-1908-5p	3.0481	0.00035	1.6079	0.002631179	1.5007	0.004497
hsa-miR-432-5p	2.9506	0.00069	1.6207	0.004979602	1.2323	0.034856
hsa-miR-191-3p	5.3424	0.00097	N/A	N/A	N/A	N/A
hsa-miR-1-3p	-3.7294	0.00097	N/A	N/A	N/A	N/A
hsa-miR-5187-5p	6.6980	0.00223	N/A	N/A	N/A	N/A
hsa-miR-1273h-5p	4.8207	0.00324	N/A	N/A	N/A	N/A
hsa-miR-151a-3p	2.0271	0.00342	1.1500	0.016765645	N/A	N/A
hsa-miR-4732-5p	-2.4793	0.00386	-1.5042	0.006420143	-1.3562	0.016438
hsa-miR-4433b-5p	3.1824	0.00438	N/A	N/A	N/A	N/A
hsa-miR-101-3p	-2.9071	0.00471	-2.2466	0.032557172	N/A	N/A
hsa-miR-339-3p	2.1754	0.00497	N/A	N/A	N/A	N/A
hsa-miR-1306-5p	3.3914	0.00617	N/A	N/A	N/A	N/A
hsa-miR-31-5p	-4.0994	0.00669	N/A	N/A	N/A	N/A
hsa-miR-425-3p	2.0149	0.0077	N/A	N/A	N/A	N/A
hsa-miR-760	3.7192	0.00927	2.8304	0.045874454	N/A	N/A
hsa-miR-95-3p	-3.2765	0.00931	-2.6539	0.024764551	-2.8986	0.023124
hsa-miR-3120-3p	3.1677	0.00962	N/A	N/A	N/A	N/A
hsa-miR-574-3p	2.2538	0.00999	N/A	N/A	N/A	N/A
hsa-miR-1304-3p	2.7015	0.01014	N/A	N/A	N/A	N/A
hsa-let-7d-3p	-2.0833	0.01057	N/A	N/A	N/A	N/A
hsa-miR-107	N/A	N/A	-0.8668	0.004312495	-0.9597	0.000202
hsa-miR-30d-5p	N/A	N/A	0.6843	0.008092844	0.6606	0.013664

hsa-miR-103a-3p	N/A	N/A	-0.6819	0.011469409	-0.7729	0.000502
hsa-miR-122-5p	N/A	N/A	-1.0861	0.012057693	N/A	N/A
hsa-miR-361-5p	N/A	N/A	1.2638	0.014373789	N/A	N/A
hsa-miR-151a-5p	N/A	N/A	0.5455	0.029615406	0.4878	0.043807
hsa-miR-142-5p	N/A	N/A	-0.6367	0.033548042	N/A	N/A
hsa-miR-6734-5p	N/A	N/A	1.5704	0.044032081	N/A	N/A
hsa-miR-424-3p	N/A	N/A	-2.7463	0.047247558	N/A	N/A
hsa-miR-126-5p	N/A	N/A	N/A	N/A	0.6403	0.027116
hsa-miR-93-5p	N/A	N/A	N/A	N/A	0.5236	0.046815

Table S16: Unique DE miRNAs identified in Visit 2 in comparison to Visit 1 in the CI group after controlling for the covariables in DEseq

**MA**			**NHW**		
miRNA	log2Fold Change	P-value	miRNA	log2Fold Change	P-value
Upregulated			Upregulated		
**hsa-miR-425-5p**	1.212143826	0.030082	**hsa-miR-221-3p**	1.729203	0.020777
**hsa-miR-191-5p**	0.462981352	0.040304	**hsa-miR-23a-3p**	0.865721	0.030467
**hsa-miR-1-3p**	1.234867081	0.049706			
			Downregulated		
Downregulated			**hsa-miR-7-5p**	-1.1518	0.049965
**hsa-miR-203a-3p**	-2.488427506	0.008567			
**hsa-miR-4732-5p**	-1.399764946	0.018791			
**hsa-miR-4758-5p**	-1.116849032	0.045864			
