# Supplementary material for: Neuronal Enriched Extracellular Vesicle miR-122-5p as a Potential Biomarker for Alzheimer’s Disease
Source: Cells. 2025 Nov 13;14(22):1784. doi: 10.3390/cells14221784 (PMC12651308; doi:10.3390/cells14221784)
Supplement: Supplementary file 1 [file cells-14-01784-s001.zip › Supplementary Document S3.pdf]

Supplementary Document S3

Mexican Americans

Model Statistics from DREAM after accounting for the repeated measures due to longitudinal data points

1. Results for hsa-miR-122-5p

Covariate	Beta	SE	t	p	Signif
(Intercept)	25.03005868	2.0545837	12.1825451	1.017858e-16	***
GroupCI	-1.60561484	0.4579977	-3.5057271	9.588277e-04	***
SexMale	0.90731256	0.4420196	2.0526524	4.525514e-02	*
Visit_GroupV2	-0.05299682	0.2242932	-0.2362836	8.141591e-01	
AgeAtVisit	-0.12868489	0.0302039	-4.2605397	8.788010e-05	***
APOE4_INDEX	0.54431620	0.3947189	1.3789972	1.739146e-01	
Metabolic_Index	-0.12917118	0.1915553	-0.6743284	5.031483e-01	

DE miRNAs in CI vs NC unique to Mexican Americans

2. Results for hsa-let-7e-5p

Covariate	Beta	SE	t	p	Signif
(Intercept)	10.947957989	1.37055933	7.987948943	3.081337e-12	***
GroupCI	1.105746875	0.31882389	3.468205847	7.871396e-04	***
SexMale	-0.001774596	0.30660256	-0.005787936	9.953940e-01	
Visit_GroupV2	0.334677658	0.28174700	1.187865905	2.378288e-01	
AgeAtVisit	-0.022337630	0.02007697	-1.112599391	2.686702e-01	
APOE4_INDEX	0.064987253	0.26232728	0.247733494	8.048719e-01	
Metabolic_Index	-0.108673849	0.13020355	-0.834645835	4.059978e-01	

-----

3. Results for hsa-miR-26a-5p

Covariate	Beta	SE	t	p	Signif
(Intercept)	11.059594874	1.72318976	6.41809458	5.813697e-08	***
GroupCI	1.395504336	0.39642184	3.52025090	9.576419e-04	***
SexMale	0.016094422	0.38424456	0.04188588	9.667639e-01	
Visit_GroupV2	0.578263906	0.24946554	2.31801119	2.476984e-02	*
AgeAtVisit	-0.006774917	0.02528021	-0.26799291	7.898566e-01	
APOE4_INDEX	0.029477824	0.33486213	0.08802973	9.302204e-01	
Metabolic_Index	0.136331766	0.16234825	0.83974891	4.052250e-01	

-----

4. Results for hsa-miR-139-5p

Covariate	Beta	SE	t	p	Signif
(Intercept)	8.58034196	2.05838306	4.1684865	0.0001207188	***
GroupCI	1.11284017	0.45786391	2.4305043	0.0186858501	*
SexMale	0.70802097	0.44254519	1.5998840	0.1158878790	
Visit_GroupV2	0.24640897	0.27557895	0.8941502	0.3755004515	
AgeAtVisit	-0.02032214	0.03014153	-0.6742237	0.5032562909	
APOE4_INDEX	0.43353110	0.39363223	1.1013608	0.2759825469	
Metabolic_Index	0.06190596	0.18899826	0.3275478	0.7446131353	

-----

5. Results for hsa-miR-15b-5p

Covariate	Beta	SE	t	p	Signif
(Intercept)	13.01142931	0.96203009	13.5249712	1.523848e-18	***
GroupCI	-0.40113171	0.21816051	-1.8386999	7.175361e-02	.
SexMale	0.50819142	0.21239364	2.3926866	2.042568e-02	*
Visit_GroupV2	0.10281018	0.17002430	0.6046793	5.480585e-01	
AgeAtVisit	-0.01172477	0.01412493	-0.8300762	4.103435e-01	
APOE4_INDEX	-0.62637664	0.19281527	-3.2485843	2.049190e-03	**
Metabolic_Index	0.15935061	0.08931807	1.7840803	8.032790e-02	.

-----

6. Results for hsa-miR-3688-3p

Covariate	Beta	SE	t	p	Signif
(Intercept)	5.660572821	1.01050918	5.60170350	2.036511e-07	***
GroupCI	-0.723510771	0.22456743	-3.22179737	1.742005e-03	**
SexMale	-0.095352064	0.21568200	-0.44209561	6.594184e-01	
Visit_GroupV2	-0.006911888	0.20498943	-0.03371826	9.731721e-01	
AgeAtVisit	-0.006606188	0.01464731	-0.45101727	6.529984e-01	
APOE4_INDEX	0.378247518	0.20093364	1.88244993	6.281586e-02	.
Metabolic_Index	0.045744333	0.09289446	0.49243336	6.235419e-01	

-----

7. Results for hsa-miR-132-3p

Covariate	Beta	SE	t	p	Signif
(Intercept)	6.58605394	1.11049291	5.9307483	4.791537e-08	***
GroupCI	-0.77509508	0.24448066	-3.1703738	2.045970e-03	**
SexMale	0.10439223	0.23680396	0.4408382	6.603253e-01	
Visit_GroupV2	-0.05937712	0.22326189	-0.2659528	7.908482e-01	
AgeAtVisit	-0.01896214	0.01608473	-1.1788906	2.413673e-01	
APOE4_INDEX	0.37874679	0.21826890	1.7352302	8.592220e-02	.
Metabolic_Index	0.04543735	0.10090980	0.4502768	6.535302e-01	

-----

8. Results for hsa-miR-25-3p

Covariate	Beta	SE	t	p	Signif
(Intercept)	11.5242758	1.60531279	7.178835	9.014514e-09	***
GroupCI	-0.6326347	0.36174108	-1.748860	8.776777e-02	.
SexMale	0.8551193	0.35176598	2.430932	1.950010e-02	*
Visit_GroupV2	0.2550049	0.26887535	0.948413	3.484531e-01	
AgeAtVisit	-0.0275758	0.02359582	-1.168673	2.492577e-01	
APOE4_INDEX	-0.8313783	0.31929393	-2.603803	1.276506e-02	*
Metabolic_Index	0.2527086	0.14873103	1.699098	9.684592e-02	.

-----

9. Results for hsa-let-7a-5p

Covariate	Beta	SE	t	p	Signif
(Intercept)	16.09551842	1.25399495	12.8353933	1.382892e-17	***
GroupCI	0.63206254	0.28397996	2.2257294	3.048623e-02	*
SexMale	0.25763630	0.27452817	0.9384694	3.524331e-01	
Visit_GroupV2	0.20899083	0.15819377	1.3211066	1.923714e-01	
AgeAtVisit	-0.01138049	0.01839759	-0.6185857	5.389465e-01	
APOE4_INDEX	0.09483487	0.24326326	0.3898446	6.982772e-01	
Metabolic_Index	-0.01707664	0.11697356	-0.1459872	8.845079e-01	

-----

10. Results for hsa-let-7f-5p

Covariate	Beta	SE	t	p	Signif
(Intercept)	14.599043301	1.19334363	12.2337297	7.902983e-17	***
GroupCI	0.635749120	0.27315251	2.3274512	2.392493e-02	*
SexMale	0.224039623	0.26454030	0.8469017	4.009849e-01	
Visit_GroupV2	0.295226969	0.17284926	1.7080025	9.368323e-02	.
AgeAtVisit	-0.002476106	0.01751016	-0.1414097	8.881003e-01	
APOE4_INDEX	0.037836541	0.23231321	0.1628687	8.712619e-01	
Metabolic_Index	0.076991041	0.11194841	0.6877368	4.947175e-01	

-----

11. Results for hsa-miR-26b-5p

Covariate	Beta	SE	t	p	Signif
(Intercept)	8.57370075	1.61004895	5.3251181	2.716828e-06	***
GroupCI	0.81977996	0.37164822	2.2057955	3.227643e-02	*
SexMale	0.05569045	0.35925725	0.1550155	8.774666e-01	
Visit_GroupV2	0.45503955	0.29010978	1.5685081	1.234083e-01	
AgeAtVisit	0.03294228	0.02349543	1.4020721	1.674023e-01	
APOE4_INDEX	0.10404804	0.31128991	0.3342480	7.396654e-01	
Metabolic_Index	0.17062086	0.14972035	1.1395970	2.601747e-01	

-----

12. Results for hsa-miR-4669

Covariate	Beta	SE	t	p	Signif
(Intercept)	5.323383803	1.14156717	4.66322434	2.885418e-05	***
GroupCI	-0.612700700	0.25595726	-2.39376176	2.098675e-02	*
SexMale	-0.287434362	0.24488441	-1.17375527	2.467802e-01	

Covariate	Beta	SE	t	p	Signif
Visit_GroupV2	-0.153554580	0.21128147	-0.72677730	4.711949e-01	
AgeAtVisit	0.002781048	0.01669155	0.16661409	8.684337e-01	
APOE4_INDEX	0.052531653	0.22810845	0.23029245	8.189273e-01	
Metabolic_Index	0.008392483	0.10682925	0.07855979	9.377374e-01	

-----

13. Results for hsa-miR-1260b

Covariate	Beta	SE	t	p	Signif
(Intercept)	8.889184862	1.75813110	5.05604210	2.059642e-06	***
GroupCI	1.217256690	0.39733064	3.06358623	2.841350e-03	**
SexMale	-0.655440514	0.38780079	-1.69014744	9.425787e-02	.
Visit_GroupV2	0.482838910	0.35782880	1.34935731	1.804090e-01	
AgeAtVisit	-0.001300295	0.02563972	-0.05071408	9.596592e-01	
APOE4_INDEX	-0.442270470	0.33158053	-1.33382522	1.854299e-01	
Metabolic_Index	-0.098921569	0.16300981	-0.60684428	5.453927e-01	

-----

14. Results for hsa-miR-154-5p

Covariate	Beta	SE	t	p	Signif
(Intercept)	6.935766458	1.71701310	4.0394371	0.0001082143	***
GroupCI	-0.817693508	0.35933798	-2.2755555	0.0251053110	*
SexMale	0.384772853	0.36015560	1.0683517	0.2880519400	
Visit_GroupV2	-0.364871640	0.33475214	-1.0899755	0.2784634803	
AgeAtVisit	-0.007028219	0.02504473	-0.2806266	0.7796031418	
APOE4_INDEX	0.050427453	0.31314523	0.1610354	0.8724053409	
Metabolic_Index	0.047180041	0.15000685	0.3145192	0.7538120269	

-----

15. Results for hsa-let-7g-5p

Covariate	Beta	SE	t	p	Signif
(Intercept)	16.51483736	1.09091752	15.1384839	5.074070e-20	***
GroupCI	0.55140573	0.25040955	2.2020156	3.244385e-02	*
SexMale	0.18270596	0.24281699	0.7524431	4.554158e-01	
Visit_GroupV2	0.17018453	0.15588724	1.0917156	2.803438e-01	
AgeAtVisit	-0.02110744	0.01600696	-1.3186413	1.934729e-01	
APOE4_INDEX	0.14052611	0.21334597	0.6586771	5.132135e-01	

Covariate	Beta	SE	t	p	Signif
Metabolic_Index	0.06506642	0.10290544	0.6322933	5.301605e-01	

-----

16. Results for hsa-miR-15a-5p

Covariate	Beta	SE	t	p	Signif
(Intercept)	9.51745474	1.38856846	6.8541487	6.996157e-10	***
GroupCI	-0.47981063	0.31682221	-1.5144476	1.332114e-01	
SexMale	0.76957041	0.30843841	2.4950537	1.430815e-02	*
Visit_GroupV2	-0.16206010	0.28227579	-0.5741197	5.672351e-01	
AgeAtVisit	0.01630001	0.02037564	0.7999753	4.257083e-01	
APOE4_INDEX	-0.67195334	0.28432425	-2.3633346	2.013544e-02	*
Metabolic_Index	0.33979578	0.12890577	2.6360013	9.789373e-03	**

-----

17. Results for hsa-miR-379-5p

Covariate	Beta	SE	t	p	Signif
(Intercept)	4.59001068	1.17162746	3.9176366	0.0003166547	***
GroupCI	-0.60407079	0.25931304	-2.3295041	0.0246164194	*
SexMale	-0.05280414	0.25067272	-0.2106497	0.8341595334	
Visit_GroupV2	-0.25695154	0.20824801	-1.2338727	0.2239778940	
AgeAtVisit	0.01081332	0.01706124	0.6337945	0.5295851063	
APOE4_INDEX	0.13149643	0.23068779	0.5700190	0.5716427040	
Metabolic_Index	0.07056965	0.10819174	0.6522647	0.5177156916	

-----

18. Results for hsa-miR-92b-5p

Covariate	Beta	SE	t	p	Signif
(Intercept)	6.05700130	1.26784100	4.77741397	6.405970e-06	***
GroupCI	-0.80786077	0.28053006	-2.87976547	4.911554e-03	**
SexMale	-0.01831009	0.27232601	-0.06723592	9.465343e-01	
Visit_GroupV2	0.14773391	0.25719118	0.57441280	5.670376e-01	
AgeAtVisit	-0.01159729	0.01837115	-0.63127712	5.293657e-01	
APOE4_INDEX	0.49607829	0.25056665	1.97982569	5.059827e-02	.
Metabolic_Index	0.05959687	0.11628734	0.51249668	6.094854e-01	

-----

19. Results for hsa-miR-4747-5p

Covariate	Beta	SE	t	p	Signif
(Intercept)	6.16817792	1.19720977	5.1521280	1.382010e-06	***
GroupCI	-0.54985295	0.26465294	-2.0776378	4.042321e-02	*
SexMale	0.05107526	0.25722434	0.1985631	8.430261e-01	
Visit_GroupV2	-0.05583585	0.24124949	-0.2314444	8.174642e-01	
AgeAtVisit	-0.01021490	0.01744293	-0.5856187	5.595116e-01	
APOE4_INDEX	0.02851552	0.23583860	0.1209112	9.040151e-01	
Metabolic_Index	-0.06110895	0.11012148	-0.5549231	5.802432e-01	

-----

20. Results for hsa-miR-92a-1-5p

Covariate	Beta	SE	t	p	Signif
(Intercept)	5.2191095559	1.14432358	4.56086865	3.765149e-05	***
GroupCI	-0.6533370191	0.25233576	-2.58915749	1.283412e-02	*
SexMale	-0.0248899897	0.24357965	-0.10218419	9.190540e-01	
Visit_GroupV2	-0.1340598638	0.22695364	-0.59069274	5.576136e-01	
AgeAtVisit	-0.0003171791	0.01658333	-0.01912639	9.848229e-01	
APOE4_INDEX	0.3163679635	0.22508414	1.40555423	1.665658e-01	
Metabolic_Index	0.1081482019	0.10448221	1.03508725	3.060319e-01	

-----

21. Results for hsa-miR-744-5p

Covariate	Beta	SE	t	p	Signif
(Intercept)	7.078024949	2.11079280	3.35325426	0.001510212	**
GroupCI	0.960466560	0.46027057	2.08674336	0.041917992	*
SexMale	-0.252713543	0.44841812	-0.56356675	0.575513370	
Visit_GroupV2	0.479227790	0.40679455	1.17805852	0.244229218	
AgeAtVisit	0.007046614	0.03085009	0.22841472	0.820235246	
APOE4_INDEX	-0.089920248	0.39354150	-0.22848987	0.820177142	
Metabolic_Index	-0.012450901	0.19121683	-0.06511404	0.948337212	

-----

22. Results for hsa-miR-96-5p

Covariate	Beta	SE	t	p	Signif
(Intercept)	5.2024350489	1.12333060	4.63125907	3.684824e-05	***
GroupCI	-0.6173244993	0.25171607	-2.45246358	1.856383e-02	*
SexMale	-0.0504943572	0.24290028	-0.20788102	8.363581e-01	
Visit_GroupV2	-0.0499125125	0.21363908	-0.23363007	8.164427e-01	
AgeAtVisit	0.0006210915	0.01634581	0.03799698	9.698758e-01	
APOE4_INDEX	0.2406996503	0.22432528	1.07299388	2.895915e-01	
Metabolic_Index	0.0043516188	0.10505852	0.04142090	9.671628e-01	

-----

23. Results for hsa-let-7b-5p

Covariate	Beta	SE	t	p	Signif
(Intercept)	16.607564694	1.06457311	15.60021052	1.837114e-20	***
GroupCI	0.426033073	0.24240646	1.75751533	8.518429e-02	.
SexMale	0.343944620	0.23442346	1.46719371	1.488230e-01	
Visit_GroupV2	0.113862488	0.17826867	0.63871286	5.260354e-01	
AgeAtVisit	-0.012613295	0.01559507	-0.80880006	4.226073e-01	
APOE4_INDEX	0.112752241	0.20638227	0.54632716	5.873630e-01	
Metabolic_Index	0.007540318	0.09930186	0.07593331	9.397867e-01	

-----

24. Results for hsa-miR-1260a

Covariate	Beta	SE	t	p	Signif
(Intercept)	9.54558059	2.28924944	4.1697425	0.0001126203	***
GroupCI	1.21812357	0.50544427	2.4100057	0.0194322518	*
SexMale	-0.56675109	0.49582895	-1.1430375	0.2581268100	
Visit_GroupV2	0.57978524	0.40140217	1.4443999	0.1544745099	
AgeAtVisit	-0.01677264	0.03345698	-0.5013197	0.6182086808	
APOE4_INDEX	-0.44548698	0.43096901	-1.0336868	0.3059459798	
Metabolic_Index	-0.04768577	0.21095583	-0.2260462	0.8220293124	

-----

25. Results for hsa-miR-30e-3p

Covariate	Beta	SE	t	p	Signif
(Intercept)	4.72565809	1.56702559	3.01568661	0.004173197	**
GroupCI	0.49815266	0.35599745	1.39931524	0.168455272	
SexMale	-0.12822144	0.34268158	-0.37417079	0.710004071	
Visit_GroupV2	0.51745760	0.27423912	1.88688470	0.065524135	.
AgeAtVisit	0.06243679	0.02287562	2.72940362	0.008972106	**
APOE4_INDEX	0.22799966	0.29857738	0.76362001	0.449009814	
Metabolic_Index	0.01344844	0.14362636	0.09363492	0.925807607	

-----

26. Results for hsa-miR-155-5p

Covariate	Beta	SE	t	p	Signif
(Intercept)	7.76944231	2.33094842	3.3331678	0.001583355	**
GroupCI	0.75693938	0.51502719	1.4697076	0.147637301	
SexMale	0.47115166	0.49948110	0.9432823	0.349878130	
Visit_GroupV2	0.58413096	0.33395474	1.7491321	0.086140741	.
AgeAtVisit	-0.01966397	0.03411055	-0.5764776	0.566767754	
APOE4_INDEX	0.61713126	0.44247154	1.3947366	0.168997120	
Metabolic_Index	0.21105708	0.21219069	0.9946576	0.324487227	

-----

27. Results for hsa-miR-766-5p

Covariate	Beta	SE	t	p	Signif
(Intercept)	4.916111474	1.04231284	4.7165412	4.021799e-05	***
GroupCI	-0.771033302	0.23221019	-3.3204111	2.160180e-03	**
SexMale	-0.077749788	0.22710118	-0.3423575	7.341971e-01	

Covariate	Beta	SE	t	p	Signif
Visit_GroupV2	-0.461616132	0.21261277	-2.1711590	3.701854e-02	*
AgeAtVisit	0.008113185	0.01514160	0.5358210	5.955833e-01	
APOE4_INDEX	0.575539477	0.21174397	2.7180915	1.027328e-02	*
Metabolic_Index	0.034246469	0.09855568	0.3474835	7.303780e-01	

-----

28. Results for hsa-miR-425-3p

Covariate	Beta	SE	t	p	Signif
(Intercept)	6.61575162	2.02569582	3.2659156	0.002015496	**
GroupCI	-0.51357604	0.45083259	-1.1391724	0.260275032	
SexMale	0.27136929	0.43562997	0.6229353	0.536270678	
Visit_GroupV2	0.08264673	0.32974290	0.2506399	0.803160500	
AgeAtVisit	0.03169854	0.02965309	1.0689794	0.290416443	
APOE4_INDEX	-1.09963000	0.38750799	-2.8376964	0.006635259	**
Metabolic_Index	-0.04307765	0.18400701	-0.2341088	0.815894552	

-----

29. Results for hsa-miR-144-3p

Covariate	Beta	SE	t	p	Signif
(Intercept)	9.998144906	2.43215476	4.11081773	0.0001578023	***
GroupCI	-1.089682677	0.54430928	-2.00195499	0.0511088135	.
SexMale	0.804924725	0.52605212	1.53012353	0.1327229043	
Visit_GroupV2	0.004503168	0.38457491	0.01170947	0.9907072191	
AgeAtVisit	-0.003892847	0.03576247	-0.10885286	0.9137849196	
APOE4_INDEX	0.104917052	0.46924918	0.22358495	0.8240541102	
Metabolic_Index	0.091553409	0.22354404	0.40955424	0.6840028644	

-----

30. Results for hsa-miR-769-5p

Covariate	Beta	SE	t	p	Signif
(Intercept)	5.842290074	1.03935830	5.6210549	1.872259e-07	***
GroupCI	-0.588290276	0.23057968	-2.5513535	1.231659e-02	*
SexMale	-0.105192546	0.22325364	-0.4711795	6.385859e-01	
Visit_GroupV2	-0.028299335	0.21067993	-0.1343238	8.934289e-01	

Covariate	Beta	SE	t	p	Signif
AgeAtVisit	-0.006253931	0.01514282	-0.4129964	6.805340e-01	
APOE4_INDEX	0.320255264	0.20814183	1.5386396	1.271954e-01	
Metabolic_Index	-0.088680199	0.09659373	-0.9180740	3.608903e-01	

-----

31. Results for hsa-miR-365a-3p

Covariate	Beta	SE	t	p	Signif
(Intercept)	6.84146871	1.21415050	5.6347782	1.763732e-07	***
GroupCI	-0.54256680	0.26803217	-2.0242600	4.573315e-02	*
SexMale	0.14519359	0.25947213	0.5595730	5.770793e-01	
Visit_GroupV2	-0.15569043	0.24471089	-0.6362219	5.261520e-01	
AgeAtVisit	-0.02244043	0.01758355	-1.2762168	2.049704e-01	
APOE4_INDEX	0.20890412	0.23860004	0.8755410	3.834739e-01	
Metabolic_Index	0.06398977	0.11019441	0.5806988	5.628097e-01	

-----

32. Results for hsa-miR-365b-3p

Covariate	Beta	SE	t	p	Signif
(Intercept)	6.84146871	1.21415050	5.6347782	1.763732e-07	***
GroupCI	-0.54256680	0.26803217	-2.0242600	4.573315e-02	*
SexMale	0.14519359	0.25947213	0.5595730	5.770793e-01	
Visit_GroupV2	-0.15569043	0.24471089	-0.6362219	5.261520e-01	
AgeAtVisit	-0.02244043	0.01758355	-1.2762168	2.049704e-01	
APOE4_INDEX	0.20890412	0.23860004	0.8755410	3.834739e-01	
Metabolic_Index	0.06398977	0.11019441	0.5806988	5.628097e-01	

-----

33. Results for hsa-miR-98-5p

Covariate	Beta	SE	t	p	Signif
(Intercept)	8.803740560	2.3096784	3.8116738	0.0004044857	***
GroupCI	0.785509992	0.5201810	1.5100705	0.1377898056	
SexMale	-0.130198772	0.5004783	-0.2601487	0.7958973204	
Visit_GroupV2	0.664107821	0.3122435	2.1268909	0.0387640187	*
AgeAtVisit	0.008477414	0.0338696	0.2502957	0.8034610110	
APOE4_INDEX	0.145362237	0.4422620	0.3286790	0.7438736424	
Metabolic_Index	-0.068175168	0.2130875	-0.3199398	0.7504467757	

-----

34. Results for hsa-miR-4510

Covariate	Beta	SE	t	p	Signif
(Intercept)	5.228084488	1.18083684	4.42744021	6.264277e-05	***
GroupCI	-0.555566414	0.26191562	-2.12116562	3.960666e-02	*
SexMale	0.050779765	0.25372271	0.20013882	8.422987e-01	
Visit_GroupV2	-0.253418937	0.19811843	-1.27912851	2.075892e-01	
AgeAtVisit	-0.001224845	0.01720024	-0.07121091	9.435542e-01	
APOE4_INDEX	0.221955752	0.23179297	0.95756032	3.435421e-01	
Metabolic_Index	0.077648250	0.10891944	0.71289614	4.796922e-01	

-----

35. Results for hsa-miR-150-5p

Covariate	Beta	SE	t	p	Signif
(Intercept)	10.2245583	2.06009890	4.9631395	9.256577e-06	***
GroupCI	0.4668214	0.46394792	1.0061935	3.194007e-01	
SexMale	-0.2876349	0.44817557	-0.6417906	5.240822e-01	
Visit_GroupV2	0.4554461	0.36847851	1.2360182	2.225054e-01	
AgeAtVisit	-0.0326774	0.03017826	-1.0828127	2.843362e-01	
APOE4_INDEX	0.7905219	0.38360278	2.0607824	4.480051e-02	*
Metabolic_Index	-0.1269183	0.19032761	-0.6668410	5.080873e-01	

-----

36. Results for hsa-miR-4738-3p

Covariate	Beta	SE	t	p	Signif
(Intercept)	6.25161844	1.13228343	5.5212487	2.884765e-07	***
GroupCI	-0.55162431	0.25046361	-2.2024130	3.003987e-02	*
SexMale	-0.21588147	0.23874862	-0.9042208	3.681511e-01	
Visit_GroupV2	-0.31365075	0.22708532	-1.3812022	1.704365e-01	
AgeAtVisit	-0.01063831	0.01646027	-0.6463024	5.196319e-01	
APOE4_INDEX	0.25141299	0.22395971	1.1225813	2.644265e-01	
Metabolic_Index	0.02996930	0.10362537	0.2892081	7.730483e-01	

DE miRNAs in CI vs NC: Common to both MAs and NHWs
Adjusted for the covariables in MA

37. Results for hsa-miR-6515-5p

Covariate	Beta	SE	t	p	Signif
(Intercept)	5.947205480	1.45269182	4.09392095	8.868186e-05	***
GroupCI	-0.606182408	0.31152952	-1.94582656	5.461217e-02	.
SexMale	0.099712720	0.30356695	0.32847028	7.432744e-01	
Visit_GroupV2	-0.265268324	0.28343116	-0.93591800	3.516734e-01	
AgeAtVisit	0.002177193	0.02123135	0.10254613	9.185380e-01	
APOE4_INDEX	-0.108117158	0.27650796	-0.39100920	6.966604e-01	
Metabolic_Index	-0.006336357	0.12807272	-0.04947468	9.606442e-01	

-----

38. Results for hsa-miR-199a-5p

Covariate	Beta	SE	t	p	Signif
(Intercept)	4.8619108536	1.37804854	3.528112926	0.0006451447	***
GroupCI	-0.9141314746	0.29591132	-3.089207504	0.0026278819	**
SexMale	-0.4119742243	0.28680762	-1.436413086	0.1541489896	
Visit_GroupV2	-0.0309525752	0.27468318	-0.112684639	0.9105168404	
AgeAtVisit	0.0212544166	0.02027963	1.048067301	0.2972498488	
APOE4_INDEX	-0.0007449163	0.26276924	-0.002834869	0.9977440117	
Metabolic_Index	-0.0122022267	0.12569816	-0.097075621	0.9228695496	

-----

39. Results for hsa-let-7i-3p

Covariate	Beta	SE	t	p	Signif
(Intercept)	5.2571327621	1.12369254	4.6784441	9.505906e-06	***
GroupCI	-0.7065104392	0.24686090	-2.8619779	5.172331e-03	**
SexMale	0.1964057375	0.24019439	0.8176949	4.155645e-01	
Visit_GroupV2	-0.0499650850	0.22573722	-0.2213418	8.252983e-01	
AgeAtVisit	-0.0009235458	0.01626582	-0.0567783	9.548403e-01	
APOE4_INDEX	0.2919424104	0.22002374	1.3268678	1.877127e-01	
Metabolic_Index	0.0893202840	0.10214229	0.8744692	3.840542e-01	

-----

40. Results for hsa-miR-4665-5p

Covariate	Beta	SE	t	p	Signif
(Intercept)	4.950258674	1.20065402	4.12296847	0.0001531366	***
GroupCI	- 0.447694940	0.26339592	- 1.69970339	0.0958796263	.
SexMale	0.045785460	0.25375772	0.18042982	0.8576006001	
Visit_GroupV2	- 0.505938962	0.21505336	- 2.35262064	0.0229348038	*
AgeAtVisit	0.008487518	0.01750991	0.48472656	0.6301499973	
APOE4_INDEX	- 0.017703312	0.23229678	- 0.07620989	0.9395796498	
Metabolic_Index	0.010851564	0.10924848	0.09932920	0.9213046410	

-----

41. Results for hsa-let-7g-3p

Covariate	Beta	SE	t	p	Signif
(Intercept)	5.891335030	1.07400871	5.4853699	3.366853e-07	***
GroupCI	-0.900530226	0.23635740	-3.8100362	2.456716e-04	***
SexMale	0.161969363	0.23003952	0.7040937	4.830859e-01	
Visit_GroupV2	-0.086478831	0.21610003	-0.4001796	6.899169e-01	
AgeAtVisit	-0.008708833	0.01555027	-0.5600439	5.767594e-01	
APOE4_INDEX	0.343704955	0.21051107	1.6327168	1.058178e-01	
Metabolic_Index	0.099701591	0.09779529	1.0194928	3.105419e-01	

-----

42. Results for hsa-miR-106a-5p

Covariate	Beta	SE	t	p	Signif
(Intercept)	5.1409113349	1.22054991	4.21196321	0.0001118471	***
GroupCI	-0.5753187636	0.27241224	-2.11194170	0.0399664083	*
SexMale	0.0159889556	0.26267362	0.06087005	0.9517175684	
Visit_GroupV2	-0.1818293726	0.19460981	-0.93432787	0.3548505987	
AgeAtVisit	0.0008779229	0.01779418	0.04933764	0.9608566716	
APOE4_INDEX	0.1607037369	0.24015931	0.66915473	0.5066295514	
Metabolic_Index	0.0709601526	0.11278980	0.62913626	0.5322678097	

-----

43. Results for hsa-miR-1294

Covariate	Beta	SE	t	p	Signif
(Intercept)	8.35211664	1.79213776	4.6604211	2.781371e-05	***
GroupCI	-0.59654005	0.38432403	-1.5521799	1.275631e-01	
SexMale	-0.46216478	0.37314162	-1.2385774	2.218680e-01	
Visit_GroupV2	0.24455067	0.32673705	0.7484632	4.580409e-01	
AgeAtVisit	-0.01782973	0.02636793	-0.6761902	5.023503e-01	
APOE4_INDEX	-0.19706436	0.34208300	-0.5760718	5.674116e-01	
Metabolic_Index	-0.25559416	0.16009840	-1.5964816	1.173195e-01	

-----

44. Results for hsa-miR-98-3p

Covariate	Beta	SE	t	p	Signif
(Intercept)	6.22264840	1.17885750	5.2785416	8.129454e-07	***
GroupCI	-0.77654693	0.25857598	-3.0031673	3.409979e-03	**
SexMale	0.20896848	0.25519489	0.8188584	4.149036e-01	
Visit_GroupV2	-0.11937377	0.23794380	-0.5016889	6.170397e-01	
AgeAtVisit	-0.01104489	0.01705175	-0.6477275	5.187136e-01	

Covariate	Beta	SE	t	p	Signif
APOE4_INDEX	0.30376029	0.22928628	1.3248080	1.883926e-01	
Metabolic_Index	0.05183624	0.10675507	0.4855623	6.283884e-01	

-----

45. Results for hsa-miR-7-1-3p

Covariate	Beta	SE	t	p	Signif
(Intercept)	5.28993233	1.37782596	3.8393327	0.0003764417	***
GroupCI	-0.47738518	0.29437317	-1.6217007	0.1117259216	
SexMale	-0.08853644	0.28161294	-0.3143905	0.7546508081	
Visit_GroupV2	-0.66853433	0.22986156	-2.9084216	0.0055838663	**
AgeAtVisit	0.01079361	0.02013855	0.5359677	0.5945719598	
APOE4_INDEX	-0.23391234	0.25616217	-0.9131416	0.3659455452	
Metabolic_Index	0.07368305	0.12180076	0.6049474	0.5481968520	

-----

46. Results for hsa-miR-21-3p

Covariate	Beta	SE	t	p	Signif
(Intercept)	4.49327629	1.10676025	4.0598461	0.0002060639	***
GroupCI	-0.63272311	0.24445135	-2.5883395	0.0131387156	*
SexMale	0.06757716	0.23872150	0.2830795	0.7784872618	
Visit_GroupV2	-0.43596266	0.20300069	-2.1475921	0.0374804052	*
AgeAtVisit	0.01397475	0.01614111	0.8657864	0.3914582256	
APOE4_INDEX	0.12597002	0.21818395	0.5773570	0.5667380270	
Metabolic_Index	0.01857493	0.10279399	0.1807005	0.8574597697	

-----

47. Results for hsa-miR-320e

Covariate	Beta	SE	t	p	Signif
(Intercept)	6.1091077726	1.27395503	4.79538729	5.960008e-06	***
GroupCI	-0.7513026526	0.27262367	-2.75582328	7.011062e-03	**
SexMale	-0.1134284369	0.26354254	-0.43039897	6.678741e-01	
Visit_GroupV2	-0.3165442409	0.25275054	-1.25239789	2.134798e-01	

Covariate	Beta	SE	t	p	Signif
AgeAtVisit	-0.0007044744	0.01860426	-0.03786629	9.698732e-01	
APOE4_INDEX	0.3183170154	0.24190822	1.31585860	1.913679e-01	
Metabolic_Index	0.0541418633	0.11451080	0.47281010	6.374263e-01	

-----

48. Results for hsa-miR-6877-5p

Covariate	Beta	SE	t	p	Signif
(Intercept)	4.97859315	1.10057399	4.5236333	1.746206e-05	***
GroupCI	-1.11858646	0.24525752	-4.5608651	1.510206e-05	***
SexMale	-0.13367862	0.24159662	-0.5533133	5.813405e-01	
Visit_GroupV2	-0.38841718	0.22797789	-1.7037493	9.167624e-02	.
AgeAtVisit	0.01491278	0.01605759	0.9287066	3.553799e-01	
APOE4_INDEX	0.38112894	0.22349213	1.7053349	9.137908e-02	.
Metabolic_Index	-0.06959152	0.10533053	-0.6606966	5.103958e-01	

-----

49. Results for hsa-miR-7113-5p

Covariate	Beta	SE	t	p	Signif
(Intercept)	4.70396020	1.36073076	3.45693676	0.000816949	***
GroupCI	-0.70218430	0.29701382	-2.36414688	0.020093842	*
SexMale	0.01907851	0.29160276	0.06542639	0.947971128	
Visit_GroupV2	-0.22415453	0.27380216	-0.81867336	0.415008686	
AgeAtVisit	0.01869324	0.01993979	0.93748411	0.350871767	
APOE4_INDEX	0.06355511	0.26658257	0.23840686	0.812075782	
Metabolic_Index	-0.09735127	0.12555143	-0.77538960	0.440023138	

-----

50. Results for hsa-let-7f-1-3p

Covariate	Beta	SE	t	p	Signif
(Intercept)	4.973305618	1.16067558	4.28483697	0.0001146763	***
GroupCI	-0.919966343	0.25632749	-3.58902730	0.0009099117	***
SexMale	-0.008930901	0.24610916	-0.03628837	0.9712362946	

Covariate	Beta	SE	t	p	Signif
Visit_GroupV2	0.014849065	0.22770452	0.06521199	0.9483361224	
AgeAtVisit	0.003974029	0.01685235	0.23581456	0.8148023332	
APOE4_INDEX	0.335658217	0.22684831	1.47965930	0.1469437345	
Metabolic_Index	0.213659630	0.10689286	1.99882035	0.0525844039	.

-----

51. Results for hsa-miR-381-3p

Covariate	Beta	SE	t	p	Signif
(Intercept)	4.750518458	1.10162698	4.3122750	9.036125e-05	***
GroupCI	-0.576829441	0.24179954	-2.3855688	2.144493e-02	*
SexMale	0.224439585	0.23776374	0.9439605	3.503687e-01	
Visit_GroupV2	-0.120514242	0.21366072	-0.5640449	5.756010e-01	
AgeAtVisit	0.006201198	0.01598385	0.3879666	6.999203e-01	
APOE4_INDEX	0.233562368	0.21594364	1.0815895	2.853550e-01	
Metabolic_Index	0.034934136	0.10067954	0.3469835	7.302657e-01	

-----

52. Results for hsa-miR-19a-3p

Covariate	Beta	SE	t	p	Signif
(Intercept)	5.8314360745	1.15115319	5.06573419	1.978739e-06	***
GroupCI	-0.8197962955	0.25194269	-3.25389995	1.574210e-03	**
SexMale	-0.1340723927	0.23913981	-0.56064440	5.763515e-01	
Visit_GroupV2	-0.0621598070	0.22775520	-0.27292377	7.855004e-01	
AgeAtVisit	-0.0005044183	0.01683115	-0.02996933	9.761540e-01	
APOE4_INDEX	-0.0722870613	0.22024181	-0.32821680	7.434655e-01	
Metabolic_Index	0.0735979306	0.10462680	0.70343290	4.834955e-01	

-----

53. Results for hsa-let-7a-3p

Covariate	Beta	SE	t	p	Signif
(Intercept)	6.578075556	1.41825675	4.6381415	1.114876e-05	***
GroupCI	-0.998797665	0.30674494	-3.2561178	1.563195e-03	**
SexMale	0.117041648	0.29634940	0.3949448	6.937634e-01	
Visit_GroupV2	-0.038847196	0.27991409	-0.1387826	8.899139e-01	
AgeAtVisit	-0.005421217	0.02076038	-0.2611328	7.945518e-01	
APOE4_INDEX	-0.129088392	0.26795499	-0.4817540	6.310816e-01	
Metabolic_Index	0.040534926	0.12770957	0.3173993	7.516328e-01	

-----

54. Results for hsa-miR-1303

Covariate	Beta	SE	t	p	Signif
(Intercept)	5.201463331	1.10503467	4.7070589	8.484714e-06	***
GroupCI	-0.745073648	0.24629974	-3.0250688	3.192685e-03	**
SexMale	-0.089438161	0.23894738	-0.3743007	7.090095e-01	
Visit_GroupV2	-0.230058291	0.22444426	-1.0250131	3.079435e-01	
AgeAtVisit	0.006298472	0.01611803	0.3907717	6.968354e-01	
APOE4_INDEX	0.068173643	0.21988239	0.3100459	7.572008e-01	
Metabolic_Index	-0.057139178	0.10341267	-0.5525355	5.818710e-01	

-----

55. Results for hsa-miR-3613-5p

Covariate	Beta	SE	t	p	Signif
(Intercept)	5.168393893	1.23580574	4.1822058	0.0001311498	***
GroupCI	-0.823513233	0.27044655	-3.0450129	0.0038743771	**
SexMale	0.224923448	0.26705097	0.8422491	0.4040876346	
Visit_GroupV2	-0.064008409	0.23476362	-0.2726505	0.7863649674	
AgeAtVisit	0.007018742	0.01805272	0.3887914	0.6992578110	
APOE4_INDEX	0.158452696	0.23997784	0.6602805	0.5124282518	
Metabolic_Index	0.007283671	0.11318431	0.0643523	0.9489737686	

-----

56. Results for hsa-miR-192-5p

Covariate	Beta	SE	t	p	Signif
(Intercept)	10.86689846	1.8175734	5.97879500	9.493131e-07	***
GroupCI	-0.57053142	0.3888379	-1.46727318	1.515749e-01	
SexMale	0.03065148	0.3697501	0.08289782	9.344233e-01	

Covariate	Beta	SE	t	p	Signif
Visit_GroupV2	-0.16299571	0.3232735	-0.50420379	6.173996e-01	
AgeAtVisit	-0.05798486	0.0264079	-2.19573903	3.509716e-02	*
APOE4_INDEX	-0.73089949	0.3282680	-2.22653301	3.277196e-02	*
Metabolic_Index	0.09218938	0.1604763	0.57447341	5.694635e-01	

-----

57. Results for hsa-miR-3611

Covariate	Beta	SE	t	p	Signif
(Intercept)	5.816342639	1.22582320	4.7448463	0.0000175305	***
GroupCI	-0.922847495	0.27146503	-3.3995078	0.0013259345	**
SexMale	0.045449487	0.26199732	0.1734731	0.8629730126	
Visit_GroupV2	-0.125276534	0.24088637	-0.5200649	0.6052907158	
AgeAtVisit	-0.006003423	0.01777229	-0.3377967	0.7369165031	
APOE4_INDEX	0.262719123	0.24128159	1.0888486	0.2813893803	
Metabolic_Index	0.113800747	0.11245178	1.0119960	0.3163640440	

-----

58. Results for hsa-miR-7847-3p

Covariate	Beta	SE	t	p	Signif
(Intercept)	4.19013645	1.15231448	3.6362786	0.000447958	***
GroupCI	-0.60494497	0.25408659	-2.3808615	0.019254666	*
SexMale	-0.05712041	0.24597373	-0.2322216	0.816862261	
Visit_GroupV2	-0.45997277	0.23291269	-1.9748721	0.051166985	.
AgeAtVisit	0.01945142	0.01675026	1.1612604	0.248427080	
APOE4_INDEX	0.27208122	0.22983751	1.1837981	0.239427869	
Metabolic_Index	0.02955026	0.10690419	0.2764181	0.782823599	

-----

59. Results for hsa-miR-885-5p

Covariate	Beta	SE	t	p	Signif
(Intercept)	7.31616891	1.31662053	5.55677870	2.474292e-07	***
GroupCI	-0.93685742	0.28681295	-3.26644040	1.512867e-03	**
SexMale	0.02460685	0.28181476	0.08731567	9.306032e-01	
Visit_GroupV2	0.16969658	0.26777728	0.63372285	5.277749e-01	
AgeAtVisit	-0.02693566	0.01910566	-1.40982631	1.618357e-01	
APOE4_INDEX	0.64209421	0.26011190	2.46853071	1.534276e-02	*

Covariate	Beta	SE	t	p	Signif
Metabolic_Index	0.07185313	0.11851383	0.60628482	5.457625e-01	

-----

60. Results for hsa-miR-3928-3p

Covariate	Beta	SE	t	p	Signif
(Intercept)	4.833367997	1.10258193	4.3836815	2.995527e-05	***
GroupCI	-0.578358813	0.24146468	-2.3952108	1.855921e-02	*
SexMale	0.079191681	0.23545947	0.3363283	7.373604e-01	
Visit_GroupV2	-0.185773929	0.22087593	-0.8410782	4.024028e-01	
AgeAtVisit	0.007399039	0.01604693	0.4610874	6.457831e-01	
APOE4_INDEX	0.175948352	0.21667452	0.8120399	4.187860e-01	
Metabolic_Index	0.052674235	0.10077740	0.5226790	6.024067e-01	

-----

61. Results for hsa-miR-195-5p

Covariate	Beta	SE	t	p	Signif
(Intercept)	6.969183010	1.23391519	5.64802434	1.664845e-07	***
GroupCI	-0.781485694	0.26442662	-2.95539725	3.932169e-03	**
SexMale	0.141961937	0.25776723	0.55073694	5.830986e-01	
Visit_GroupV2	-0.032322321	0.24306387	-0.13297871	8.944897e-01	
AgeAtVisit	-0.012001692	0.01805194	-0.66484209	5.077520e-01	
APOE4_INDEX	-0.089047792	0.23194228	-0.38392220	7.018886e-01	
Metabolic_Index	0.006467166	0.10970605	0.05894996	9.531150e-01	

-----

62. Results for hsa-miR-2278

Covariate	Beta	SE	t	p	Signif
(Intercept)	4.83028191	1.14217622	4.2290163	5.376043e-05	***
GroupCI	-0.83216218	0.25024335	-3.3254117	1.253241e-03	**
SexMale	0.07468414	0.24600635	0.3035862	7.621028e-01	
Visit_GroupV2	-0.13205080	0.22975737	-0.5747402	5.668170e-01	
AgeAtVisit	0.01169701	0.01667977	0.7012694	4.848383e-01	
APOE4_INDEX	0.13667499	0.22379773	0.6107077	5.428422e-01	
Metabolic_Index	0.01361458	0.10533785	0.1292468	8.974339e-01	

-----

63. Results for hsa-miR-3149

Covariate	Beta	SE	t	p	Signif
(Intercept)	4.46555979	1.30990988	3.4090588	0.00143213	**
GroupCI	-0.59581001	0.28893669	-2.0620781	0.04530801	*
SexMale	0.05112212	0.27963054	0.1828202	0.85580279	
Visit_GroupV2	-0.05102719	0.25270627	-0.2019229	0.84093420	
AgeAtVisit	0.01361807	0.01910842	0.7126740	0.47991706	
APOE4_INDEX	-0.07552257	0.25683077	-0.2940558	0.77013744	
Metabolic_Index	0.11316907	0.12040055	0.9399382	0.35252682	

-----

64. Results for hsa-miR-376b-3p

Covariate	Beta	SE	t	p	Signif
(Intercept)	5.466285800	1.23967438	4.40945292	5.670982e-05	***
GroupCI	-0.751189761	0.27571597	-2.72450584	8.906109e-03	**
SexMale	-0.130261132	0.26605884	-0.48959521	6.266085e-01	
Visit_GroupV2	-0.094522434	0.20914250	-0.45195229	6.533008e-01	
AgeAtVisit	-0.001100999	0.01805207	-0.06099014	9.516155e-01	
APOE4_INDEX	0.285965790	0.24499217	1.16724460	2.487692e-01	
Metabolic_Index	0.039360292	0.11481245	0.34282250	7.332006e-01	

-----

65. Results for hsa-miR-32-5p

Covariate	Beta	SE	t	p	Signif
(Intercept)	4.36274271	1.56965344	2.77943053	0.006556964	**
GroupCI	-0.89338388	0.33508652	-2.66612894	0.009009901	**
SexMale	0.14700741	0.33578015	0.43780851	0.662512587	
Visit_GroupV2	-0.01326892	0.31544940	-0.04206356	0.966535750	
AgeAtVisit	0.03072992	0.02296324	1.33822269	0.183997819	
APOE4_INDEX	-0.02453792	0.29299573	-0.08374840	0.933431579	
Metabolic_Index	-0.06439828	0.14105781	-0.45653818	0.649038493	

-----

66. Results for hsa-let-7c-5p

Covariate	Beta	SE	t	p	Signif
(Intercept)	10.21333210	1.36756330	7.4682701	1.049589e-09	***

Covariate	Beta	SE	t	p	Signif
GroupCI	0.97677947	0.31293515	3.1213479	2.975485e-03	**
SexMale	-0.81010024	0.30238480	-2.6790375	9.939780e-03	**
Visit_GroupV2	0.16583896	0.22830912	0.7263790	4.709591e-01	
AgeAtVisit	0.01478089	0.01993518	0.7414476	4.618555e-01	
APOE4_INDEX	-0.09442753	0.26018926	-0.3629186	7.181799e-01	
Metabolic_Index	-0.26196171	0.12648678	-2.0710601	4.348104e-02	*

-----

67. Results for hsa-miR-627-5p

Covariate	Beta	SE	t	p	Signif
(Intercept)	6.652319710	1.4290451	4.6550802	1.042724e-05	***
GroupCI	-0.561373693	0.2987558	-1.8790385	6.328535e-02	.
SexMale	0.220443572	0.2970566	0.7420929	4.598503e-01	
Visit_GroupV2	-0.776587872	0.2772558	-2.8009792	6.166087e-03	**
AgeAtVisit	-0.004496164	0.0208353	-0.2157955	8.296068e-01	
APOE4_INDEX	-0.048832710	0.2627818	-0.1858299	8.529719e-01	
Metabolic_Index	0.131962436	0.1243487	1.0612290	2.912593e-01	

-----

68. Results for hsa-miR-19b-3p

Covariate	Beta	SE	t	p	Signif
(Intercept)	7.74120104	2.00641213	3.85823077	0.0003401759	***
GroupCI	-1.04083707	0.42799780	-2.43187484	0.0188003598	*
SexMale	-0.20068432	0.41461648	-0.48402398	0.6305692099	
Visit_GroupV2	0.14774768	0.34391080	0.42961047	0.6694007930	
AgeAtVisit	-0.01558493	0.02922558	-0.53326349	0.5963110279	
APOE4_INDEX	-0.03096092	0.36805953	-0.08411932	0.9333113087	
Metabolic_Index	0.14990950	0.17963419	0.83452652	0.4081172306	

-----

69. Results for hsa-miR-651-5p

Covariate	Beta	SE	t	p	Signif
(Intercept)	3.67727562	1.44994609	2.5361464	0.01282835	*
GroupCI	-0.58722520	0.31315300	-1.8752022	0.06381683	.
SexMale	0.05852402	0.30965951	0.1889948	0.85049755	
Visit_GroupV2	0.04432263	0.28989315	0.1528930	0.87880488	

Covariate	Beta	SE	t	p	Signif
AgeAtVisit	0.03223935	0.02128757	1.5144684	0.13320610	
APOE4_INDEX	-0.15669576	0.27593349	-0.5678751	0.57145097	
Metabolic_Index	0.05303520	0.13258191	0.4000184	0.69003530	

-----

70. Results for hsa-miR-760

Covariate	Beta	SE	t	p	Signif
(Intercept)	4.58181224	1.30702522	3.50552703	0.0009105002	***
GroupCI	-0.56100137	0.27938794	-2.00796560	0.0495309365	*
SexMale	0.23329486	0.26889006	0.86762176	0.3893426229	
Visit_GroupV2	-0.20869868	0.25176925	-0.82892838	0.4107007251	
AgeAtVisit	0.01444812	0.01905744	0.75813532	0.4515817841	
APOE4_INDEX	0.00325317	0.24506089	0.01327495	0.9894561086	
Metabolic_Index	0.18687563	0.11608624	1.60980004	0.1131232221	

-----

71. Results for hsa-miR-431-3p

Covariate	Beta	SE	t	p	Signif
(Intercept)	4.9467911825	1.22697073	4.031710819	0.0002725201	***
GroupCI	-0.7991836020	0.26884118	-2.972697871	0.0052203789	**
SexMale	-0.0132695928	0.25956103	-0.051123209	0.9595080934	
Visit_GroupV2	-0.2287105619	0.24050474	-0.950960739	0.3479238070	
AgeAtVisit	0.0132213488	0.01800383	0.734363102	0.4674527276	
APOE4_INDEX	-0.0004972942	0.23750299	-0.002093844	0.9983408392	
Metabolic_Index	-0.0034883432	0.11175176	-0.031215107	0.9752692756	

-----

72. Results for hsa-miR-5010-5p

Covariate	Beta	SE	t	p	Signif
(Intercept)	9.62667792	1.85860759	5.1795107	3.766862e-06	***
GroupCI	-0.66627637	0.39927995	-1.6686948	1.012669e-01	
SexMale	0.17337891	0.39442652	0.4395722	6.620921e-01	
Visit_GroupV2	-0.48976469	0.31382017	-1.5606540	1.247501e-01	
AgeAtVisit	-0.03849337	0.02717066	-1.4167258	1.626036e-01	
APOE4_INDEX	-0.04548668	0.35279954	-0.1289307	8.979164e-01	
Metabolic_Index	-0.19246493	0.16297180	-1.1809708	2.430574e-01	

-----

73. Results for hsa-let-7a-5p

Covariate	Beta	SE	t	p	Signif
(Intercept)	16.09551842	1.25399495	12.8353933	1.382892e-17	***
GroupCI	0.63206254	0.28397996	2.2257294	3.048623e-02	*
SexMale	0.25763630	0.27452817	0.9384694	3.524331e-01	
Visit_GroupV2	0.20899083	0.15819377	1.3211066	1.923714e-01	
AgeAtVisit	-0.01138049	0.01839759	-0.6185857	5.389465e-01	
APOE4_INDEX	0.09483487	0.24326326	0.3898446	6.982772e-01	
Metabolic_Index	-0.01707664	0.11697356	-0.1459872	8.845079e-01	

-----

74. Results for hsa-miR-181a-5p

Covariate	Beta	SE	t	p	Signif
(Intercept)	8.09539751	1.8813917	4.3028771	4.072307e-05	***
GroupCI	0.78338594	0.3766270	2.0800045	4.020061e-02	*
SexMale	0.51028387	0.3615331	1.4114443	1.613597e-01	
Visit_GroupV2	0.27934869	0.3443678	0.8111929	4.192698e-01	
AgeAtVisit	-0.03737631	0.0274401	-1.3621054	1.763652e-01	
APOE4_INDEX	0.64935025	0.3325554	1.9526080	5.379070e-02	.
Metabolic_Index	0.19551995	0.1556290	1.2563209	2.120607e-01	

-----

75. Results for hsa-miR-376c-3p

Covariate	Beta	SE	t	p	Signif
(Intercept)	4.22130656	1.54491283	2.7323914	0.007489978	**
GroupCI	-0.51522561	0.31747193	-1.6229013	0.107903411	
SexMale	0.13691214	0.30755610	0.4451615	0.657209291	
Visit_GroupV2	-0.19319740	0.29188344	-0.6618991	0.509628077	
AgeAtVisit	0.02576482	0.02256875	1.1416152	0.256465208	
APOE4_INDEX	-0.22599356	0.27405845	-0.8246181	0.411641026	
Metabolic_Index	0.19653415	0.13439489	1.4623633	0.146921096	
