# Supplementary material for: Neuronal Enriched Extracellular Vesicle miR-122-5p as a Potential Biomarker for Alzheimer’s Disease
Source: Cells. 2025 Nov 13;14(22):1784. doi: 10.3390/cells14221784 (PMC12651308; doi:10.3390/cells14221784)
Supplement: Supplementary file 1 [file cells-14-01784-s001.zip › Supplementary Document S4.pdf]

Supplementary Document S4

Non-Hispanic Whites

Model Statistics from DREAM after accounting for the repeated measures due to longitudinal data points

1. Results for hsa-miR-122-5p

Covariate	Beta	SE	t	p	Signif
(Intercept)	13.97325124	2.30038796	6.0743020	1.217452e-07	***
GroupCI	-1.07944341	0.43012785	-2.5095873	1.505887e-02	*
SexMale	1.22413273	0.43536443	2.8117426	6.814860e-03	**
Visit_GroupV2	-0.60764152	0.21897303	-2.7749606	7.526780e-03	**
AgeAtVisit	0.02510742	0.03213574	0.7812926	4.379784e-01	
APOE4_INDEX	-0.07356882	0.30593405	-0.2404728	8.108573e-01	
Metabolic_Index	-0.03651386	0.17013370	-0.2146186	8.308579e-01	

DE miRNAs in CI vs NC unique to Non-Hispanic Whites

2. Results for hsa-miR-3679-5p

Covariate	Beta	SE	t	p	Signif
(Intercept)	3.60003367	1.53177458	2.3502372	2.072038e-02	*
GroupCI	1.29009841	0.29948780	4.3076827	3.855258e-05	***
SexMale	-0.31141539	0.28465485	-1.0940105	2.765724e-01	
Visit_GroupV2	0.40615903	0.27796227	1.4612020	1.470878e-01	
AgeAtVisit	0.02228793	0.02084854	1.0690403	2.876192e-01	
APOE4_INDEX	0.14984411	0.19933758	0.7517103	4.539867e-01	
Metabolic_Index	0.22053078	0.11867794	1.8582288	6.607116e-02	.

-----

3. Results for hsa-miR-99b-3p

Covariate	Beta	SE	t	p	Signif
(Intercept)	3.57435437	1.63668874	2.1838938	3.414881e-02	*
GroupCI	1.38049641	0.31618447	4.3661108	7.189143e-05	***

Covariate	Beta	SE	t	p	Signif
SexMale	-0.25199883	0.30456885	-0.8273953	4.123192e-01	
Visit_GroupV2	0.31085333	0.26803696	1.1597405	2.521868e-01	
AgeAtVisit	0.01929163	0.02239853	0.8612900	3.935817e-01	
APOE4_INDEX	0.16222130	0.21231228	0.7640693	4.487589e-01	
Metabolic_Index	0.14389544	0.12408295	1.1596714	2.522147e-01	

-----

4. Results for hsa-miR-5187-5p

Covariate	Beta	SE	t	p	Signif
(Intercept)	5.73176772	1.82784939	3.1357987	0.0029132983	**
GroupCI	1.34365525	0.35044222	3.8341706	0.0003641496	***
SexMale	0.14330409	0.33689024	0.4253732	0.6724507898	
Visit_GroupV2	0.73446530	0.28815067	2.5488932	0.0140318519	*
AgeAtVisit	-0.01446557	0.02501644	-0.5782425	0.5657823850	
APOE4_INDEX	0.22367976	0.23584268	0.9484278	0.3476252281	
Metabolic_Index	0.21057466	0.13795818	1.5263659	0.1334301885	

-----

5. Results for hsa-miR-1275

Covariate	Beta	SE	t	p	Signif
(Intercept)	2.77752677	1.77468506	1.5650815	1.239831e-01	
GroupCI	1.51672515	0.34018555	4.4585232	4.793524e-05	***
SexMale	-0.42999578	0.33205742	-1.2949441	2.013923e-01	
Visit_GroupV2	0.02791416	0.24480851	0.1140245	9.096831e-01	
AgeAtVisit	0.02369071	0.02453502	0.9655874	3.389800e-01	
APOE4_INDEX	0.21081095	0.23104507	0.9124235	3.660017e-01	
Metabolic_Index	0.33998929	0.13385238	2.5400318	1.429644e-02	*

-----

6. Results for hsa-miR-9-5p

Covariate	Beta	SE	t	p	Signif
(Intercept)	3.68551064	2.1069406	1.7492238	0.0862380678	.
GroupCI	1.63043688	0.3995511	4.0806721	0.0001573024	***
SexMale	-0.44510643	0.3925209	-1.1339686	0.2620833735	
Visit_GroupV2	-0.12155684	0.2608707	-0.4659659	0.6432140147	
AgeAtVisit	0.01015788	0.0291575	0.3483795	0.7289819906	

Covariate	Beta	SE	t	p	Signif
APOE4_INDEX	0.26188157	0.2732381	0.9584373	0.3423413851	
Metabolic_Index	0.48858521	0.1575965	3.1002292	0.0031389578	**

-----

7. Results for hsa-miR-200a-3p

Covariate	Beta	SE	t	p	Signif
(Intercept)	2.94355496	1.85095156	1.5902928	0.1178771178	
GroupCI	1.44721801	0.35156078	4.1165514	0.0001389906	***
SexMale	-0.45476946	0.34472778	-1.3192133	0.1929215618	
Visit_GroupV2	-0.14255242	0.21545046	-0.6616482	0.5111404059	
AgeAtVisit	0.02211433	0.02560784	0.8635766	0.3918166645	
APOE4_INDEX	0.26907868	0.24007997	1.1207877	0.2675670549	
Metabolic_Index	0.37879666	0.13847097	2.7355672	0.0085167123	**

-----

8. Results for hsa-miR-548j-5p

Covariate	Beta	SE	t	p	Signif
(Intercept)	2.69081347	1.82629931	1.4733694	0.1467889473	
GroupCI	1.35115113	0.34739746	3.8893523	0.0002916595	***
SexMale	-0.28926367	0.33952534	-0.8519649	0.3982113190	
Visit_GroupV2	-0.10940399	0.22345188	-0.4896087	0.6265074215	
AgeAtVisit	0.02446119	0.02525867	0.9684273	0.3373952915	
APOE4_INDEX	0.24924439	0.23653470	1.0537329	0.2969654051	
Metabolic_Index	0.36054966	0.13677836	2.6360138	0.0110828457	*

-----

9. Results for hsa-miR-3150b-3p

Covariate	Beta	SE	t	p	Signif
(Intercept)	4.553583818	1.9962585	2.2810592	0.0267764854	*
GroupCI	1.396851567	0.3797158	3.6786761	0.0005672777	***
SexMale	-0.536758010	0.3711856	-1.4460637	0.1543094621	
Visit_GroupV2	-0.163882547	0.2815902	-0.5819894	0.5631513151	
AgeAtVisit	0.004195212	0.0273988	0.1531166	0.8789137166	
APOE4_INDEX	0.244463509	0.2584720	0.9458026	0.3487325115	
Metabolic_Index	0.431403469	0.1498342	2.8792061	0.0058240317	**

-----

10. Results for hsa-miR-590-5p

Covariate	Beta	SE	t	p	Signif
(Intercept)	2.92317030	1.77701845	1.6449859	0.1061461873	
GroupCI	1.35721602	0.33885098	4.0053478	0.0002023761	***
SexMale	-0.37313925	0.32974160	-1.1316111	0.2631106248	
Visit_GroupV2	0.11521565	0.22296950	0.5167328	0.6075846815	
AgeAtVisit	0.02540092	0.02454278	1.0349651	0.3055860701	
APOE4_INDEX	0.05833710	0.22960601	0.2540748	0.8004631663	
Metabolic_Index	0.33532878	0.13396054	2.5031908	0.0155646808	*

-----

11. Results for hsa-miR-299-3p

Covariate	Beta	SE	t	p	Signif
(Intercept)	1.73248861	1.91569770	0.9043643	0.3701165779	
GroupCI	1.49608576	0.36504747	4.0983321	0.0001516743	***
SexMale	-0.44883658	0.35679405	-1.2579710	0.2142149740	
Visit_GroupV2	0.09822452	0.23246207	0.4225400	0.6744330525	
AgeAtVisit	0.04090987	0.02652972	1.5420391	0.1293369680	
APOE4_INDEX	0.17882061	0.24811137	0.7207272	0.4744161859	
Metabolic_Index	0.25781736	0.14356219	1.7958584	0.0785290042	.

-----

12. Results for hsa-miR-374a-3p

Covariate	Beta	SE	t	p	Signif
(Intercept)	2.91992639	1.97023384	1.4820202	0.1444970597	
GroupCI	1.41080598	0.37489911	3.7631617	0.0004350231	***
SexMale	-0.16285653	0.36537497	-0.4457244	0.6576856616	
Visit_GroupV2	-0.09827282	0.22574832	-0.4353203	0.6651695209	
AgeAtVisit	0.02098952	0.02727397	0.7695807	0.4451038760	
APOE4_INDEX	0.14702314	0.25462240	0.5774164	0.5662025296	
Metabolic_Index	0.36727115	0.14772305	2.4862142	0.0162326388	*

-----

13. Results for hsa-miR-503-5p

Covariate	Beta	SE	t	p	Signif
(Intercept)	2.707402305	2.03457258	1.33069831	0.1892097504	
GroupCI	1.429289860	0.38928306	3.67159531	0.0005776821	***
SexMale	0.008521016	0.37951355	0.02245247	0.9821746779	
Visit_GroupV2	0.206143726	0.22004741	0.93681504	0.3532706242	
AgeAtVisit	0.026865284	0.02819765	0.95274905	0.3452128500	
APOE4_INDEX	0.161710694	0.26356105	0.61356066	0.5422337548	
Metabolic_Index	0.198632629	0.15293030	1.29884419	0.1998415555	

-----

14. Results for hsa-miR-548d-5p

Covariate	Beta	SE	t	p	Signif
(Intercept)	3.419483585	2.10391929	1.62529219	0.1101202114	
GroupCI	1.428865311	0.39960019	3.57573735	0.0007626185	***
SexMale	-0.208726364	0.39086596	-0.53401008	0.5956002555	
Visit_GroupV2	0.008558904	0.23449099	0.03649992	0.9710228876	
AgeAtVisit	0.014661646	0.02912989	0.50331972	0.6168556876	
APOE4_INDEX	0.183246942	0.27188320	0.67399141	0.5032889122	
Metabolic_Index	0.331315318	0.15725787	2.10682821	0.0399536633	*

-----

15. Results for hsa-miR-4667-5p

Covariate	Beta	SE	t	p	Signif
(Intercept)	3.0815753	1.85366904	1.6624194	0.1031317243	
GroupCI	1.2546325	0.35427173	3.5414413	0.0009148823	***
SexMale	-0.3512744	0.34532830	-1.0172187	0.3142939211	
Visit_GroupV2	-0.1513747	0.26999665	-0.5606540	0.5777152696	
AgeAtVisit	0.0222195	0.02552083	0.8706415	0.3884063584	
APOE4_INDEX	0.2152845	0.24064988	0.8945963	0.3755935588	
Metabolic_Index	0.4050571	0.14039068	2.8852135	0.0059036207	**

-----

16. Results for hsa-miR-548ad-5p

Covariate	Beta	SE	t	p	Signif
(Intercept)	3.46103968	2.02759528	1.7069677	0.0937662885	.
GroupCI	1.36860739	0.38352545	3.5684917	0.0007796376	***

Covariate	Beta	SE	t	p	Signif
SexMale	-0.17764084	0.37584044	-0.4726496	0.6384319693	
Visit_GroupV2	0.07977361	0.21929291	0.3637765	0.7174930568	
AgeAtVisit	0.01098398	0.02808761	0.3910613	0.6973424128	
APOE4_INDEX	0.21392861	0.26153225	0.8179818	0.4170831217	
Metabolic_Index	0.40383521	0.15122893	2.6703569	0.0100764461	*

-----

17. Results for hsa-miR-548ae-5p

Covariate	Beta	SE	t	p	Signif
(Intercept)	3.46103968	2.02759528	1.7069677	0.0937662885	.
GroupCI	1.36860739	0.38352545	3.5684917	0.0007796376	***
SexMale	-0.17764084	0.37584044	-0.4726496	0.6384319693	
Visit_GroupV2	0.07977361	0.21929291	0.3637765	0.7174930568	
AgeAtVisit	0.01098398	0.02808761	0.3910613	0.6973424128	
APOE4_INDEX	0.21392861	0.26153225	0.8179818	0.4170831217	
Metabolic_Index	0.40383521	0.15122893	2.6703569	0.0100764461	*

-----

18. Results for hsa-miR-1224-5p

Covariate	Beta	SE	t	p	Signif
(Intercept)	1.90821880	1.63284433	1.1686471	0.2491914055	
GroupCI	1.15388675	0.31803571	3.6281672	0.0007728656	***
SexMale	-0.30530423	0.30657363	-0.9958594	0.3250691822	
Visit_GroupV2	-0.26173998	0.28123036	-0.9306960	0.3573742198	
AgeAtVisit	0.03965357	0.02243624	1.7673892	0.0844893470	.
APOE4_INDEX	0.45279484	0.21556931	2.1004606	0.0417838357	*
Metabolic_Index	0.41768914	0.12573886	3.3218779	0.0018685530	**

-----

19. Results for hsa-miR-548ay-5p

Covariate	Beta	SE	t	p	Signif
(Intercept)	3.367147306	2.10010426	1.60332388	0.114900742	
GroupCI	1.388425926	0.39872972	3.48212295	0.001014879	**
SexMale	-0.171526283	0.39004326	-0.43976220	0.661925697	
Visit_GroupV2	0.006941245	0.23442351	0.02960985	0.976491187	
AgeAtVisit	0.014771374	0.02907731	0.50800345	0.613592093	

Covariate	Beta	SE	t	p	Signif
APOE4_INDEX	0.206672935	0.27136810	0.76159628	0.449730067	
Metabolic_Index	0.343495712	0.15696956	2.18829510	0.033148657	*

-----

20. Results for hsa-miR-27b-5p

Covariate	Beta	SE	t	p	Signif
(Intercept)	2.40355687	1.77071381	1.3573943	0.1807398328	
GroupCI	1.24696552	0.33619406	3.7090647	0.0005214665	***
SexMale	-0.51508401	0.32989177	-1.5613727	0.1247315462	
Visit_GroupV2	-0.12685055	0.23984336	-0.5288892	0.5992180047	
AgeAtVisit	0.03004435	0.02442677	1.2299762	0.2244509690	
APOE4_INDEX	0.36824997	0.23011128	1.6003126	0.1158175304	
Metabolic_Index	0.44059965	0.13306915	3.3110578	0.0017288328	**

-----

21. Results for hsa-let-7b-3p

Covariate	Beta	SE	t	p	Signif
(Intercept)	2.24176962	1.70906992	1.3116898	0.196847401	
GroupCI	1.18497607	0.33480146	3.5393396	0.001005939	**
SexMale	0.04698545	0.31964672	0.1469918	0.883851473	
Visit_GroupV2	0.10679464	0.28580287	0.3736654	0.710558708	
AgeAtVisit	0.04143627	0.02343224	1.7683445	0.084366502	.
APOE4_INDEX	0.49746320	0.22444520	2.2164128	0.032211120	*
Metabolic_Index	0.20051986	0.13196415	1.5195026	0.136231656	

-----

22. Results for hsa-miR-4750-5p

Covariate	Beta	SE	t	p	Signif
(Intercept)	4.025993838	1.61983460	2.4854351	0.016365440	*
GroupCI	0.986548942	0.31203352	3.1616762	0.002678397	**
SexMale	0.169265498	0.30226882	0.5599833	0.578017445	
Visit_GroupV2	0.348443866	0.24906489	1.3990084	0.168048204	
AgeAtVisit	0.006419428	0.02222284	0.2888662	0.773890606	
APOE4_INDEX	0.540558876	0.21196575	2.5502180	0.013910244	*
Metabolic_Index	0.225314652	0.12264783	1.8370863	0.072206957	.

-----

23. Results for hsa-miR-374b-5p

Covariate	Beta	SE	t	p	Signif
(Intercept)	3.67071804	1.95922432	1.8735568	0.0665347241	.
GroupCI	1.36329868	0.37014314	3.6831661	0.0005432129	***
SexMale	-0.52746443	0.36367093	-1.4503893	0.1528691083	
Visit_GroupV2	-0.07230036	0.20584071	-0.3512442	0.7268038837	
AgeAtVisit	0.01301974	0.02710528	0.4803396	0.6329714983	
APOE4_INDEX	0.14933103	0.25245605	0.5915130	0.5567027339	
Metabolic_Index	0.35660169	0.14577983	2.4461661	0.0178063448	*

-----

24. Results for hsa-miR-374b-3p

Covariate	Beta	SE	t	p	Signif
(Intercept)	3.66388040	1.80486230	2.0300055	0.047643289	*
GroupCI	1.19605465	0.34249685	3.4921625	0.001005528	**
SexMale	-0.30961439	0.33557531	-0.9226376	0.360585970	
Visit_GroupV2	0.04157310	0.23481855	0.1770435	0.860182815	
AgeAtVisit	0.01075332	0.02490795	0.4317224	0.667781923	
APOE4_INDEX	0.29126844	0.23360985	1.2468157	0.218220536	
Metabolic_Index	0.37477546	0.13522932	2.7714069	0.007795425	**

-----

25. Results for hsa-miR-7111-5p

Covariate	Beta	SE	t	p	Signif
(Intercept)	3.02976263	2.2059021	1.37348010	0.175861081	
GroupCI	1.44580822	0.4194890	3.44659406	0.001175033	**
SexMale	-0.37264365	0.4124968	-0.90338553	0.370745460	
Visit_GroupV2	0.02622529	0.2684478	0.09769232	0.922575522	
AgeAtVisit	0.01774527	0.0305511	0.58083901	0.564013643	
APOE4_INDEX	0.47322622	0.2923962	1.61844165	0.111993439	
Metabolic_Index	0.50562490	0.1660413	3.04517467	0.003737841	**

-----

26. Results for hsa-miR-654-3p

Covariate	Beta	SE	t	p	Signif
(Intercept)	3.4419359	1.98614585	1.7329724	0.089000909	.
GroupCI	1.3522497	0.37742246	3.5828543	0.000746077	***
SexMale	-0.3101815	0.36918861	-0.8401708	0.404641748	
Visit_GroupV2	0.2092388	0.21152639	0.9891853	0.327132439	
AgeAtVisit	0.0193518	0.02749522	0.7038242	0.484667935	
APOE4_INDEX	0.1264124	0.25618050	0.4934504	0.623762704	
Metabolic_Index	0.2202841	0.14836971	1.4846970	0.143633970	

-----

27. Results for hsa-miR-1306-5p

Covariate	Beta	SE	t	p	Signif
(Intercept)	6.403491032	1.67688691	3.81867793	0.0003529506	***
GroupCI	0.945001508	0.31469172	3.00294365	0.0040711103	**
SexMale	0.062327985	0.29952869	0.20808686	0.8359573099	
Visit_GroupV2	0.817160389	0.28983565	2.81939230	0.0067483579	**
AgeAtVisit	-<br>0.017752361	0.02270001	-<br>0.78204199	0.4376636956	
APOE4_INDEX	-<br>0.002535321	0.20827032	-<br>0.01217322	0.9903330399	
Metabolic_Index	0.186901582	0.12549030	1.48937081	0.1423062954	

-----

28. Results for hsa-miR-127-3p

Covariate	Beta	SE	t	p	Signif
(Intercept)	2.54886103	1.88587697	1.3515521	0.1824136361	
GroupCI	1.27822897	0.35779347	3.5725330	0.0007764556	***
SexMale	-0.27821644	0.35012602	-0.7946180	0.4304750537	
Visit_GroupV2	-0.22766285	0.22121034	-1.0291691	0.3081998928	
AgeAtVisit	0.02801573	0.02609888	1.0734454	0.2880652486	
APOE4_INDEX	0.18632900	0.24344741	0.7653768	0.4475336004	
Metabolic_Index	0.42550972	0.14105907	3.0165357	0.0039625282	**

-----

29. Results for hsa-miR-326

Covariate	Beta	SE	t	p	Signif
(Intercept)	3.20490150	1.94464981	1.6480610	0.10589145	

Covariate	Beta	SE	t	p	Signif
GroupCI	1.38855774	0.37287050	3.7239678	0.00051675	***
SexMale	-0.07350234	0.36331587	-0.2023097	0.84053225	
Visit_GroupV2	0.38161166	0.24945390	1.5297883	0.13265329	
AgeAtVisit	0.02947091	0.02682794	1.0985156	0.27747580	
APOE4_INDEX	0.05145612	0.25152984	0.2045726	0.83877367	
Metabolic_Index	0.07331978	0.14623326	0.5013893	0.61839605	

-----

30. Results for hsa-miR-6124

Covariate	Beta	SE	t	p	Signif
(Intercept)	2.56673495	1.97235049	1.3013584	0.199039969	
GroupCI	1.28919599	0.37364125	3.4503577	0.001139275	**
SexMale	-0.32065422	0.36619421	-0.8756398	0.385370635	
Visit_GroupV2	0.11131970	0.23983144	0.4641581	0.644528670	
AgeAtVisit	0.02391371	0.02730551	0.8757834	0.385293306	
APOE4_INDEX	0.38070558	0.25495346	1.4932356	0.141595627	
Metabolic_Index	0.38884812	0.14747624	2.6366833	0.011094827	*

-----

31. Results for hsa-miR-664b-5p

Covariate	Beta	SE	t	p	Signif
(Intercept)	1.33140396	1.64372677	0.80999105	0.4198641095	
GroupCI	1.18509515	0.32379721	3.65999181	0.0004045482	***
SexMale	-0.02842029	0.31048881	-0.09153401	0.9272509603	
Visit_GroupV2	0.01269633	0.30586205	0.04151000	0.9669718940	
AgeAtVisit	0.04338825	0.02265974	1.91477252	0.0583726073	.
APOE4_INDEX	0.27838302	0.21708352	1.28237751	0.2026680360	
Metabolic_Index	0.49738527	0.12977461	3.83268552	0.0002213589	***

-----

32. Results for hsa-miR-493-3p

Covariate	Beta	SE	t	p	Signif
(Intercept)	3.25346490	1.84598891	1.7624509	0.084075120	.
GroupCI	1.21108061	0.35154874	3.4449864	0.001161554	**
SexMale	-0.37567782	0.34425181	-1.0912878	0.280351658	
Visit_GroupV2	0.03529867	0.22333785	0.1580506	0.875050440	

Covariate	Beta	SE	t	p	Signif
AgeAtVisit	0.01915635	0.02550955	0.7509483	0.456186444	
APOE4_INDEX	0.41699613	0.24138762	1.7274960	0.090221472	.
Metabolic_Index	0.27109683	0.13796678	1.9649428	0.054961877	.

-----

33. Results for hsa-miR-7977

Covariate	Beta	SE	t	p	Signif
(Intercept)	2.589337783	1.77550692	1.45836536	0.151559622	
GroupCI	1.155459811	0.34143046	3.38417319	0.001471554	**
SexMale	-0.053063049	0.33141335	-0.16011138	0.873496978	
Visit_GroupV2	-0.007991737	0.26653173	-0.02998419	0.976209865	
AgeAtVisit	0.025631080	0.02446435	1.04769124	0.300275074	
APOE4_INDEX	0.250304676	0.23124131	1.08243928	0.284720039	
Metabolic_Index	0.428416324	0.13532329	3.16587289	0.002748226	**

-----

34. Results for hsa-miR-590-3p

Covariate	Beta	SE	t	p	Signif
(Intercept)	3.89434120	2.03405268	1.9145724	0.060994752	.
GroupCI	1.32412165	0.38243835	3.4623140	0.001072286	**
SexMale	-0.40866128	0.37508123	-1.0895274	0.280889293	
Visit_GroupV2	0.09931525	0.21900344	0.4534872	0.652063941	
AgeAtVisit	0.01380309	0.02810759	0.4910806	0.625412805	
APOE4_INDEX	0.21451279	0.25998385	0.8251004	0.413042033	
Metabolic_Index	0.24840458	0.15028730	1.6528647	0.104317472	

-----

35. Results for hsa-miR-10a-3p

Covariate	Beta	SE	t	p	Signif
(Intercept)	2.66354831	1.93977361	1.3731233	0.175601028	
GroupCI	1.27456300	0.36715118	3.4714937	0.001049566	**
SexMale	-0.30585554	0.36030268	-0.8488850	0.399832865	
Visit_GroupV2	-0.21992254	0.20732170	-1.0607792	0.293688053	
AgeAtVisit	0.02585886	0.02686043	0.9627122	0.340143597	
APOE4_INDEX	0.17841114	0.25048355	0.7122669	0.479480384	
Metabolic_Index	0.46521655	0.14497164	3.2090176	0.002281885	**

-----

36. Results for hsa-miR-3150a-3p

Covariate	Beta	SE	t	p	Signif
(Intercept)	3.21147844	1.94637889	1.6499760	0.104965708	
GroupCI	1.25876298	0.36809830	3.4196381	0.001226059	**
SexMale	-0.43977795	0.36128135	-1.2172728	0.228986368	
Visit_GroupV2	-0.11031448	0.21705137	-0.5082414	0.613429739	
AgeAtVisit	0.01833717	0.02692181	0.6811269	0.498808071	
APOE4_INDEX	0.25457756	0.25158260	1.0119045	0.316261007	
Metabolic_Index	0.37240567	0.14501284	2.5680876	0.013133335	*

-----

37. Results for hsa-miR-6089

Covariate	Beta	SE	t	p	Signif
(Intercept)	3.517361649	1.97860307	1.7776995	0.081392493	.
GroupCI	1.229529597	0.37226308	3.3028513	0.001750181	**
SexMale	-0.212898652	0.36612834	-0.5814864	0.563464079	
Visit_GroupV2	0.111624549	0.23295299	0.4791720	0.633856573	
AgeAtVisit	0.008675195	0.02738846	0.3167464	0.752723922	
APOE4_INDEX	0.309652660	0.25451268	1.2166492	0.229316525	
Metabolic_Index	0.491526195	0.14725640	3.3378935	0.001578764	**

-----

38. Results for hsa-miR-625-3p

Covariate	Beta	SE	t	p	Signif
(Intercept)	4.319344920	1.97893896	2.1826570	0.033508526	*
GroupCI	1.239421154	0.37443948	3.3100707	0.001681552	**
SexMale	-0.268004912	0.36719318	-0.7298744	0.468677599	
Visit_GroupV2	0.179923897	0.20326036	0.8851893	0.380049137	
AgeAtVisit	0.004551422	0.02740001	0.1661102	0.868701658	
APOE4_INDEX	0.186312187	0.25461100	0.7317523	0.467539991	
Metabolic_Index	0.299060702	0.14736928	2.0293286	0.047454928	*

-----

39. Results for hsa-miR-491-5p

Covariate	Beta	SE	t	p	Signif
(Intercept)	2.98652951	1.89512446	1.5759015	0.121148255	
GroupCI	1.20679828	0.35956891	3.3562364	0.001487358	**
SexMale	-0.22290049	0.35249865	-0.6323442	0.529948565	
Visit_GroupV2	0.19122903	0.22111754	0.8648298	0.391127199	
AgeAtVisit	0.01923379	0.02622263	0.7334805	0.466578389	
APOE4_INDEX	0.22335074	0.24520819	0.9108616	0.366595501	
Metabolic_Index	0.34608955	0.14176571	2.4412782	0.018095697	*

-----

40. Results for hsa-miR-652-5p

Covariate	Beta	SE	t	p	Signif
(Intercept)	2.60463147	1.94102492	1.3418846	0.185500136	
GroupCI	1.22438208	0.36842787	3.3232613	0.001640075	**
SexMale	-0.44236368	0.36170869	-1.2229833	0.226884161	
Visit_GroupV2	0.09712173	0.24350545	0.3988483	0.691648426	
AgeAtVisit	0.02674596	0.02683253	0.9967737	0.323519113	
APOE4_INDEX	0.30718699	0.25152137	1.2213156	0.227509351	
Metabolic_Index	0.36832358	0.14552521	2.5309950	0.014457972	*

-----

41. Results for hsa-miR-3940-3p

Covariate	Beta	SE	t	p	Signif
(Intercept)	3.910291396	1.83412905	2.1319609	0.037863852	*
GroupCI	1.142246076	0.34744074	3.2875997	0.001837007	**
SexMale	-0.390471278	0.34070200	-1.1460786	0.257128609	
Visit_GroupV2	0.031199053	0.23373188	0.1334822	0.894339003	
AgeAtVisit	0.009436901	0.02529367	0.3730934	0.710630083	
APOE4_INDEX	0.228568080	0.23683687	0.9650865	0.339069968	
Metabolic_Index	0.379493170	0.13731706	2.7636272	0.007940759	**

-----

42. Results for hsa-miR-500a-3p

Covariate	Beta	SE	t	p	Signif
(Intercept)	2.05854354	1.87735505	1.0965126	0.278003458	

Covariate	Beta	SE	t	p	Signif
GroupCI	1.18595585	0.35418176	3.3484385	0.001533434	**
SexMale	-0.22196897	0.34790576	-0.6380147	0.526317648	
Visit_GroupV2	-0.09758225	0.21990781	-0.4437416	0.659105755	
AgeAtVisit	0.03126848	0.02598647	1.2032599	0.234429744	
APOE4_INDEX	0.34915039	0.24227370	1.4411403	0.155654982	
Metabolic_Index	0.45471782	0.14039074	3.2389446	0.002112830	**

-----

43. Results for hsa-miR-148b-3p

Covariate	Beta	SE	t	p	Signif
(Intercept)	2.30346771	1.92475451	1.19675922	0.237215454	
GroupCI	1.19311100	0.36821407	3.24026451	0.002159622	**
SexMale	-0.13855742	0.35905410	-0.38589565	0.701260881	
Visit_GroupV2	0.01792474	0.24054624	0.07451681	0.940905553	
AgeAtVisit	0.03124861	0.02660247	1.17465056	0.245867505	
APOE4_INDEX	0.43547200	0.25103891	1.73467935	0.089146967	.
Metabolic_Index	0.25048083	0.14484465	1.72930678	0.090112582	.

-----

44. Results for hsa-miR-197-5p

Covariate	Beta	SE	t	p	Signif
(Intercept)	4.4733270118	1.88204923	2.37683847	0.021254201	*
GroupCI	1.0775307800	0.35642465	3.02316574	0.003908909	**
SexMale	-0.0662925288	0.34840967	-0.19027178	0.849851924	
Visit_GroupV2	0.1624627650	0.24701844	0.65769489	0.513693512	
AgeAtVisit	-0.0002779469	0.02593593	-0.01071667	0.991491329	
APOE4_INDEX	0.2510164436	0.24244276	1.03536372	0.305386247	
Metabolic_Index	0.3692326231	0.14060961	2.62594168	0.011380529	*

-----

45. Results for hsa-miR-4787-5p

Covariate	Beta	SE	t	p	Signif
(Intercept)	2.0492489	1.78399668	1.1486842	0.256270144	
GroupCI	1.1249629	0.34061970	3.3026947	0.001794496	**

Covariate	Beta	SE	t	p	Signif
SexMale	-0.4006062	0.33371831	-1.2004322	0.235750508	
Visit_GroupV2	0.1326939	0.24881339	0.5333068	0.596235211	
AgeAtVisit	0.0332005	0.02463762	1.3475527	0.184008492	
APOE4_INDEX	0.4503195	0.23303766	1.9323894	0.059110698	.
Metabolic_Index	0.4332305	0.13477893	3.2143782	0.002316169	**

-----

46. Results for hsa-miR-378d

Covariate	Beta	SE	t	p	Signif
(Intercept)	2.68592074	1.74904668	1.5356484	0.1308130535	
GroupCI	1.09342967	0.32940714	3.3193867	0.0016718285	**
SexMale	-0.37359985	0.32379765	-1.1538066	0.2539642873	
Visit_GroupV2	-0.06712047	0.23630924	-0.2840366	0.7775337001	
AgeAtVisit	0.02457373	0.02406361	1.0211988	0.3119857690	
APOE4_INDEX	0.28149649	0.22557588	1.2479016	0.2177730322	
Metabolic_Index	0.53356210	0.13117927	4.0674270	0.0001652623	***

-----

47. Results for hsa-miR-411-5p

Covariate	Beta	SE	t	p	Signif
(Intercept)	4.257092224	2.19924505	1.93570618	0.0583707531	.
GroupCI	1.453727303	0.41486568	3.50409154	0.0009539332	***
SexMale	-0.624640077	0.40798791	-1.53102594	0.1318482353	
Visit_GroupV2	0.004567805	0.22881837	0.01996258	0.9841499257	
AgeAtVisit	0.016854627	0.03043157	0.55385334	0.5820624964	
APOE4_INDEX	0.225698919	0.28244653	0.79908547	0.4278900511	
Metabolic_Index	0.044388943	0.16264480	0.27291953	0.7860002732	

-----

48. Results for hsa-miR-16-2-3p

Covariate	Beta	SE	t	p	Signif
(Intercept)	3.41808745	1.88602766	1.8123210	0.076268909	.
GroupCI	1.03043001	0.36194808	2.8469001	0.006504398	**
SexMale	0.03668633	0.34886735	0.1051584	0.916693557	
Visit_GroupV2	0.38829724	0.27503427	1.4118140	0.164531523	

Covariate	Beta	SE	t	p	Signif
AgeAtVisit	0.01897922	0.02594536	0.7315073	0.468068015	
APOE4_INDEX	0.23388608	0.24471820	0.9557364	0.344051287	
Metabolic_Index	0.34930254	0.14362191	2.4320978	0.018838318	*

-----

49. Results for hsa-miR-152-3p

Covariate	Beta	SE	t	p	Signif
(Intercept)	2.19834887	1.92215162	1.1436917	0.258052169	
GroupCI	1.23265468	0.36343829	3.3916478	0.001343669	**
SexMale	-0.52602442	0.35734375	-1.4720404	0.147109194	
Visit_GroupV2	-0.14617947	0.22333917	-0.6545178	0.515697582	
AgeAtVisit	0.03300815	0.02659106	1.2413249	0.220123137	
APOE4_INDEX	0.32321929	0.24809814	1.3027880	0.198456039	
Metabolic_Index	0.38940483	0.14341246	2.7152789	0.008996515	**

-----

50. Results for hsa-miR-4492

Covariate	Beta	SE	t	p	Signif
(Intercept)	2.63152102	1.81170376	1.4525118	0.1527333831	
GroupCI	1.08577964	0.34474566	3.1495092	0.0027855518	**
SexMale	-0.42877178	0.33728981	-1.2712266	0.2096496551	
Visit_GroupV2	-0.21190118	0.26306509	-0.8055086	0.4244180739	
AgeAtVisit	0.02860651	0.02494726	1.1466795	0.2570828522	
APOE4_INDEX	0.35974386	0.23562314	1.5267764	0.1332463855	
Metabolic_Index	0.49998346	0.13692981	3.6513851	0.0006333731	***

-----

51. Results for hsa-miR-423-3p

Covariate	Beta	SE	t	p	Signif
(Intercept)	3.64679289	2.05188897	1.7772857	0.081552678	.
GroupCI	1.24516196	0.38899724	3.2009532	0.002371673	**
SexMale	-0.39298887	0.38141825	-1.0303358	0.307769201	
Visit_GroupV2	-0.08298217	0.23735384	-0.3496138	0.728084519	
AgeAtVisit	0.01882285	0.02832437	0.6645461	0.509368561	
APOE4_INDEX	0.10101446	0.26456908	0.3818075	0.704208428	
Metabolic_Index	0.34319011	0.15372944	2.2324293	0.030056566	*

-----

52. Results for hsa-miR-6511b-3p

Covariate	Beta	SE	t	p	Signif
(Intercept)	3.59485798	1.78681135	2.0118845	0.049531556	*
GroupCI	1.02884937	0.33686973	3.0541461	0.003583557	**
SexMale	-0.19546468	0.33068544	-0.5910895	0.557072354	
Visit_GroupV2	0.26769807	0.22618350	1.1835438	0.242083448	
AgeAtVisit	0.01091131	0.02461242	0.4433255	0.659406140	
APOE4_INDEX	0.32518207	0.22996694	1.4140383	0.163428966	
Metabolic_Index	0.34245991	0.13300377	2.5748136	0.012975523	*

-----

53. Results for hsa-miR-454-3p

Covariate	Beta	SE	t	p	Signif
(Intercept)	3.0352504	2.04842878	1.4817456	0.144377213	
GroupCI	1.2365432	0.38776096	3.1889316	0.002407005	**
SexMale	-0.4216551	0.38054360	-1.1080336	0.272890768	
Visit_GroupV2	-0.2075841	0.21640132	-0.9592551	0.341821095	
AgeAtVisit	0.0229093	0.02834809	0.8081424	0.422650295	
APOE4_INDEX	0.2334464	0.26429443	0.8832815	0.381105405	
Metabolic_Index	0.3417782	0.15256957	2.2401466	0.029332922	*

-----

54. Results for hsa-miR-6741-5p

Covariate	Beta	SE	t	p	Signif
(Intercept)	3.22473730	1.73789833	1.8555385	0.069569188	.
GroupCI	1.00539839	0.33074900	3.0397625	0.003800988	**
SexMale	-0.17355183	0.32441738	-0.5349647	0.595105673	
Visit_GroupV2	0.20389926	0.23888706	0.8535383	0.397534575	
AgeAtVisit	0.01475344	0.02396961	0.6155061	0.541082928	
APOE4_INDEX	0.50182574	0.22585248	2.2219182	0.030961600	*
Metabolic_Index	0.36483954	0.13056910	2.7942257	0.007416352	**

-----

55. Results for hsa-let-7f-2-3p

Covariate	Beta	SE	t	p	Signif
(Intercept)	3.30995213	1.84533156	1.7936897	0.078750667	.
GroupCI	1.09131151	0.34976653	3.1201142	0.002963859	**
SexMale	-0.27403418	0.34353013	-0.7977006	0.428716970	
Visit_GroupV2	0.05349324	0.21482334	0.2490104	0.804345841	
AgeAtVisit	0.01587950	0.02550508	0.6226015	0.536301049	
APOE4_INDEX	0.29635470	0.23832845	1.2434718	0.219340039	
Metabolic_Index	0.38029071	0.13784325	2.7588634	0.008017426	**

-----

56. Results for hsa-miR-3615

Covariate	Beta	SE	t	p	Signif
(Intercept)	2.34434394	1.99721067	1.1738090	0.245900803	
GroupCI	1.16140973	0.37656623	3.0842110	0.003285729	**
SexMale	-0.35652880	0.37002979	-0.9635138	0.339816864	
Visit_GroupV2	-0.23810754	0.22309283	-1.0673025	0.290840150	
AgeAtVisit	0.03086121	0.02763069	1.1169179	0.269241546	
APOE4_INDEX	0.43081861	0.25698085	1.6764619	0.099741915	.
Metabolic_Index	0.36584938	0.14879122	2.4588103	0.017358568	*

-----

57. Results for hsa-miR-378c

Covariate	Beta	SE	t	p	Signif
(Intercept)	2.75693071	2.08282651	1.3236488	0.191408412	
GroupCI	1.24676696	0.39346625	3.1686758	0.002564610	**
SexMale	-0.07129282	0.38574553	-0.1848183	0.854090363	
Visit_GroupV2	0.03895087	0.20357384	0.1913353	0.849007866	
AgeAtVisit	0.02367370	0.02882495	0.8212922	0.415228629	
APOE4_INDEX	0.15199416	0.26824309	0.5666284	0.573403847	
Metabolic_Index	0.51253095	0.15541489	3.2978240	0.001761514	**

-----

58. Results for hsa-miR-6735-5p

Covariate	Beta	SE	t	p	Signif
(Intercept)	4.121482317	1.9249951	2.1410352	0.037148580	*

Covariate	Beta	SE	t	p	Signif
GroupCI	1.071376608	0.3643494	2.9405194	0.004943470	**
SexMale	-0.133150442	0.3566277	-0.3733598	0.710452230	
Visit_GroupV2	-0.093061810	0.2495170	-0.3729678	0.710742096	
AgeAtVisit	0.003938596	0.0265502	0.1483453	0.882664365	
APOE4_INDEX	0.337517400	0.2481963	1.3598807	0.179942097	
Metabolic_Index	0.472415973	0.1440878	3.2786667	0.001897812	**

-----

59. Results for hsa-miR-205-3p

Covariate	Beta	SE	t	p	Signif
(Intercept)	4.481192994	2.5845102	1.73386545	0.089070310	.
GroupCI	1.414897555	0.4896078	2.88985923	0.005675262	**
SexMale	0.059430954	0.4767883	0.12464851	0.901298836	
Visit_GroupV2	-0.022100368	0.3005169	-0.07354118	0.941667267	
AgeAtVisit	0.003233326	0.0357050	0.09055667	0.928204651	
APOE4_INDEX	0.183517831	0.3314420	0.55369524	0.582241484	
Metabolic_Index	0.411709311	0.1930806	2.13231785	0.037887730	*

-----

60. Results for hsa-miR-874-3p

Covariate	Beta	SE	t	p	Signif
(Intercept)	3.41099044	2.06376158	1.6528026	0.104593886	
GroupCI	1.19344963	0.39097967	3.0524595	0.003619185	**
SexMale	-0.27117816	0.38364368	-0.7068490	0.482918788	
Visit_GroupV2	-0.04442446	0.23934008	-0.1856123	0.853494537	
AgeAtVisit	0.01616764	0.02852651	0.5667586	0.573396138	
APOE4_INDEX	0.17193214	0.26648449	0.6451863	0.521735612	
Metabolic_Index	0.36345380	0.15402011	2.3597815	0.022201903	*

-----

61. Results for hsa-miR-125b-5p

Covariate	Beta	SE	t	p	Signif
(Intercept)	2.60218799	3.15919983	0.82368578	0.413925147	
GroupCI	1.85506164	0.59613207	3.11182997	0.003034042	**
SexMale	-0.04274994	0.58838289	-0.07265668	0.942361427	
Visit_GroupV2	-0.07865488	0.34095760	-0.23068817	0.818473156	

Covariate	Beta	SE	t	p	Signif
AgeAtVisit	0.03907126	0.04388423	0.89032561	0.377441057	
APOE4_INDEX	0.14686790	0.40911690	0.35898762	0.721075375	
Metabolic_Index	0.78148333	0.23701757	3.29715363	0.001776351	**

-----

62. Results for hsa-miR-222-3p

Covariate	Beta	SE	t	p	Signif
(Intercept)	3.63062786	2.06017107	1.7622944	0.083882034	.
GroupCI	1.19891368	0.38924115	3.0801309	0.003299225	**
SexMale	-0.46827767	0.38195701	-1.2259957	0.225709872	
Visit_GroupV2	-0.29344818	0.24255810	-1.2098057	0.231811151	
AgeAtVisit	0.01781388	0.02844634	0.6262275	0.533898356	
APOE4_INDEX	0.21488981	0.26562469	0.8089979	0.422193406	
Metabolic_Index	0.37029184	0.15362591	2.4103475	0.019499821	*

-----

63. Results for hsa-miR-140-5p

Covariate	Beta	SE	t	p	Signif
(Intercept)	3.42167514	2.0226781	1.6916558	0.096774976	.
GroupCI	1.13266992	0.3812327	2.9710721	0.004506644	**
SexMale	-0.39351465	0.3750500	-1.0492325	0.298986296	
Visit_GroupV2	-0.19934567	0.2426705	-0.8214664	0.415179910	
AgeAtVisit	0.01694502	0.0279199	0.6069152	0.546582473	
APOE4_INDEX	0.36422299	0.2605636	1.3978273	0.168176835	
Metabolic_Index	0.39800944	0.1504805	2.6449232	0.010815757	*

-----

64. Results for hsa-miR-6764-5p

Covariate	Beta	SE	t	p	Signif
(Intercept)	4.240727219	1.85947646	2.2806028	0.026719319	*
GroupCI	1.022411452	0.35030939	2.9185956	0.005193039	**
SexMale	-0.163313821	0.34284330	-0.4763512	0.635828894	
Visit_GroupV2	0.101832821	0.22687612	0.4488477	0.655415137	
AgeAtVisit	0.003806434	0.02562269	0.1485572	0.882480326	
APOE4_INDEX	0.367702943	0.23827613	1.5431799	0.128879418	
Metabolic_Index	0.298124060	0.13773877	2.1644165	0.035067505	*

-----

65. Results for hsa-miR-6512-5p

Covariate	Beta	SE	t	p	Signif
(Intercept)	4.120849629	1.98228494	2.0788382	0.042588006	*
GroupCI	1.126733218	0.37110747	3.0361373	0.003740917	**
SexMale	-0.451359503	0.36531096	-1.2355487	0.222185442	
Visit_GroupV2	0.243985217	0.25443868	0.9589156	0.342043675	
AgeAtVisit	0.005489233	0.02728113	0.2012099	0.841320312	
APOE4_INDEX	0.225164779	0.25384971	0.8870003	0.379167366	
Metabolic_Index	0.439426973	0.14708653	2.9875406	0.004285756	**

-----

66. Results for hsa-miR-1250-5p

Covariate	Beta	SE	t	p	Signif
(Intercept)	3.852999932	1.93691879	1.9892419	0.051836892	.
GroupCI	1.147248374	0.36357401	3.1554741	0.002639054	**
SexMale	-0.423837094	0.35824746	-1.1830847	0.242045607	
Visit_GroupV2	0.105188534	0.22607556	0.4652804	0.643633272	
AgeAtVisit	0.009355362	0.02670033	0.3503837	0.727436912	
APOE4_INDEX	0.195735544	0.24843880	0.7878622	0.434280333	
Metabolic_Index	0.374306563	0.14364589	2.6057589	0.011868802	*

-----

67. Results for hsa-miR-369-3p

Covariate	Beta	SE	t	p	Signif
(Intercept)	2.64219643	1.98675779	1.3299036	0.189280005	
GroupCI	1.20781707	0.37636264	3.2091843	0.002267735	**
SexMale	-0.47335856	0.36947400	-1.2811688	0.205745918	
Visit_GroupV2	-0.04116867	0.21580678	-0.1907664	0.849441989	
AgeAtVisit	0.02890335	0.02750667	1.0507762	0.298158202	
APOE4_INDEX	0.22590125	0.25643216	0.8809396	0.382352312	
Metabolic_Index	0.32986748	0.14795224	2.2295538	0.030064207	*

-----

68. Results for hsa-miR-22-5p

Covariate	Beta	SE	t	p	Signif
(Intercept)	2.01767246	1.90428579	1.0595429	0.294339932	
GroupCI	1.15611105	0.36222953	3.1916532	0.002421537	**
SexMale	-0.50953705	0.35465845	-1.4366979	0.156904229	
Visit_GroupV2	-0.23718978	0.24424633	-0.9711089	0.336072739	
AgeAtVisit	0.03792207	0.02630587	1.4415822	0.155526622	
APOE4_INDEX	0.24226737	0.24654040	0.9826680	0.330405059	
Metabolic_Index	0.46889709	0.14361851	3.2648793	0.001958667	**

-----

69. Results for hsa-miR-1255b-5p

Covariate	Beta	SE	t	p	Signif
(Intercept)	1.89422947	1.75529152	1.0791538	0.285731785	
GroupCI	1.04933501	0.33131066	3.1672238	0.002631482	**
SexMale	-0.28525708	0.32591641	-0.8752461	0.385652814	
Visit_GroupV2	-0.18498005	0.22839344	-0.8099184	0.421850345	
AgeAtVisit	0.03661794	0.02425437	1.5097460	0.137443879	
APOE4_INDEX	0.38289911	0.22684475	1.6879346	0.097694067	.
Metabolic_Index	0.47224091	0.13123493	3.5984390	0.000736915	***

-----

70. Results for hsa-miR-518e-5p

Covariate	Beta	SE	t	p	Signif
(Intercept)	2.21332049	1.6618554	1.3318370	0.189323973	
GroupCI	0.97467661	0.3175684	3.0691861	0.003555617	**
SexMale	-0.27566066	0.3104648	-0.8878966	0.379108848	
Visit_GroupV2	-0.20036221	0.2540090	-0.7887996	0.434182265	
AgeAtVisit	0.03299129	0.0228851	1.4416056	0.156030759	
APOE4_INDEX	0.46816515	0.2167335	2.1600959	0.035886948	*
Metabolic_Index	0.38181757	0.1256041	3.0398495	0.003857250	**

-----

71. Results for hsa-miR-519b-5p

Covariate	Beta	SE	t	p	Signif
(Intercept)	2.21332049	1.6618554	1.3318370	0.189323973	

Covariate	Beta	SE	t	p	Signif
GroupCI	0.97467661	0.3175684	3.0691861	0.003555617	**
SexMale	-0.27566066	0.3104648	-0.8878966	0.379108848	
Visit_GroupV2	-0.20036221	0.2540090	-0.7887996	0.434182265	
AgeAtVisit	0.03299129	0.0228851	1.4416056	0.156030759	
APOE4_INDEX	0.46816515	0.2167335	2.1600959	0.035886948	*
Metabolic_Index	0.38181757	0.1256041	3.0398495	0.003857250	**

-----

72. Results for hsa-miR-519c-5p

Covariate	Beta	SE	t	p	Signif
(Intercept)	2.21332049	1.6618554	1.3318370	0.189323973	
GroupCI	0.97467661	0.3175684	3.0691861	0.003555617	**
SexMale	-0.27566066	0.3104648	-0.8878966	0.379108848	
Visit_GroupV2	-0.20036221	0.2540090	-0.7887996	0.434182265	
AgeAtVisit	0.03299129	0.0228851	1.4416056	0.156030759	
APOE4_INDEX	0.46816515	0.2167335	2.1600959	0.035886948	*
Metabolic_Index	0.38181757	0.1256041	3.0398495	0.003857250	**

-----

73. Results for hsa-miR-522-5p

Covariate	Beta	SE	t	p	Signif
(Intercept)	2.21332049	1.6618554	1.3318370	0.189323973	
GroupCI	0.97467661	0.3175684	3.0691861	0.003555617	**
SexMale	-0.27566066	0.3104648	-0.8878966	0.379108848	
Visit_GroupV2	-0.20036221	0.2540090	-0.7887996	0.434182265	
AgeAtVisit	0.03299129	0.0228851	1.4416056	0.156030759	
APOE4_INDEX	0.46816515	0.2167335	2.1600959	0.035886948	*
Metabolic_Index	0.38181757	0.1256041	3.0398495	0.003857250	**

-----

74. Results for hsa-miR-523-5p

Covariate	Beta	SE	t	p	Signif
(Intercept)	2.21332049	1.6618554	1.3318370	0.189323973	
GroupCI	0.97467661	0.3175684	3.0691861	0.003555617	**
SexMale	-0.27566066	0.3104648	-0.8878966	0.379108848	
Visit_GroupV2	-0.20036221	0.2540090	-0.7887996	0.434182265	

Covariate	Beta	SE	t	p	Signif
AgeAtVisit	0.03299129	0.0228851	1.4416056	0.156030759	
APOE4_INDEX	0.46816515	0.2167335	2.1600959	0.035886948	*
Metabolic_Index	0.38181757	0.1256041	3.0398495	0.003857250	**

-----

75. Results for hsa-miR-340-5p

Covariate	Beta	SE	t	p	Signif
(Intercept)	1.47830353	2.06636169	0.7154137	0.478198943	
GroupCI	1.26344076	0.40077971	3.1524569	0.002939955	**
SexMale	0.27539375	0.38658126	0.7123826	0.480054491	
Visit_GroupV2	0.20826277	0.30198278	0.6896511	0.494098395	
AgeAtVisit	0.04987537	0.02853626	1.7477896	0.087598993	.
APOE4_INDEX	0.32835147	0.26965807	1.2176586	0.229957161	
Metabolic_Index	0.25137713	0.15867916	1.5841849	0.120439172	

-----

76. Results for hsa-miR-4674

Covariate	Beta	SE	t	p	Signif
(Intercept)	1.90829534	3.48045285	0.5482894	0.585850378	
GroupCI	2.26434925	0.65264924	3.4694735	0.001059607	**
SexMale	-1.09748085	0.64809299	-1.6934003	0.096390193	.
Visit_GroupV2	-0.47797634	0.33721460	-1.4174248	0.162350619	
AgeAtVisit	0.06508906	0.04828521	1.3480123	0.183524623	
APOE4_INDEX	0.10172554	0.45144339	0.2253340	0.822606787	
Metabolic_Index	0.80759408	0.25711904	3.1409345	0.002783236	**

-----

77. Results for hsa-miR-548c-5p

Covariate	Beta	SE	t	p	Signif
(Intercept)	4.025281473	1.8803252	2.1407368	0.0369944593	*
GroupCI	1.029175734	0.3543070	2.9047572	0.0053815776	**
SexMale	-0.293620375	0.3483390	-0.8429156	0.4031284056	
Visit_GroupV2	-0.251256845	0.2127252	-1.1811332	0.2429130059	
AgeAtVisit	0.008397373	0.0259611	0.3234599	0.7476416301	
APOE4_INDEX	0.316135792	0.2426537	1.3028270	0.1983623359	
Metabolic_Index	0.494048432	0.1402195	3.5233929	0.0008961962	***

-----

78. Results for hsa-miR-548o-5p

Covariate	Beta	SE	t	p	Signif
(Intercept)	4.025281473	1.8803252	2.1407368	0.0369944593	*
GroupCI	1.029175734	0.3543070	2.9047572	0.0053815776	**
SexMale	-0.293620375	0.3483390	-0.8429156	0.4031284056	
Visit_GroupV2	-0.251256845	0.2127252	-1.1811332	0.2429130059	
AgeAtVisit	0.008397373	0.0259611	0.3234599	0.7476416301	
APOE4_INDEX	0.316135792	0.2426537	1.3028270	0.1983623359	
Metabolic_Index	0.494048432	0.1402195	3.5233929	0.0008961962	***

-----

79. Results for hsa-miR-93-3p

Covariate	Beta	SE	t	p	Signif
(Intercept)	3.00507554	1.72923427	1.7378071	0.088613162	.
GroupCI	0.98990302	0.32973129	3.0021507	0.004234331	**
SexMale	-0.36585610	0.32192050	-1.1364797	0.261359340	
Visit_GroupV2	-0.15629627	0.25584624	-0.6108992	0.544128635	
AgeAtVisit	0.02630572	0.02373539	1.1082912	0.273220716	
APOE4_INDEX	0.33110169	0.22387570	1.4789533	0.145641051	
Metabolic_Index	0.33613097	0.13040705	2.5775521	0.013054247	*

-----

80. Results for hsa-miR-3125

Covariate	Beta	SE	t	p	Signif
(Intercept)	4.372385247	2.03497877	2.14861467	0.036341813	*
GroupCI	1.111891961	0.37999575	2.92606420	0.005078959	**
SexMale	-0.434201617	0.37505819	-1.15769133	0.252279452	
Visit_GroupV2	-0.085319498	0.24078137	-0.35434426	0.724513698	
AgeAtVisit	0.001457327	0.02802461	0.05200169	0.958726578	
APOE4_INDEX	0.381330519	0.26041152	1.46433811	0.149120775	
Metabolic_Index	0.450494146	0.15022553	2.99878555	0.004151535	**

-----

81. Results for hsa-miR-4659b-5p

Covariate	Beta	SE	t	p	Signif
(Intercept)	2.82273055	2.02306978	1.3952710	0.168838603	
GroupCI	1.16829091	0.38221465	3.0566356	0.003523101	**
SexMale	-0.37026891	0.37525833	-0.9867040	0.328336626	
Visit_GroupV2	-0.22034831	0.21524394	-1.0237143	0.310688257	
AgeAtVisit	0.02383867	0.02802052	0.8507574	0.398787538	
APOE4_INDEX	0.23575680	0.26057092	0.9047702	0.369741634	
Metabolic_Index	0.43509013	0.15065566	2.8879774	0.005630014	**

-----

82. Results for hsa-miR-6777-5p

Covariate	Beta	SE	t	p	Signif
(Intercept)	4.3281610856	2.10556064	2.05558606	0.044852025	*
GroupCI	1.0897372042	0.39563173	2.75442319	0.008079654	**
SexMale	-0.1717940177	0.38893123	-0.44170795	0.660529281	
Visit_GroupV2	-0.2263798473	0.24729475	-0.91542521	0.364187877	
AgeAtVisit	0.0004709261	0.02910611	0.01617963	0.987152915	
APOE4_INDEX	0.3709148073	0.27201198	1.36359734	0.178559937	
Metabolic_Index	0.4858805427	0.15653593	3.10395534	0.003084667	**

-----

83. Results for hsa-miR-6813-5p

Covariate	Beta	SE	t	p	Signif
(Intercept)	2.81176606	1.94008105	1.4493034	0.153239331	
GroupCI	1.10217001	0.36576399	3.0133366	0.003981547	**
SexMale	-0.24324222	0.35971684	-0.6762047	0.501898402	
Visit_GroupV2	0.11489750	0.21470913	0.5351309	0.594833708	
AgeAtVisit	0.02100822	0.02685911	0.7821638	0.437654984	
APOE4_INDEX	0.30397549	0.24985250	1.2166198	0.229225972	
Metabolic_Index	0.38323437	0.14437004	2.6545283	0.010505660	*

-----

84. Results for hsa-miR-502-3p

Covariate	Beta	SE	t	p	Signif
(Intercept)	2.180911254	2.00978522	1.08514643	0.283008861	
GroupCI	1.108512755	0.37952225	2.92081098	0.005206902	**
SexMale	-0.109293874	0.37255772	-0.29336092	0.770448237	
Visit_GroupV2	-0.005274098	0.23596815	-0.02235089	0.982256119	
AgeAtVisit	0.030632272	0.02781763	1.10118192	0.276037365	
APOE4_INDEX	0.279853528	0.25917512	1.07978546	0.285366742	
Metabolic_Index	0.408057918	0.15037165	2.71366253	0.009078584	**

-----

85. Results for hsa-miR-1292-5p

Covariate	Beta	SE	t	p	Signif
(Intercept)	5.268781247	1.8851527	2.7948831	0.007436907	**
GroupCI	0.983751768	0.3580276	2.7476979	0.008423932	**
SexMale	-0.472363165	0.3485125	-1.3553694	0.181632319	
Visit_GroupV2	-0.258011500	0.2850090	-0.9052751	0.369833259	
AgeAtVisit	-0.002919245	0.0257287	-0.1134626	0.910136226	
APOE4_INDEX	0.349832285	0.2438143	1.4348312	0.157807493	
Metabolic_Index	0.450437501	0.1419606	3.1729745	0.002629685	**

-----

86. Results for hsa-let-7c-3p

Covariate	Beta	SE	t	p	Signif
(Intercept)	2.67026189	1.98785375	1.3432889	0.185020254	
GroupCI	1.14417273	0.37338301	3.0643406	0.003455654	**
SexMale	-0.35515739	0.36781203	-0.9655948	0.338722871	
Visit_GroupV2	-0.09528967	0.22592125	-0.4217827	0.674924830	
AgeAtVisit	0.02327344	0.02753387	0.8452659	0.401840647	
APOE4_INDEX	0.27997791	0.25547091	1.0959288	0.278167108	
Metabolic_Index	0.51083987	0.14785567	3.4549900	0.001104701	**

-----

87. Results for hsa-miR-374a-5p

Covariate	Beta	SE	t	p	Signif
(Intercept)	3.10826336	2.04753010	1.5180550	0.135005201	
GroupCI	1.14216231	0.38669442	2.9536561	0.004691724	**
SexMale	-0.41883370	0.38009900	-1.1019069	0.275532719	

Covariate	Beta	SE	t	p	Signif
Visit_GroupV2	-0.26788708	0.23256398	-1.1518855	0.254591357	
AgeAtVisit	0.02170649	0.02830522	0.7668723	0.446595442	
APOE4_INDEX	0.31734396	0.26410613	1.2015774	0.234924526	
Metabolic_Index	0.38015030	0.15232049	2.4957265	0.015751381	*

-----

88. Results for hsa-miR-6876-5p

Covariate	Beta	SE	t	p	Signif
(Intercept)	3.34343861	1.88196462	1.7765683	0.081453340	.
GroupCI	1.06424273	0.35513027	2.9967672	0.004165269	**
SexMale	-0.32566923	0.34953031	-0.9317339	0.355753586	
Visit_GroupV2	0.11746458	0.20641471	0.5690708	0.571742992	
AgeAtVisit	0.01514135	0.02602758	0.5817425	0.563238274	
APOE4_INDEX	0.30511287	0.24252828	1.2580507	0.213958866	
Metabolic_Index	0.36791248	0.14007504	2.6265386	0.011288549	*

-----

89. Results for hsa-miR-486-5p

Covariate	Beta	SE	t	p	Signif
(Intercept)	13.40232824	1.18386990	11.3207779	3.954010e-15	***
GroupCI	-0.82496532	0.22079067	-3.7364138	4.977486e-04	***
SexMale	0.38268377	0.22419285	1.7069401	9.432080e-02	.
Visit_GroupV2	-1.05871067	0.20433288	-5.1813035	4.374085e-06	***
AgeAtVisit	0.01131856	0.01685083	0.6716914	5.050129e-01	
APOE4_INDEX	0.11679908	0.16309758	0.7161301	4.773944e-01	
Metabolic_Index	-0.10413055	0.08831188	-1.1791228	2.441838e-01	

-----

90. Results for hsa-miR-17-3p

Covariate	Beta	SE	t	p	Signif
(Intercept)	3.20624295	2.01559034	1.5907215	0.117778297	
GroupCI	1.17495690	0.38147099	3.0800688	0.003313409	**
SexMale	-0.43612089	0.37326504	-1.1683947	0.248015196	
Visit_GroupV2	-0.13205696	0.20933100	-0.6308524	0.530920255	
AgeAtVisit	0.02548695	0.02788071	0.9141428	0.364891942	
APOE4_INDEX	0.18881865	0.25888919	0.7293416	0.469089145	

Covariate	Beta	SE	t	p	Signif
Metabolic_Index	0.23958983	0.15003933	1.5968469	0.116403955	

-----

91. Results for hsa-miR-3605-3p

Covariate	Beta	SE	t	p	Signif
(Intercept)	3.10779128	1.94475867	1.5980344	0.116287421	
GroupCI	1.05994777	0.36730840	2.8857161	0.005735077	**
SexMale	-0.25242456	0.35952691	-0.7021020	0.485845895	
Visit_GroupV2	-0.25257186	0.23216985	-1.0878754	0.281825839	
AgeAtVisit	0.02085998	0.02683719	0.7772788	0.440627951	
APOE4_INDEX	0.30805638	0.24977460	1.2333375	0.223171838	
Metabolic_Index	0.38985472	0.14515300	2.6858192	0.009771753	**

-----

92. Results for hsa-miR-485-5p

Covariate	Beta	SE	t	p	Signif
(Intercept)	5.200218908	1.76773523	2.9417408	0.004916555	**
GroupCI	0.931637246	0.33593864	2.7732364	0.007756067	**
SexMale	-0.389039079	0.32790057	-1.1864544	0.240994509	
Visit_GroupV2	0.549368142	0.26546016	2.0694938	0.043634718	*
AgeAtVisit	-0.003566452	0.02417057	-0.1475535	0.883282701	
APOE4_INDEX	0.445119976	0.22901058	1.9436655	0.057518255	.
Metabolic_Index	0.158612116	0.13177966	1.2036160	0.234349591	

-----

93. Results for hsa-miR-548am-5p

Covariate	Beta	SE	t	p	Signif
(Intercept)	3.921400988	1.82464582	2.1491300	0.0363405723	*
GroupCI	0.950940886	0.34351135	2.7682954	0.0078100405	**
SexMale	-0.273527469	0.33758166	-0.8102557	0.4215178442	
Visit_GroupV2	-0.244001035	0.22407285	-1.0889362	0.2812464502	
AgeAtVisit	0.009123268	0.02514791	0.3627843	0.7182492388	
APOE4_INDEX	0.358791791	0.23528487	1.5249251	0.1333896936	
Metabolic_Index	0.523230825	0.13632389	3.8381449	0.0003401837	***

-----

94. Results for hsa-miR-3659

Covariate	Beta	SE	t	p	Signif
(Intercept)	2.03726370	1.84587395	1.1036852	0.275233353	
GroupCI	1.07340270	0.35090794	3.0589296	0.003628180	**
SexMale	-0.33926751	0.34422608	-0.9855950	0.329275145	
Visit_GroupV2	0.20507520	0.26061081	0.7869021	0.435208147	
AgeAtVisit	0.03166322	0.02548919	1.2422214	0.220191242	
APOE4_INDEX	0.42526822	0.24191948	1.7578916	0.085142888	.
Metabolic_Index	0.43530177	0.13898715	3.1319568	0.002956393	**

-----

95. Results for hsa-miR-1185-1-3p

Covariate	Beta	SE	t	p	Signif
(Intercept)	3.33468368	1.98241131	1.6821351	0.098495426	.
GroupCI	1.11764748	0.37587319	2.9734695	0.004442789	**
SexMale	-0.30907450	0.36870336	-0.8382742	0.405685642	
Visit_GroupV2	0.03433558	0.20597552	0.1666974	0.868249979	
AgeAtVisit	0.01977867	0.02743309	0.7209786	0.474128258	
APOE4_INDEX	0.23382007	0.25580802	0.9140451	0.364881387	
Metabolic_Index	0.25918530	0.14769768	1.7548367	0.085134730	.

-----

96. Results for hsa-miR-148a-3p

Covariate	Beta	SE	t	p	Signif
(Intercept)	3.57688230	1.97459632	1.8114499	0.075897586	.
GroupCI	1.01634995	0.37184418	2.7332684	0.008570674	**
SexMale	-0.22604838	0.36424643	-0.6205919	0.537601707	
Visit_GroupV2	0.07741758	0.25527163	0.3032753	0.762900366	
AgeAtVisit	0.01389020	0.02722038	0.5102868	0.612028205	
APOE4_INDEX	0.38311107	0.25458759	1.5048301	0.138469198	
Metabolic_Index	0.42178132	0.14676725	2.8738109	0.005877493	**

-----

97. Results for hsa-miR-199b-5p

Covariate	Beta	SE	t	p	Signif
(Intercept)	3.00027623	1.95225873	1.5368231	0.130335257	

Covariate	Beta	SE	t	p	Signif
GroupCI	1.10360427	0.36803904	2.9986065	0.004136735	**
SexMale	-0.46073475	0.36232198	-1.2716169	0.209107420	
Visit_GroupV2	-0.10426582	0.20893621	-0.4990318	0.619839279	
AgeAtVisit	0.02090537	0.02701188	0.7739325	0.442437976	
APOE4_INDEX	0.26298716	0.25138222	1.0461645	0.300272462	
Metabolic_Index	0.44668105	0.14535232	3.0730921	0.003354199	**

-----

98. Results for hsa-miR-3617-5p

Covariate	Beta	SE	t	p	Signif
(Intercept)	3.42691595	1.97796735	1.7325442	0.089062802	.
GroupCI	1.06739639	0.36996095	2.8851596	0.005668510	**
SexMale	-0.41721270	0.36428158	-1.1453027	0.257290844	
Visit_GroupV2	-0.06312862	0.22807741	-0.2767860	0.783034014	
AgeAtVisit	0.01429433	0.02730455	0.5235144	0.602824724	
APOE4_INDEX	0.37989369	0.25278894	1.5028098	0.138894761	
Metabolic_Index	0.42894380	0.14598372	2.9382990	0.004898213	**

-----

99. Results for hsa-miR-1268a

Covariate	Beta	SE	t	p	Signif
(Intercept)	0.74814537	1.84245948	0.4060580	0.686535056	
GroupCI	1.08204973	0.35130714	3.0800676	0.003445921	**
SexMale	-0.08017699	0.34232285	-0.2342146	0.815831383	
Visit_GroupV2	-0.29216028	0.24536471	-1.1907184	0.239717993	
AgeAtVisit	0.05360110	0.02551513	2.1007570	0.041030098	*
APOE4_INDEX	0.32488477	0.23843899	1.3625489	0.179490922	
Metabolic_Index	0.44002649	0.13931975	3.1583928	0.002765771	**

-----

100. Results for hsa-miR-31-5p

Covariate	Beta	SE	t	p	Signif
(Intercept)	3.75031114	2.83519385	1.3227706	0.1920496743	
GroupCI	1.57630176	0.53277333	2.9586724	0.0047462761	**
SexMale	-0.85929189	0.53242475	-1.6139218	0.1129641692	
Visit_GroupV2	-0.42893309	0.33056662	-1.2975693	0.2005086672	

Covariate	Beta	SE	t	p	Signif
AgeAtVisit	0.01843056	0.03932729	0.4686457	0.6414004034	
APOE4_INDEX	0.55018614	0.37284106	1.4756587	0.1464305274	
Metabolic_Index	0.75049320	0.21185403	3.5425014	0.0008810796	***

-----

101. Results for hsa-miR-338-3p

Covariate	Beta	SE	t	p	Signif
(Intercept)	3.3958542	2.00473311	1.6939184	0.096480042	.
GroupCI	1.0379272	0.37808947	2.7451893	0.008370960	**
SexMale	-0.3049100	0.37089619	-0.8220900	0.414914951	
Visit_GroupV2	-0.1125914	0.25376864	-0.4436772	0.659183277	
AgeAtVisit	0.0160189	0.02765223	0.5792987	0.564979325	
APOE4_INDEX	0.2398991	0.25779368	0.9305858	0.356523426	
Metabolic_Index	0.4871186	0.14991654	3.2492653	0.002067847	**

-----

102. Results for hsa-miR-3187-3p

Covariate	Beta	SE	t	p	Signif
(Intercept)	3.747267633	1.84717597	2.0286468	0.047875670	*
GroupCI	0.912873224	0.35076092	2.6025511	0.012166776	*
SexMale	-0.257939787	0.34394686	-0.7499408	0.456829977	
Visit_GroupV2	0.043579108	0.25105547	0.1735836	0.862898628	
AgeAtVisit	0.008929298	0.02545016	0.3508542	0.727182307	
APOE4_INDEX	0.482581172	0.24139477	1.9991368	0.051083018	.
Metabolic_Index	0.429675699	0.13865569	3.0988681	0.003195713	**

-----

103. Results for hsa-miR-671-5p

Covariate	Beta	SE	t	p	Signif
(Intercept)	1.70695648	2.06758676	0.8255791	0.412761603	
GroupCI	1.13251013	0.39075547	2.8982579	0.005455571	**
SexMale	-0.16086269	0.38326500	-0.4197166	0.676397265	
Visit_GroupV2	0.07468868	0.22931974	0.3256967	0.745942484	
AgeAtVisit	0.03798739	0.02866885	1.3250405	0.190869659	
APOE4_INDEX	0.19651632	0.26638239	0.7377226	0.463952664	
Metabolic_Index	0.43315557	0.15434849	2.8063480	0.007004450	**

-----

104. Results for hsa-miR-337-3p

Covariate	Beta	SE	t	p	Signif
(Intercept)	3.12109182	1.97716928	1.5785658	0.120442159	
GroupCI	1.07495113	0.37212110	2.8887132	0.005608784	**
SexMale	-0.31904065	0.36653632	-0.8704203	0.388033037	
Visit_GroupV2	0.14639932	0.20839360	0.7025135	0.485462061	
AgeAtVisit	0.01821863	0.02736335	0.6658043	0.508450913	
APOE4_INDEX	0.28583902	0.25421125	1.1244153	0.265951285	
Metabolic_Index	0.35993671	0.14682794	2.4514184	0.017592412	*

-----

105. Results for hsa-miR-1301-3p

Covariate	Beta	SE	t	p	Signif
(Intercept)	4.479633270	1.64796089	2.71828858	0.009022369	**
GroupCI	0.787669359	0.31645076	2.48907402	0.016217459	*
SexMale	0.015153254	0.30695943	0.04936566	0.960826621	
Visit_GroupV2	0.217993841	0.25071569	0.86948623	0.388779421	
AgeAtVisit	0.006085079	0.02253322	0.27004927	0.788245452	
APOE4_INDEX	0.504379082	0.21496791	2.34629943	0.023008703	*
Metabolic_Index	0.173430563	0.12440430	1.39408821	0.169519845	

-----

106. Results for hsa-miR-6780a-5p

Covariate	Beta	SE	t	p	Signif
(Intercept)	3.2597283	1.99972221	1.6300906	0.109107324	
GroupCI	1.0701910	0.37699432	2.8387456	0.006440102	**
SexMale	-0.3895013	0.37129769	-1.0490269	0.299002276	
Visit_GroupV2	0.1105618	0.21750643	0.5083152	0.613375555	
AgeAtVisit	0.0170347	0.02767051	0.6156264	0.540818281	
APOE4_INDEX	0.3271542	0.25750041	1.2704998	0.209544585	
Metabolic_Index	0.3674823	0.14866666	2.4718540	0.016741421	*

-----

107. Results for hsa-miR-6512-3p

Covariate	Beta	SE	t	p	Signif
(Intercept)	2.64713903	2.06177232	1.2839143	0.204939426	
GroupCI	1.05418551	0.38996417	2.7032881	0.009288276	**
SexMale	-0.21129489	0.38218572	-0.5528592	0.582760550	
Visit_GroupV2	-0.08812506	0.25574850	-0.3445770	0.731820714	
AgeAtVisit	0.02482167	0.02849519	0.8710831	0.387766200	
APOE4_INDEX	0.36365074	0.26577622	1.3682591	0.177192848	
Metabolic_Index	0.42294931	0.15412249	2.7442413	0.008337053	**

-----

108. Results for hsa-miR-4433b-5p

Covariate	Beta	SE	t	p	Signif
(Intercept)	3.72990671	1.72018780	2.1683137	0.035066722	*
GroupCI	0.93026220	0.33111139	2.8095143	0.007131557	**
SexMale	-0.23597119	0.32027065	-0.7367868	0.464799030	
Visit_GroupV2	0.43310271	0.26567288	1.6302105	0.109531168	
AgeAtVisit	0.02318705	0.02353392	0.9852608	0.329382707	
APOE4_INDEX	0.35589683	0.22364497	1.5913474	0.118025057	
Metabolic_Index	0.08857460	0.13000508	0.6813165	0.498909640	

-----

109. Results for hsa-miR-550a-5p

Covariate	Beta	SE	t	p	Signif
(Intercept)	3.09326651	1.78066115	1.73714495	0.088597204	.
GroupCI	0.92914173	0.34110557	2.72391247	0.008896045	**
SexMale	0.11114135	0.33119602	0.33557572	0.738612686	
Visit_GroupV2	-0.02152505	0.24308152	-0.08855074	0.929797178	
AgeAtVisit	0.02410146	0.02452812	0.98260518	0.330592642	
APOE4_INDEX	0.24451496	0.23003022	1.06296887	0.292967779	
Metabolic_Index	0.27264358	0.13421295	2.03142537	0.047612594	*

-----

110. Results for hsa-miR-550a-3-5p

Covariate	Beta	SE	t	p	Signif
(Intercept)	3.09326651	1.78066115	1.73714495	0.088597204	.

Covariate	Beta	SE	t	p	Signif
GroupCI	0.92914173	0.34110557	2.72391247	0.008896045	**
SexMale	0.11114135	0.33119602	0.33557572	0.738612686	
Visit_GroupV2	-0.02152505	0.24308152	-0.08855074	0.929797178	
AgeAtVisit	0.02410146	0.02452812	0.98260518	0.330592642	
APOE4_INDEX	0.24451496	0.23003022	1.06296887	0.292967779	
Metabolic_Index	0.27264358	0.13421295	2.03142537	0.047612594	*

-----

111. Results for hsa-miR-106b-5p

Covariate	Beta	SE	t	p	Signif
(Intercept)	3.27698397	1.95698409	1.6745072	0.100102895	
GroupCI	0.99959707	0.37031508	2.6993150	0.009379914	**
SexMale	-0.32015524	0.36396276	-0.8796374	0.383147900	
Visit_GroupV2	-0.24044955	0.22266184	-1.0798867	0.285231408	
AgeAtVisit	0.02228348	0.02705666	0.8235858	0.413978206	
APOE4_INDEX	0.38759652	0.25291864	1.5324949	0.131529734	
Metabolic_Index	0.32844777	0.14558187	2.2561035	0.028344456	*

-----

112. Results for hsa-miR-345-5p

Covariate	Beta	SE	t	p	Signif
(Intercept)	4.199348557	2.05457735	2.04389898	0.045889555	*
GroupCI	1.074897252	0.38565224	2.78721903	0.007338348	**
SexMale	-0.347075354	0.37664279	-0.92149742	0.360919177	
Visit_GroupV2	-0.019801222	0.23914942	-0.08279854	0.934320110	
AgeAtVisit	0.007862688	0.02829719	0.27786106	0.782187799	
APOE4_INDEX	0.060139773	0.26115190	0.23028656	0.818745586	
Metabolic_Index	0.513539387	0.15336297	3.34852260	0.001491933	**

-----

113. Results for hsa-miR-1270

Covariate	Beta	SE	t	p	Signif
(Intercept)	4.366494135	1.96917459	2.21742356	0.03140294	*
GroupCI	0.931389017	0.37718027	2.46934710	0.01717629	*
SexMale	0.032024070	0.36515262	0.08770051	0.93048258	
Visit_GroupV2	-0.505688931	0.27811093	-1.81829939	0.07531289	.

Covariate	Beta	SE	t	p	Signif
AgeAtVisit	0.009302916	0.02698822	0.34470281	0.73183602	
APOE4_INDEX	0.419256913	0.25591540	1.63826368	0.10796015	
Metabolic_Index	0.308710038	0.14818172	2.08332072	0.04261618	*

-----

114. Results for hsa-miR-6873-3p

Covariate	Beta	SE	t	p	Signif
(Intercept)	2.14855683	2.19284415	0.9798037	0.331788964	
GroupCI	1.14268963	0.41310142	2.7661237	0.007870814	**
SexMale	-0.39629417	0.40788582	-0.9715811	0.335825492	
Visit_GroupV2	-0.04240723	0.23086503	-0.1836884	0.854983243	
AgeAtVisit	0.02983064	0.03039157	0.9815432	0.330939179	
APOE4_INDEX	0.48093293	0.28289657	1.7000310	0.095191393	.
Metabolic_Index	0.46536132	0.16293332	2.8561457	0.006180403	**

-----

115. Results for hsa-miR-548au-5p

Covariate	Beta	SE	t	p	Signif
(Intercept)	3.33485335	2.01226279	1.6572653	0.103394306	
GroupCI	1.05489095	0.37700858	2.7980555	0.007159288	**
SexMale	-0.42800381	0.37203602	-1.1504365	0.255145740	
Visit_GroupV2	-0.18447457	0.20707802	-0.8908457	0.377051785	
AgeAtVisit	0.01518625	0.02786056	0.5450807	0.587992221	
APOE4_INDEX	0.33755034	0.25864361	1.3050790	0.197522671	
Metabolic_Index	0.51162138	0.14932296	3.4262741	0.001192086	**

-----

116. Results for hsa-miR-30c-1-3p

Covariate	Beta	SE	t	p	Signif
(Intercept)	3.25953368	2.00061461	1.6292662	0.10957324	
GroupCI	1.18836018	0.37838968	3.1405724	0.00283692	**
SexMale	-0.83713433	0.37315838	-2.2433754	0.02935647	*
Visit_GroupV2	0.04727333	0.24096490	0.1961835	0.84526573	
AgeAtVisit	0.02745366	0.02763283	0.9935161	0.32526635	
APOE4_INDEX	0.19343900	0.25972084	0.7447958	0.45989918	
Metabolic_Index	0.27822607	0.14931910	1.8632986	0.06832854	.

-----

117. Results for hsa-miR-641

Covariate	Beta	SE	t	p	Signif
(Intercept)	3.97563178	1.86919283	2.1269244	0.038154621	*
GroupCI	0.99652536	0.34886318	2.8564933	0.006128695	**
SexMale	-0.62091484	0.34433075	-1.8032512	0.077101233	.
Visit_GroupV2	0.05877561	0.24889005	0.2361509	0.814236478	
AgeAtVisit	0.01225466	0.02565095	0.4777468	0.634817895	
APOE4_INDEX	0.24773641	0.23791899	1.0412637	0.302535018	
Metabolic_Index	0.39903878	0.13838307	2.8835809	0.005692417	**

-----

118. Results for hsa-miR-766-3p

Covariate	Beta	SE	t	p	Signif
(Intercept)	3.17774499	1.9092272	1.6644143	0.10234224	
GroupCI	0.95707126	0.3617761	2.6454794	0.01090094	*
SexMale	-0.13908986	0.3545381	-0.3923128	0.69650969	
Visit_GroupV2	0.14178078	0.2352531	0.6026735	0.54947458	
AgeAtVisit	0.01738511	0.0263741	0.6591736	0.51283641	
APOE4_INDEX	0.40379858	0.2466696	1.6370016	0.10796700	
Metabolic_Index	0.32499225	0.1426721	2.2778960	0.02708206	*

-----

119. Results for hsa-miR-324-3p

Covariate	Beta	SE	t	p	Signif
(Intercept)	2.40969202	1.71098243	1.4083675	0.165372550	
GroupCI	0.88077242	0.32803607	2.6849865	0.009886214	**
SexMale	-0.18639314	0.32002670	-0.5824300	0.562963220	
Visit_GroupV2	-0.06817075	0.25637808	-0.2658993	0.791438871	
AgeAtVisit	0.03344990	0.02356388	1.4195412	0.162108829	
APOE4_INDEX	0.41841832	0.22296353	1.8766223	0.066566316	.
Metabolic_Index	0.28481350	0.12909807	2.2061793	0.032118383	*

-----

120. Results for hsa-miR-146b-5p

Covariate	Beta	SE	t	p	Signif
(Intercept)	2.93068506	2.08449253	1.4059465	0.165604328	
GroupCI	1.13789252	0.39399966	2.8880546	0.005610409	**
SexMale	-0.31802642	0.38692648	-0.8219299	0.414817210	
Visit_GroupV2	0.08431916	0.21468036	0.3927661	0.696075283	
AgeAtVisit	0.02591054	0.02888415	0.8970507	0.373768534	
APOE4_INDEX	0.10741075	0.26800102	0.4007849	0.690197580	
Metabolic_Index	0.30590952	0.15524654	1.9704756	0.054043337	.

-----

121. Results for hsa-miR-584-5p

Covariate	Beta	SE	t	p	Signif
(Intercept)	2.83026734	1.82415421	1.5515505	0.127038468	
GroupCI	1.04894499	0.34484131	3.0418194	0.003730032	**
SexMale	-0.59315379	0.33832766	-1.7531933	0.085660334	.
Visit_GroupV2	-0.14655280	0.24579217	-0.5962468	0.553683824	
AgeAtVisit	0.02883591	0.02511944	1.1479517	0.256417969	
APOE4_INDEX	0.13398853	0.23482596	0.5705865	0.570819814	
Metabolic_Index	0.47352655	0.13712666	3.4532055	0.001132486	**

-----

122. Results for hsa-miR-889-3p

Covariate	Beta	SE	t	p	Signif
(Intercept)	3.28383106	2.06655017	1.5890401	0.119037312	
GroupCI	1.29363873	0.39273150	3.2939520	0.001927384	**
SexMale	-0.34139834	0.38143764	-0.8950305	0.375522393	
Visit_GroupV2	0.09549195	0.29403363	0.3247654	0.746860170	
AgeAtVisit	0.04083615	0.02846281	1.4347194	0.158264898	
APOE4_INDEX	0.12434238	0.26375238	0.4714360	0.639601873	
Metabolic_Index	-0.04570275	0.15353759	-0.2976649	0.767325704	

-----

123. Results for hsa-miR-101-3p

Covariate	Beta	SE	t	p	Signif
(Intercept)	3.14499543	2.13460105	1.4733411	0.147054398	

Covariate	Beta	SE	t	p	Signif
GroupCI	1.13372676	0.40438586	2.8035767	0.007222517	**
SexMale	-0.10254104	0.39317953	-0.2607995	0.795340061	
Visit_GroupV2	-0.13490194	0.26538480	-0.5083258	0.613505861	
AgeAtVisit	0.03441359	0.02940677	1.1702608	0.247556407	
APOE4_INDEX	0.27040108	0.27158273	0.9956490	0.324312213	
Metabolic_Index	0.12373646	0.15888135	0.7787979	0.439843714	

-----

124. Results for hsa-miR-487b-3p

Covariate	Beta	SE	t	p	Signif
(Intercept)	3.42070917	1.96337964	1.7422556	0.087572307	.
GroupCI	1.07614799	0.37239140	2.8898304	0.005673859	**
SexMale	-0.27846515	0.36531134	-0.7622680	0.449457931	
Visit_GroupV2	0.33974789	0.23761035	1.4298531	0.158938133	
AgeAtVisit	0.02164691	0.02714054	0.7975858	0.428858093	
APOE4_INDEX	0.27121518	0.25308227	1.0716483	0.288990287	
Metabolic_Index	0.13461103	0.14585678	0.9228987	0.360463576	

-----

125. Results for hsa-miR-30b-3p

Covariate	Beta	SE	t	p	Signif
(Intercept)	2.22376484	1.94575895	1.1428779	0.258648104	
GroupCI	1.00045888	0.37185517	2.6904531	0.009733223	**
SexMale	-0.26451025	0.36337430	-0.7279278	0.470125432	
Visit_GroupV2	0.13574226	0.26341421	0.5153187	0.608649771	
AgeAtVisit	0.03452261	0.02683369	1.2865396	0.204306016	
APOE4_INDEX	0.39873687	0.25273751	1.5776719	0.121082025	
Metabolic_Index	0.29073568	0.14654554	1.9839271	0.052886027	.

-----

126. Results for hsa-miR-499a-5p

Covariate	Beta	SE	t	p	Signif
(Intercept)	3.24345070	1.77938078	1.8227974	0.07473527	.
GroupCI	0.90338578	0.33876081	2.6667364	0.01048794	*
SexMale	-0.41453290	0.33187544	-1.2490617	0.21786313	
Visit_GroupV2	0.13030610	0.25823872	0.5045955	0.61621412	

Covariate	Beta	SE	t	p	Signif
AgeAtVisit	0.02053503	0.02441435	0.8411047	0.40457271	
APOE4_INDEX	0.61872782	0.23374109	2.6470649	0.01103165	*
Metabolic_Index	0.31909750	0.13356640	2.3890551	0.02098190	*

-----

127. Results for hsa-miR-136-3p

Covariate	Beta	SE	t	p	Signif
(Intercept)	2.14361628	2.04008139	1.0507504	0.298295478	
GroupCI	1.11638261	0.38684369	2.8858752	0.005696338	**
SexMale	-0.27494959	0.37888303	-0.7256846	0.471328205	
Visit_GroupV2	-0.06998713	0.21039839	-0.3326410	0.740760118	
AgeAtVisit	0.03801857	0.02827015	1.3448308	0.184592681	
APOE4_INDEX	0.12707518	0.26268553	0.4837540	0.630618030	
Metabolic_Index	0.34648811	0.15247387	2.2724425	0.027278285	*

-----

128. Results for hsa-miR-3605-5p

Covariate	Beta	SE	t	p	Signif
(Intercept)	4.461185383	2.01990183	2.2086150	0.03160426	*
GroupCI	1.011307388	0.38101212	2.6542656	0.01050367	*
SexMale	-0.412478388	0.37422283	-1.1022267	0.27540644	
Visit_GroupV2	0.032151221	0.22535862	0.1426669	0.88710111	
AgeAtVisit	0.004633783	0.02783748	0.1664584	0.86843768	
APOE4_INDEX	0.248628905	0.26114527	0.9520712	0.34543756	
Metabolic_Index	0.362520168	0.15007407	2.4156082	0.01923480	*

-----

129. Results for hsa-miR-134-5p

Covariate	Beta	SE	t	p	Signif
(Intercept)	3.586221262	2.00834550	1.7856595	0.080144918	.
GroupCI	1.065425946	0.38533887	2.7649065	0.007923858	**
SexMale	0.165012314	0.37096589	0.4448180	0.658347295	
Visit_GroupV2	0.336659218	0.27399500	1.2287057	0.224863321	
AgeAtVisit	0.024163175	0.02762592	0.8746558	0.385891867	
APOE4_INDEX	0.279754850	0.25997784	1.0760719	0.286996581	
Metabolic_Index	-0.002455619	0.15045119	-0.0163217	0.987041901	

-----

130. Results for hsa-miR-4488

Covariate	Beta	SE	t	p	Signif
(Intercept)	0.9376807	2.08758578	0.4491699	0.655217905	
GroupCI	1.1346006	0.39762681	2.8534308	0.006241075	**
SexMale	-0.4906769	0.38989196	-1.2584945	0.213961233	
Visit_GroupV2	-0.2658550	0.26641498	-0.9978981	0.323054436	
AgeAtVisit	0.0528068	0.02891406	1.8263363	0.073673718	.
APOE4_INDEX	0.3102738	0.27065209	1.1463937	0.256998950	
Metabolic_Index	0.3984183	0.15659958	2.5441853	0.014034712	*

-----

131. Results for hsa-miR-4429

Covariate	Beta	SE	t	p	Signif
(Intercept)	2.25823167	1.95936192	1.1525342	0.254467023	
GroupCI	1.04107548	0.37099296	2.8061866	0.007077622	**
SexMale	-0.52888764	0.36399788	-1.4529965	0.152340884	
Visit_GroupV2	-0.17282484	0.24865142	-0.6950487	0.490174679	
AgeAtVisit	0.03622499	0.02706405	1.3384909	0.186663938	
APOE4_INDEX	0.25482649	0.25195571	1.0113940	0.316595014	
Metabolic_Index	0.41896867	0.14649004	2.8600489	0.006120749	**

-----

132. Results for hsa-miR-181c-3p

Covariate	Beta	SE	t	p	Signif
(Intercept)	2.89355817	1.89082364	1.5303163	0.132340371	
GroupCI	0.98603596	0.35861206	2.7495895	0.008322304	**
SexMale	-0.54656477	0.35328400	-1.5470975	0.128245664	
Visit_GroupV2	-0.06310327	0.24130477	-0.2615086	0.794791479	
AgeAtVisit	0.02655575	0.02607096	1.0185948	0.313375933	
APOE4_INDEX	0.36832183	0.24529743	1.5015316	0.139606372	
Metabolic_Index	0.43855156	0.14211642	3.0858612	0.003327432	**

-----

133. Results for hsa-miR-92b-3p

Covariate	Beta	SE	t	p	Signif
(Intercept)	4.11802879	2.2060876	1.86666602	0.067673895	.
GroupCI	0.88305409	0.4236895	2.08420100	0.042144548	*
SexMale	0.02990336	0.4115249	0.07266476	0.942355733	
Visit_GroupV2	-0.34731571	0.3245576	-1.07012036	0.289579059	
AgeAtVisit	0.00962485	0.0302301	0.31838636	0.751485701	
APOE4_INDEX	0.80831277	0.2902888	2.78451231	0.007492555	**
Metabolic_Index	0.35687172	0.1670756	2.13599006	0.037478100	*

-----

134. Results for hsa-miR-369-5p

Covariate	Beta	SE	t	p	Signif
(Intercept)	2.74060808	1.88590810	1.4532034	0.152436550	
GroupCI	1.05759992	0.35845668	2.9504260	0.004824757	**
SexMale	-0.44156546	0.35089624	-1.2583933	0.214114122	
Visit_GroupV2	0.18321998	0.24692350	0.7420111	0.461564028	
AgeAtVisit	0.03100459	0.02596335	1.1941675	0.238066401	
APOE4_INDEX	0.17144826	0.24314354	0.7051319	0.484011456	
Metabolic_Index	0.31165369	0.14138992	2.2042144	0.032156773	*

-----

135. Results for hsa-miR-95-3p

Covariate	Beta	SE	t	p	Signif
(Intercept)	4.12754705	2.48309801	1.6622570	0.102695453	
GroupCI	1.21927584	0.47319370	2.5766950	0.012962011	*
SexMale	-0.17760810	0.46200479	-0.3844291	0.702284546	
Visit_GroupV2	-0.88773472	0.27410195	-3.2387027	0.002131895	**
AgeAtVisit	0.01996492	0.03430232	0.5820281	0.563153238	
APOE4_INDEX	0.36186479	0.32316994	1.1197353	0.268156772	
Metabolic_Index	0.36759353	0.18622718	1.9738984	0.053910453	.

-----

136. Results for hsa-miR-200c-3p

Covariate	Beta	SE	t	p	Signif
(Intercept)	2.24019534	2.37009544	0.9451920	0.34905037	

Covariate	Beta	SE	t	p	Signif
GroupCI	1.20004998	0.45343506	2.6465752	0.01080837	*
SexMale	-0.13326052	0.44100768	-0.3021728	0.76375832	
Visit_GroupV2	-0.44771967	0.27459405	-1.6304784	0.10920719	
AgeAtVisit	0.03869693	0.03276419	1.1810738	0.24309240	
APOE4_INDEX	0.23948438	0.30859708	0.7760423	0.44133455	
Metabolic_Index	0.38387891	0.17846802	2.1509674	0.03627535	*

-----

137. Results for hsa-miR-505-3p

Covariate	Beta	SE	t	p	Signif
(Intercept)	3.02573680	1.83533237	1.6486043	0.105752645	
GroupCI	0.90187162	0.35220940	2.5606120	0.013645839	*
SexMale	-0.04366311	0.34108540	-0.1280123	0.898673537	
Visit_GroupV2	-0.28106878	0.24275955	-1.1578073	0.252665320	
AgeAtVisit	0.02864895	0.02522574	1.1357029	0.261713880	
APOE4_INDEX	0.21377885	0.23876089	0.8953679	0.375053875	
Metabolic_Index	0.39103315	0.13949910	2.8031232	0.007277677	**

-----

138. Results for hsa-miR-150-3p

Covariate	Beta	SE	t	p	Signif
(Intercept)	3.95629544	2.07844836	1.9034851	0.06234247	.
GroupCI	0.99220825	0.38728599	2.5619523	0.01324857	*
SexMale	-0.44332511	0.38160854	-1.1617274	0.25048364	
Visit_GroupV2	-0.18283737	0.22225638	-0.8226417	0.41434972	
AgeAtVisit	0.01010431	0.02872772	0.3517267	0.72641963	
APOE4_INDEX	0.26076982	0.26436418	0.9864037	0.32835860	
Metabolic_Index	0.39664160	0.15279058	2.5959820	0.01213956	*

-----

139. Results for hsa-miR-184

Covariate	Beta	SE	t	p	Signif
(Intercept)	2.56679906	3.34936959	0.7663529	0.446845843	
GroupCI	1.48446638	0.62806399	2.3635591	0.021776317	*
SexMale	-0.07260012	0.61983438	-0.1171283	0.907198478	
Visit_GroupV2	-0.56551448	0.43895084	-1.2883321	0.203192713	

Covariate	Beta	SE	t	p	Signif
AgeAtVisit	0.06069426	0.04641841	1.3075473	0.196636666	
APOE4_INDEX	0.86580861	0.42502980	2.0370539	0.046621787	*
Metabolic_Index	0.67891220	0.24529588	2.7677277	0.007744456	**

-----

140. Results for hsa-miR-141-3p

Covariate	Beta	SE	t	p	Signif
(Intercept)	1.74120908	2.34324065	0.7430774	0.460815066	
GroupCI	1.33802304	0.44454875	3.0098455	0.004042395	**
SexMale	-0.28571919	0.43251672	-0.6605969	0.511818422	
Visit_GroupV2	-0.12551176	0.27908047	-0.4497332	0.654792231	
AgeAtVisit	0.04744395	0.03251838	1.4589889	0.150647738	
APOE4_INDEX	-0.19264496	0.29894113	-0.6444244	0.522163619	
Metabolic_Index	0.39575436	0.17550521	2.2549437	0.028420778	*

-----

141. Results for hsa-miR-505-5p

Covariate	Beta	SE	t	p	Signif
(Intercept)	2.70704503	1.66261368	1.6281864	0.10662462	
GroupCI	0.83209178	0.32029844	2.5978639	0.01079301	*
SexMale	-0.16368532	0.30525670	-0.5362219	0.59299324	
Visit_GroupV2	0.59971035	0.29736165	2.0167710	0.04639384	*
AgeAtVisit	0.04023034	0.02262712	1.7779700	0.07844117	.
APOE4_INDEX	0.52373824	0.21262564	2.4631942	0.01547455	*
Metabolic_Index	0.06896093	0.12621640	0.5463706	0.58602531	

-----

142. Results for hsa-miR-1304-3p

Covariate	Beta	SE	t	p	Signif
(Intercept)	4.968996279	1.86240288	2.6680566	0.010054945	*
GroupCI	0.959462334	0.35773695	2.6820331	0.009692514	**
SexMale	0.036276211	0.34242024	0.1059406	0.916022688	
Visit_GroupV2	0.565050279	0.30126931	1.8755653	0.066134498	.
AgeAtVisit	0.006032981	0.02536595	0.2378378	0.812908544	
APOE4_INDEX	0.270621718	0.23963519	1.1293071	0.263768326	
Metabolic_Index	-0.085368613	0.13972463	-0.6109776	0.543782007	

-----

143. Results for hsa-miR-125a-5p

Covariate	Beta	SE	t	p	Signif
(Intercept)	8.18136014	1.87578471	4.3615667	3.137182e-05	***
GroupCI	0.76123473	0.35099869	2.1687680	3.246543e-02	*
SexMale	-0.22795737	0.33114614	-0.6883890	4.927980e-01	
Visit_GroupV2	0.11628039	0.32481319	0.3579916	7.211024e-01	
AgeAtVisit	-0.02506009	0.02529846	-0.9905779	3.242769e-01	
APOE4_INDEX	0.15640123	0.23089229	0.6773774	4.997270e-01	
Metabolic_Index	0.12649762	0.13868954	0.9120920	3.639087e-01	

-----

144. Results for hsa-miR-26b-3p

Covariate	Beta	SE	t	p	Signif
(Intercept)	4.465448594	1.98653282	2.2478605	0.02888940	*
GroupCI	0.959760785	0.37449117	2.5628395	0.01334222	*
SexMale	-0.533350873	0.36783449	-1.4499752	0.15312189	
Visit_GroupV2	0.174029609	0.24420817	0.7126281	0.47928881	
AgeAtVisit	0.004971334	0.02736452	0.1816708	0.85655348	
APOE4_INDEX	0.300455511	0.25535886	1.1766011	0.24475868	
Metabolic_Index	0.323288463	0.14779112	2.1874688	0.03326694	*

-----

145. Results for hsa-miR-1299

Covariate	Beta	SE	t	p	Signif
(Intercept)	6.01558047	2.01493269	2.9854995	0.004308909	**
GroupCI	0.91391505	0.37791744	2.4182928	0.019132462	*
SexMale	-0.30683614	0.36967995	-0.8300048	0.410331698	
Visit_GroupV2	0.26345113	0.26794241	0.9832379	0.330044679	
AgeAtVisit	-0.01628566	0.02763506	-0.5893115	0.558204918	
APOE4_INDEX	0.28731708	0.25651314	1.1200872	0.267825098	
Metabolic_Index	0.28199950	0.14887309	1.8942275	0.063764573	.

-----

146. Results for hsa-miR-337-5p

Covariate	Beta	SE	t	p	Signif
(Intercept)	2.22428850	2.13918626	1.0397825	0.303308921	
GroupCI	1.08767339	0.40585072	2.6799839	0.009869296	**
SexMale	-0.36423163	0.39920947	-0.9123822	0.365828447	
Visit_GroupV2	0.25340363	0.21904122	1.1568765	0.252672793	
AgeAtVisit	0.03570318	0.02970385	1.2019714	0.234884846	
APOE4_INDEX	0.28453005	0.27585887	1.0314334	0.307168081	
Metabolic_Index	0.30839346	0.15914397	1.9378269	0.058148809	.

-----

147. Results for hsa-miR-10399-5p

Covariate	Beta	SE	t	p	Signif
(Intercept)	3.13924436	2.03761974	1.5406429	0.12952676	
GroupCI	0.95706343	0.38339109	2.4963111	0.01578980	*
SexMale	-0.24421427	0.37671195	-0.6482785	0.51968517	
Visit_GroupV2	-0.11462363	0.22486115	-0.5097529	0.61240239	
AgeAtVisit	0.02041549	0.02818564	0.7243222	0.47214605	
APOE4_INDEX	0.24343514	0.26158823	0.9306043	0.35640058	
Metabolic_Index	0.39967178	0.15142098	2.6394743	0.01096052	*

-----

148. Results for hsa-miR-4758-5p

Covariate	Beta	SE	t	p	Signif
(Intercept)	2.94445261	3.47936098	0.8462625	0.401158918	
GroupCI	1.71242888	0.65250035	2.6244107	0.011276657	*
SexMale	-0.69397004	0.64160826	-1.0816102	0.284255451	
Visit_GroupV2	0.21137626	0.38044017	0.5556097	0.580783419	
AgeAtVisit	0.05080082	0.04827049	1.0524199	0.297316104	
APOE4_INDEX	0.56841129	0.44517929	1.2768143	0.207157365	
Metabolic_Index	0.77005229	0.25648267	3.0023560	0.004059188	**

-----

149. Results for hsa-miR-10401-3p

Covariate	Beta	SE	t	p	Signif
(Intercept)	3.32243478	2.01834656	1.6461171	0.10576808	

Covariate	Beta	SE	t	p	Signif
GroupCI	0.92211123	0.38028016	2.4248208	0.01882392	*
SexMale	-0.16608136	0.37376479	-0.4443473	0.65863442	
Visit_GroupV2	-0.09050488	0.21650583	-0.4180251	0.67764910	
AgeAtVisit	0.01640812	0.02792094	0.5876635	0.55930027	
APOE4_INDEX	0.33328134	0.25937459	1.2849421	0.20450374	
Metabolic_Index	0.36601268	0.14993237	2.4411852	0.01807750	*

-----

150. Results for hsa-miR-100-5p

Covariate	Beta	SE	t	p	Signif
(Intercept)	9.70659296	3.59125263	2.7028433	0.009212615	**
GroupCI	1.53175634	0.67521949	2.2685310	0.027388067	*
SexMale	-0.54832009	0.66300568	-0.8270217	0.411926251	
Visit_GroupV2	-0.39193041	0.39537277	-0.9912934	0.326038642	
AgeAtVisit	-0.02997683	0.04994105	-0.6002444	0.550896901	
APOE4_INDEX	0.87388616	0.45848373	1.9060353	0.062065247	.
Metabolic_Index	0.67146055	0.26514722	2.5324065	0.014320609	*

-----

151. Results for hsa-miR-32-3p

Covariate	Beta	SE	t	p	Signif
(Intercept)	1.95925155	2.20626125	0.8880415	0.378671732	
GroupCI	1.19932302	0.41814899	2.8681715	0.005982718	**
SexMale	-0.63111493	0.40961595	-1.5407480	0.129536953	
Visit_GroupV2	0.15764747	0.22830661	0.6905077	0.492993776	
AgeAtVisit	0.04613149	0.03061439	1.5068565	0.137993537	
APOE4_INDEX	0.18167169	0.28483497	0.6378139	0.526437427	
Metabolic_Index	0.26988865	0.16436513	1.6420067	0.106718037	

-----

152. Results for hsa-miR-29c-5p

Covariate	Beta	SE	t	p	Signif
(Intercept)	1.98286080	1.70850103	1.1605851	0.25164901	
GroupCI	0.78270483	0.32491223	2.4089731	0.01995334	*
SexMale	-0.10847307	0.31805880	-0.3410472	0.73458203	
Visit_GroupV2	-0.12663766	0.23867812	-0.5305792	0.59819882	

Covariate	Beta	SE	t	p	Signif
AgeAtVisit	0.03467533	0.02357471	1.4708700	0.14796409	
APOE4_INDEX	0.51126046	0.22161560	2.3069696	0.02549264	*
Metabolic_Index	0.43873370	0.12882358	3.4056941	0.00135690	**

-----

153. Results for hsa-miR-187-3p

Covariate	Beta	SE	t	p	Signif
(Intercept)	3.57773961	1.86776772	1.9155164	0.06100706	.
GroupCI	0.81953965	0.35075386	2.3365093	0.02340655	*
SexMale	-0.18047895	0.34486874	-0.5233265	0.60299979	
Visit_GroupV2	-0.03407516	0.23347659	-0.1459468	0.88453521	
AgeAtVisit	0.01384594	0.02569113	0.5389386	0.59225987	
APOE4_INDEX	0.30426319	0.23901472	1.2729893	0.20875905	
Metabolic_Index	0.41864102	0.13919099	3.0076732	0.00407094	**

-----

154. Results for hsa-miR-4446-3p

Covariate	Beta	SE	t	p	Signif
(Intercept)	2.84477263	1.69548400	1.677853	0.099881106	.
GroupCI	0.82575779	0.32329390	2.554202	0.013874419	*
SexMale	-0.33620118	0.31676950	-1.061343	0.293848855	
Visit_GroupV2	0.25873428	0.25855918	1.000677	0.322004573	
AgeAtVisit	0.02542564	0.02329548	1.091441	0.280531216	
APOE4_INDEX	0.41775382	0.22025613	1.896673	0.063901905	.
Metabolic_Index	0.39217751	0.12861303	3.049283	0.003727764	**

-----

155. Results for hsa-miR-18a-5p

Covariate	Beta	SE	t	p	Signif
(Intercept)	3.50923559	1.73856976	2.0184612	0.049639806	*
GroupCI	0.80355261	0.33078070	2.4292608	0.019264095	*
SexMale	-0.45342487	0.32242302	-1.4063043	0.166629530	
Visit_GroupV2	0.24639298	0.26601802	0.9262266	0.359360951	
AgeAtVisit	0.01937623	0.02376933	0.8151777	0.419344024	
APOE4_INDEX	0.46372585	0.22699484	2.0428916	0.047057618	*
Metabolic_Index	0.39503905	0.13198971	2.9929535	0.004510647	**

-----

156. Results for hsa-miR-181a-3p

Covariate	Beta	SE	t	p	Signif
(Intercept)	4.123318158	1.94679078	2.1180078	0.03920922	*
GroupCI	0.855003452	0.36876378	2.3185668	0.02458135	*
SexMale	-0.251929875	0.36191925	-0.6960942	0.48961923	
Visit_GroupV2	0.056635400	0.25833622	0.2192314	0.82736958	
AgeAtVisit	0.008375525	0.02678113	0.3127398	0.75578965	
APOE4_INDEX	0.402442543	0.25160714	1.5994878	0.11606402	
Metabolic_Index	0.287163212	0.14544468	1.9743810	0.05392018	.

-----

157. Results for hsa-miR-6788-5p

Covariate	Beta	SE	t	p	Signif
(Intercept)	2.84305306	1.91091740	1.4877948	0.142804041	
GroupCI	0.86529518	0.35953071	2.4067351	0.019658087	*
SexMale	-0.25809196	0.35410578	-0.7288555	0.469338202	
Visit_GroupV2	0.04608423	0.21706838	0.2123028	0.832695232	
AgeAtVisit	0.02262400	0.02642892	0.8560321	0.395881268	
APOE4_INDEX	0.38881189	0.24563284	1.5828987	0.119467743	
Metabolic_Index	0.38463296	0.14194849	2.7096658	0.009083128	**

-----

158. Results for hsa-miR-323a-3p

Covariate	Beta	SE	t	p	Signif
(Intercept)	2.9230131	1.9248025	1.5186042	0.13493789	
GroupCI	0.9538021	0.3634638	2.6242012	0.01138332	*
SexMale	-0.2940294	0.3571783	-0.8232008	0.41416483	
Visit_GroupV2	0.4447377	0.2257340	1.9701849	0.05416966	.
AgeAtVisit	0.0281998	0.0265850	1.0607411	0.29372477	
APOE4_INDEX	0.2712121	0.2469858	1.0980881	0.27724028	
Metabolic_Index	0.1815606	0.1426619	1.2726628	0.20881625	

-----

159. Results for hsa-miR-548ax

Covariate	Beta	SE	t	p	Signif
(Intercept)	4.903595094	2.17503376	2.25449148	0.02837770	*
GroupCI	1.062837026	0.40943381	2.59587023	0.01221830	*
SexMale	-0.473443031	0.40273410	-1.17557224	0.24508786	
Visit_GroupV2	0.457009463	0.22460017	2.03476902	0.04695588	*
AgeAtVisit	0.001386629	0.03001468	0.04619836	0.96332804	
APOE4_INDEX	0.134250662	0.27879732	0.48153498	0.63214440	
Metabolic_Index	0.187811296	0.16071404	1.16860543	0.24785994	

-----

160. Results for hsa-miR-642a-3p

Covariate	Beta	SE	t	p	Signif
(Intercept)	2.9463848	2.24648908	1.3115509	0.195506839	
GroupCI	0.9804788	0.42514681	2.3062122	0.025170102	*
SexMale	-0.1375460	0.41617550	-0.3305000	0.742367176	
Visit_GroupV2	-0.2244327	0.25500992	-0.8800942	0.382909480	
AgeAtVisit	0.0226430	0.03105925	0.7290258	0.469298641	
APOE4_INDEX	0.2432989	0.28923725	0.8411740	0.404151383	
Metabolic_Index	0.4708495	0.16771915	2.8073690	0.007043906	**

-----

161. Results for hsa-miR-671-3p

Covariate	Beta	SE	t	p	Signif
(Intercept)	3.21058100	1.83895934	1.7458684	0.087047046	.
GroupCI	0.84820735	0.34596102	2.4517425	0.017795507	*
SexMale	-0.24815165	0.34050243	-0.7287808	0.469574763	
Visit_GroupV2	0.24147780	0.24911583	0.9693395	0.337095294	
AgeAtVisit	0.01720667	0.02527924	0.6806640	0.499260289	
APOE4_INDEX	0.30495913	0.23634143	1.2903329	0.202938753	
Metabolic_Index	0.44654807	0.13757972	3.2457404	0.002105353	**

-----

162. Results for hsa-miR-942-5p

Covariate	Beta	SE	t	p	Signif
(Intercept)	1.71430540	1.82580941	0.9389290	0.35233254	

Covariate	Beta	SE	t	p	Signif
GroupCI	0.84744531	0.34618960	2.4479225	0.01796385	*
SexMale	-0.14332208	0.33967141	-0.4219433	0.67489584	
Visit_GroupV2	0.18915724	0.23595195	0.8016770	0.42657685	
AgeAtVisit	0.03728172	0.02525167	1.4764063	0.14617722	
APOE4_INDEX	0.41223995	0.23639730	1.7438437	0.08740267	.
Metabolic_Index	0.38822567	0.13702390	2.8332698	0.00665178	**

-----

163. Results for hsa-miR-421

Covariate	Beta	SE	t	p	Signif
(Intercept)	5.66196285	1.98621303	2.8506322	0.005297789	**
GroupCI	0.97031525	0.36639835	2.6482523	0.009399876	**
SexMale	0.32694091	0.34986254	0.9344839	0.352300098	
Visit_GroupV2	0.94789887	0.34277445	2.7653720	0.006769397	**
AgeAtVisit	0.01127069	0.02697038	0.4178914	0.676920384	
APOE4_INDEX	0.03916645	0.24023011	0.1630372	0.870817391	
Metabolic_Index	-0.05214623	0.14504703	-0.3595126	0.719967945	

-----

164. Results for hsa-miR-6815-5p

Covariate	Beta	SE	t	p	Signif
(Intercept)	3.19912487	1.9922736	1.6057659	0.1145207811	
GroupCI	0.88381969	0.3742461	2.3616006	0.0220664371	*
SexMale	-0.13914621	0.3675553	-0.3785722	0.7065821763	
Visit_GroupV2	-0.04085854	0.2286139	-0.1787229	0.8588657094	
AgeAtVisit	0.01624490	0.0275291	0.5900992	0.5577384219	
APOE4_INDEX	0.12589681	0.2557661	0.4922342	0.6246731014	
Metabolic_Index	0.62834242	0.1491034	4.2141388	0.0001027409	***

-----

165. Results for hsa-miR-133a-3p

Covariate	Beta	SE	t	p	Signif
(Intercept)	0.69966732	3.98665302	0.17550244	0.86134937	
GroupCI	1.65105603	0.75385039	2.19016405	0.03290487	*
SexMale	0.06051376	0.74210017	0.08154393	0.93531459	
Visit_GroupV2	-0.12829953	0.32198991	-0.39845824	0.69188288	

Covariate	Beta	SE	t	p	Signif
AgeAtVisit	0.06898526	0.05546234	1.24382177	0.21900343	
APOE4_INDEX	1.05602973	0.51720358	2.04180671	0.04612908	*
Metabolic_Index	0.62891013	0.29626832	2.12277209	0.03843119	*

-----

166. Results for hsa-miR-431-5p

Covariate	Beta	SE	t	p	Signif
(Intercept)	2.6338531	1.65743130	1.5891175	0.11890717	
GroupCI	0.7757612	0.31758496	2.4426889	0.01849177	*
SexMale	-0.2410031	0.30948644	-0.7787193	0.44014604	
Visit_GroupV2	0.2903784	0.26310126	1.1036756	0.27549329	
AgeAtVisit	0.0312043	0.02274632	1.3718397	0.17678955	
APOE4_INDEX	0.5183066	0.21689797	2.3896332	0.02103432	*
Metabolic_Index	0.2475315	0.12539302	1.9740455	0.05441966	.

-----

167. Results for hsa-miR-1273h-5p

Covariate	Beta	SE	t	p	Signif
(Intercept)	4.33860421	1.89206918	2.2930473	0.02601607	*
GroupCI	0.85628970	0.35972212	2.3804200	0.02108061	*
SexMale	-0.29960512	0.35202949	-0.8510796	0.39871683	
Visit_GroupV2	0.43677785	0.25830563	1.6909343	0.09697311	.
AgeAtVisit	0.01111064	0.02596063	0.4279802	0.67047350	
APOE4_INDEX	0.33513160	0.24445855	1.3709138	0.17642489	
Metabolic_Index	0.08968934	0.14082650	0.6368783	0.52706216	

-----

168. Results for hsa-miR-145-5p

Covariate	Beta	SE	t	p	Signif
(Intercept)	3.68079270	2.33841164	1.5740568	0.12151702	
GroupCI	1.14495032	0.43967437	2.6040870	0.01196953	*
SexMale	-0.51916374	0.43212484	-1.2014207	0.23501135	
Visit_GroupV2	0.08683941	0.27125747	0.3201365	0.75014295	
AgeAtVisit	0.02795547	0.03230056	0.8654793	0.39073652	
APOE4_INDEX	0.08854541	0.29786111	0.2972708	0.76743971	
Metabolic_Index	0.23290263	0.17343709	1.3428652	0.18512641	

-----

169. Results for hsa-miR-25-5p

Covariate	Beta	SE	t	p	Signif
(Intercept)	3.922544054	1.96932673	1.9918198	0.05156884	.
GroupCI	0.734787851	0.36879108	1.9924230	0.05150065	.
SexMale	-0.418904215	0.36353276	-1.1523149	0.25437715	
Visit_GroupV2	0.111245196	0.25641166	0.4338539	0.66616040	
AgeAtVisit	0.008196088	0.02709483	0.3024964	0.76346097	
APOE4_INDEX	0.415856036	0.25269845	1.6456612	0.10576831	
Metabolic_Index	0.530506006	0.14753602	3.5957728	0.00071130	***

-----

170. Results for hsa-miR-1283

Covariate	Beta	SE	t	p	Signif
(Intercept)	1.03451127	1.86162336	0.5557038	0.580877033	
GroupCI	0.87219242	0.35040421	2.4891037	0.016158912	*
SexMale	-0.44432836	0.34550797	-1.2860148	0.204327082	
Visit_GroupV2	-0.06514310	0.22587242	-0.2884066	0.774220787	
AgeAtVisit	0.04971004	0.02577583	1.9285528	0.059440142	.
APOE4_INDEX	0.42397132	0.24013867	1.7655270	0.083545498	.
Metabolic_Index	0.45846491	0.13905557	3.2969905	0.001797565	**

-----

171. Results for hsa-miR-194-5p

Covariate	Beta	SE	t	p	Signif
(Intercept)	4.05167182	2.21675653	1.8277478	0.073342521	.
GroupCI	1.03457221	0.41631805	2.4850525	0.016217106	*
SexMale	-0.71476586	0.41030845	-1.7420208	0.087432869	.
Visit_GroupV2	0.05375773	0.24756250	0.2171481	0.828944907	
AgeAtVisit	0.01556389	0.03055584	0.5093588	0.612661253	
APOE4_INDEX	0.09858059	0.28340830	0.3478394	0.729368588	
Metabolic_Index	0.44521880	0.16525110	2.6941956	0.009484745	**

-----

172. Results for hsa-miR-363-3p

Covariate	Beta	SE	t	p	Signif
(Intercept)	2.4637336	1.98850471	1.2389881	0.22090463	
GroupCI	0.8165558	0.37158099	2.1975178	0.03245390	*
SexMale	-0.3276439	0.36605205	-0.8950746	0.37486528	
Visit_GroupV2	-0.3063024	0.24463989	-1.2520542	0.21614377	
AgeAtVisit	0.0313788	0.02737802	1.1461310	0.25697968	
APOE4_INDEX	0.4094690	0.25414626	1.6111548	0.11319051	
Metabolic_Index	0.4470131	0.14751511	3.0302869	0.00379882	**

-----

173. Results for hsa-miR-3120-3p

Covariate	Beta	SE	t	p	Signif
(Intercept)	5.885891161	2.00223272	2.9396639	0.005043689	**
GroupCI	0.845949108	0.38333965	2.2067874	0.032153593	*
SexMale	-0.180591086	0.36934374	-0.4889513	0.627106701	
Visit_GroupV2	0.447279798	0.30660456	1.4588165	0.151139711	
AgeAtVisit	-0.003285106	0.02734159	-0.1201505	0.904866271	
APOE4_INDEX	0.203100816	0.25835479	0.7861314	0.435660793	
Metabolic_Index	-0.019305598	0.14952423	-0.1291135	0.897808239	

-----

174. Results for hsa-miR-133b

Covariate	Beta	SE	t	p	Signif
(Intercept)	1.69292921	2.92972979	0.5778448	0.56602235	
GroupCI	1.17289384	0.55157839	2.1264318	0.03854344	*
SexMale	-0.29077007	0.54718330	-0.5313943	0.59755308	
Visit_GroupV2	-0.24784204	0.30706003	-0.8071452	0.42349433	
AgeAtVisit	0.04346612	0.04082218	1.0647672	0.29221460	
APOE4_INDEX	0.77380760	0.38354541	2.0175123	0.04915190	*
Metabolic_Index	0.55561282	0.21804060	2.5482080	0.01402265	*

-----

175. Results for hsa-miR-424-3p

Covariate	Beta	SE	t	p	Signif
(Intercept)	3.91139514	1.99135723	1.9641856	0.05529814	.

Covariate	Beta	SE	t	p	Signif
GroupCI	0.76048512	0.38003583	2.0010879	0.05103967	.
SexMale	0.12018746	0.36801016	0.3265873	0.74539615	
Visit_GroupV2	-0.42890484	0.28136208	-1.5243875	0.13395713	
AgeAtVisit	0.01542077	0.02732966	0.5642504	0.57520415	
APOE4_INDEX	0.33970209	0.25634241	1.3251888	0.19136595	
Metabolic_Index	0.31781753	0.14995592	2.1194063	0.03924120	*

-----

176. Results for hsa-miR-196a-5p

Covariate	Beta	SE	t	p	Signif
(Intercept)	2.44919191	2.53815123	0.96495113	0.33965426	
GroupCI	1.04987267	0.49039831	2.14085706	0.03766052	*
SexMale	-0.19057720	0.47617155	-0.40022803	0.69085566	
Visit_GroupV2	-0.01624816	0.37003097	-0.04391027	0.96516747	
AgeAtVisit	0.04578627	0.03515674	1.30234676	0.19933138	
APOE4_INDEX	0.47413949	0.33264102	1.42537891	0.16085072	
Metabolic_Index	0.26757116	0.19188492	1.39443557	0.16993668	

-----

177. Results for hsa-miR-532-3p

Covariate	Beta	SE	t	p	Signif
(Intercept)	4.616237727	1.78779011	2.58209155	0.01310255	*
GroupCI	0.592708844	0.33857006	1.75062390	0.08674133	.
SexMale	-0.010280925	0.32742666	-0.03139917	0.97508834	
Visit_GroupV2	0.103515992	0.27970839	0.37008540	0.71303353	
AgeAtVisit	0.009490733	0.02429907	0.39058015	0.69792773	
APOE4_INDEX	0.463791609	0.22887685	2.02638064	0.04860266	*
Metabolic_Index	0.208691015	0.13431093	1.55379025	0.12715329	

-----

178. Results for hsa-miR-424-5p

Covariate	Beta	SE	t	p	Signif
(Intercept)	6.252593975	1.97555278	3.1649845	0.002054161	**
GroupCI	1.055217618	0.36561767	2.8861231	0.004776674	**
SexMale	0.332626838	0.34769646	0.9566587	0.341041212	
Visit_GroupV2	0.375378584	0.34209512	1.0972930	0.275142384	

Covariate	Beta	SE	t	p	Signif
AgeAtVisit	0.004427621	0.02680977	0.1651495	0.869158920	
APOE4_INDEX	0.033992532	0.23885240	0.1423161	0.887116034	
Metabolic_Index	-0.256070864	0.14338869	-1.7858512	0.077147310	.

-----

179. Results for hsa-miR-17-5p

Covariate	Beta	SE	t	p	Signif
(Intercept)	4.08448181	1.8577639	2.1986012	0.03324562	*
GroupCI	0.80169576	0.3580812	2.2388655	0.03030552	*
SexMale	-0.16625381	0.3461297	-0.4803224	0.63339340	
Visit_GroupV2	0.09337014	0.2758422	0.3384911	0.73661376	
AgeAtVisit	0.02665767	0.0254224	1.0485900	0.30013038	
APOE4_INDEX	0.31437172	0.2419857	1.2991336	0.20070918	
Metabolic_Index	0.07714168	0.1412150	0.5462713	0.58765713	

-----

180. Results for hsa-miR-484

Covariate	Beta	SE	t	p	Signif
(Intercept)	2.73854192	2.25106625	1.2165532	0.22977552	
GroupCI	1.03006772	0.43067635	2.3917444	0.02077119	*
SexMale	-0.21884222	0.41850890	-0.5229093	0.60346432	
Visit_GroupV2	-0.04577995	0.31819074	-0.1438758	0.88620627	
AgeAtVisit	0.04755226	0.03104187	1.5318751	0.13217662	
APOE4_INDEX	0.09804189	0.29075411	0.3371986	0.73745035	
Metabolic_Index	0.18975956	0.16936302	1.1204309	0.26815456	

-----

181. Results for hsa-miR-191-3p

Covariate	Beta	SE	t	p	Signif
(Intercept)	5.981416197	1.95144681	3.0651188	0.003478208	**
GroupCI	0.795191309	0.36717263	2.1657151	0.035046800	*
SexMale	-0.258526069	0.35921063	-0.7197061	0.475000848	
Visit_GroupV2	0.684831306	0.24942793	2.7456080	0.008328804	**
AgeAtVisit	-0.007914183	0.02674538	-0.2959084	0.768505070	
APOE4_INDEX	0.340850167	0.24927410	1.3673710	0.177522691	
Metabolic_Index	-0.040143336	0.14385817	-0.2790480	0.781339714	

-----

182. Results for hsa-miR-28-5p

Covariate	Beta	SE	t	p	Signif
(Intercept)	4.723626647	1.97685124	2.3894700	0.02041115	*
GroupCI	0.813441382	0.37099561	2.1925903	0.03268624	*
SexMale	-0.396887659	0.36524158	-1.0866443	0.28204353	
Visit_GroupV2	0.042739081	0.20140359	0.2122062	0.83274893	
AgeAtVisit	0.004756204	0.02724995	0.1745400	0.86209695	
APOE4_INDEX	0.337210263	0.25264918	1.3346976	0.18760205	
Metabolic_Index	0.193324294	0.14579820	1.3259718	0.19045687	

-----

183. Results for hsa-miR-501-3p

Covariate	Beta	SE	t	p	Signif
(Intercept)	3.56105893	2.02464585	1.7588552	0.0844351143	.
GroupCI	0.76104774	0.37911392	2.0074381	0.0498680657	*
SexMale	-0.26527548	0.37339985	-0.7104328	0.4805829814	
Visit_GroupV2	-0.08887233	0.22608702	-0.3930890	0.6958469806	
AgeAtVisit	0.01315026	0.02795021	0.4704889	0.6399565418	
APOE4_INDEX	0.33525277	0.25901007	1.2943619	0.2012105242	
Metabolic_Index	0.52646299	0.15074820	3.4923334	0.0009807375	***

-----

184. Results for hsa-miR-4508

Covariate	Beta	SE	t	p	Signif
(Intercept)	3.20589834	1.92266161	1.6674272	0.098550971	.
GroupCI	0.71310506	0.36620961	1.9472593	0.054301841	.
SexMale	-0.42166724	0.35457533	-1.1892176	0.237163554	
Visit_GroupV2	-0.05513759	0.34425721	-0.1601639	0.873074251	
AgeAtVisit	0.02810969	0.02611176	1.0765146	0.284281346	
APOE4_INDEX	0.22112863	0.24704755	0.8950853	0.372885883	
Metabolic_Index	0.51240535	0.14748201	3.4743584	0.000757864	***

-----

185. Results for hsa-miR-7-5p

Covariate	Beta	SE	t	p	Signif
(Intercept)	9.44920698	2.14418572	4.40689763	0.000055625	***
GroupCI	0.88956156	0.39979421	2.22504860	0.030624940	*
SexMale	-0.02498829	0.39898686	-0.06262935	0.950311680	
Visit_GroupV2	-0.54356081	0.26170243	-2.07701854	0.042964326	*
AgeAtVisit	-0.00586152	0.02988603	-0.19612910	0.845305113	
APOE4_INDEX	0.02716663	0.27718583	0.09800872	0.922317727	
Metabolic_Index	0.50560807	0.15562888	3.24880614	0.002075808	**

-----

186. Results for hsa-miR-497-5p

Covariate	Beta	SE	t	p	Signif
(Intercept)	3.986923491	1.94547262	2.04933416	0.04573321	*
GroupCI	0.920441479	0.37151803	2.47751499	0.01667965	*
SexMale	-0.497414046	0.35843962	-1.38772062	0.17142068	
Visit_GroupV2	0.330170598	0.30424577	1.08521014	0.28307130	
AgeAtVisit	0.032043944	0.02666627	1.20166555	0.23519531	
APOE4_INDEX	0.004360832	0.24796538	0.01758645	0.98603927	
Metabolic_Index	0.085540290	0.14584660	0.58650862	0.56019181	

-----

187. Results for hsa-miR-4286

Covariate	Beta	SE	t	p	Signif
(Intercept)	4.472563176	2.16580995	2.0650765	0.04376714	*
GroupCI	0.907661180	0.40876933	2.2204728	0.03063574	*
SexMale	-0.445261918	0.40157723	-1.1087828	0.27247452	
Visit_GroupV2	0.308247435	0.23758530	1.2974180	0.20004415	
AgeAtVisit	0.009069606	0.02986424	0.3036945	0.76253646	
APOE4_INDEX	0.155877089	0.27792035	0.5608697	0.57722377	
Metabolic_Index	0.212225198	0.16078038	1.3199695	0.19245521	

-----

188. Results for hsa-miR-20a-5p

Covariate	Beta	SE	t	p	Signif
(Intercept)	3.35291391	2.11516707	1.5851769	0.1192787	

Covariate	Beta	SE	t	p	Signif
GroupCI	0.82466255	0.40064222	2.0583516	0.0448266	*
SexMale	-0.20063416	0.39448025	-0.5086038	0.6132837	
Visit_GroupV2	0.12490224	0.29463164	0.4239268	0.6734512	
AgeAtVisit	0.04098243	0.02918241	1.4043536	0.1664417	
APOE4_INDEX	0.68259500	0.27261299	2.5038975	0.0156182	*
Metabolic_Index	0.08644318	0.15769247	0.5481757	0.5860289	

-----

189. Results for hsa-miR-186-5p

Covariate	Beta	SE	t	p	Signif
(Intercept)	1.813030020	2.65143717	0.68379143	0.49712571	
GroupCI	1.197872104	0.50005722	2.39547006	0.02021322	*
SexMale	-0.112581563	0.49210386	-0.22877602	0.81993531	
Visit_GroupV2	0.019497055	0.22925713	0.08504449	0.93255077	
AgeAtVisit	0.050358439	0.03678366	1.36904386	0.17683721	
APOE4_INDEX	0.004454869	0.34071597	0.01307502	0.98961769	
Metabolic_Index	0.231474841	0.19735709	1.17287321	0.24616148	

-----

190. Results for hsa-miR-181a-2-3p

Covariate	Beta	SE	t	p	Signif
(Intercept)	4.13058221	2.24534593	1.83961952	0.07184221	.
GroupCI	0.89673682	0.42298008	2.12004507	0.03905338	*
SexMale	-0.65133745	0.41708650	-1.56163635	0.12476332	
Visit_GroupV2	0.01360943	0.27045668	0.05032017	0.96007034	
AgeAtVisit	0.01706777	0.03094533	0.55154574	0.58374816	
APOE4_INDEX	0.34964934	0.29031928	1.20436142	0.23419357	
Metabolic_Index	0.24103270	0.16657149	1.44702258	0.15421224	

-----

191. Results for hsa-miR-324-5p

Covariate	Beta	SE	t	p	Signif
(Intercept)	5.231338488	1.97033757	2.655046809	0.01061742	*
GroupCI	0.942751629	0.37045910	2.544819720	0.01407681	*
SexMale	-0.450365842	0.35841022	-1.256565289	0.21476811	
Visit_GroupV2	0.381938184	0.29787268	1.282219575	0.20569710	

Covariate	Beta	SE	t	p	Signif
AgeAtVisit	0.017336244	0.02694562	0.643378982	0.52292731	
APOE4_INDEX	-0.075748886	0.24735941	-0.306230057	0.76070496	
Metabolic_Index	0.001053031	0.14623969	0.007200723	0.99428345	

-----

192. Results for hsa-miR-6891-5p

Covariate	Beta	SE	t	p	Signif
(Intercept)	3.29044410	2.2557223	1.4587098	0.15065919	
GroupCI	0.90227451	0.4296415	2.1000638	0.04059213	*
SexMale	-0.63026924	0.4221721	-1.4929202	0.14150101	
Visit_GroupV2	-0.08101359	0.2839843	-0.2852749	0.77656674	
AgeAtVisit	0.02957784	0.0310456	0.9527224	0.34513843	
APOE4_INDEX	0.20692955	0.2938805	0.7041281	0.48449440	
Metabolic_Index	0.32321775	0.1689058	1.9135980	0.06118357	.

-----

193. Results for hsa-miR-486-3p

Covariate	Beta	SE	t	p	Signif
(Intercept)	1.58209804	1.68220936	0.9404882	0.3519296320	
GroupCI	0.52838825	0.32297674	1.6359947	0.1087348266	
SexMale	0.16954160	0.31361299	0.5406077	0.5914126342	
Visit_GroupV2	-0.23899024	0.26077928	-0.9164465	0.3642608541	
AgeAtVisit	0.04590278	0.02310746	1.9864916	0.0530161272	.
APOE4_INDEX	0.81261650	0.22089560	3.6787355	0.0006175997	***
Metabolic_Index	0.39001176	0.12838601	3.0378057	0.0039369711	**

-----

194. Results for hsa-miR-205-5p

Covariate	Beta	SE	t	p	Signif
(Intercept)	1.5150450	3.62328601	0.4181411	0.677504181	
GroupCI	1.8011233	0.67339194	2.6747027	0.009880042	**
SexMale	-0.8311643	0.66730132	-1.2455606	0.218309899	
Visit_GroupV2	-0.6514470	0.39905408	-1.6324780	0.108404506	
AgeAtVisit	0.1001035	0.05004224	2.0003791	0.050504671	.
APOE4_INDEX	0.0606642	0.46367506	0.1308334	0.896393881	
Metabolic_Index	0.1019843	0.26639557	0.3828301	0.703350412	

-----

195. Results for hsa-miR-11400

Covariate	Beta	SE	t	p	Signif
(Intercept)	3.97882525	1.9692409	2.0204868	0.04854236	*
GroupCI	0.83033191	0.3738709	2.2209057	0.03077639	*
SexMale	-0.49713770	0.3663369	-1.3570506	0.18067737	
Visit_GroupV2	0.41632955	0.2568368	1.6209885	0.11112142	
AgeAtVisit	0.02344677	0.0271428	0.8638301	0.39168266	
APOE4_INDEX	0.18043952	0.2532195	0.7125815	0.47931769	
Metabolic_Index	0.08295305	0.1467466	0.5652808	0.57433477	

-----

196. Results for hsa-miR-183-5p

Covariate	Beta	SE	t	p	Signif
(Intercept)	5.727108387	2.22860621	2.5698162	0.01307371	*
GroupCI	0.762553026	0.41558077	1.8349093	0.07222873	.
SexMale	-0.512498923	0.40881022	-1.2536353	0.21556869	
Visit_GroupV2	-0.087740037	0.24182478	-0.3628248	0.71820298	
AgeAtVisit	-0.009994363	0.03070443	-0.3255024	0.74610403	
APOE4_INDEX	0.357642263	0.28276441	1.2648065	0.21156679	
Metabolic_Index	0.340717549	0.16333162	2.0860477	0.04188550	*

-----

197. Results for hsa-miR-181b-5p

Covariate	Beta	SE	t	p	Signif
(Intercept)	2.03487722	1.91652249	1.0617549	0.29378780	
GroupCI	0.68000632	0.37192092	1.8283627	0.07386361	.
SexMale	0.03779897	0.35691786	0.1059038	0.91611066	
Visit_GroupV2	-0.28701792	0.31700124	-0.9054158	0.36987646	
AgeAtVisit	0.04954919	0.02623181	1.8888970	0.06510254	.
APOE4_INDEX	0.25320300	0.24905160	1.0166688	0.31453093	
Metabolic_Index	0.31974546	0.14785642	2.1625403	0.03571262	*

-----

198. Results for hsa-miR-629-5p

Covariate	Beta	SE	t	p	Signif
(Intercept)	10.295775579	1.76910850	5.8197536	4.555118e-07	***
GroupCI	-0.677459087	0.32960697	-2.0553543	4.524378e-02	*
SexMale	0.236731831	0.32883952	0.7199008	4.750370e-01	
Visit_GroupV2	-0.302020512	0.19301354	-1.5647633	1.241327e-01	
AgeAtVisit	0.007907983	0.02472282	0.3198658	7.504423e-01	
APOE4_INDEX	0.035219385	0.22977067	0.1532806	8.788122e-01	
Metabolic_Index	-0.263898624	0.13015511	-2.0275703	4.811080e-02	*

-----

199. Results for hsa-miR-151b

Covariate	Beta	SE	t	p	Signif
(Intercept)	13.72578663	1.59510905	8.6049205	1.275081e-11	***
GroupCI	-0.67521151	0.29430464	-2.2942605	2.578715e-02	*
SexMale	0.45582914	0.29822368	1.5284807	1.323700e-01	
Visit_GroupV2	-0.12999158	0.20153537	-0.6450063	5.217196e-01	
AgeAtVisit	-0.02611533	0.02253748	-1.1587507	2.517784e-01	
APOE4_INDEX	-0.03563968	0.20932756	-0.1702579	8.654588e-01	
Metabolic_Index	-0.28410934	0.11747869	-2.4183903	1.907585e-02	*

-----

200. Results for hsa-miR-15b-3p

Covariate	Beta	SE	t	p	Signif
(Intercept)	3.89303080	2.28792274	1.70155694	0.09523100	.
GroupCI	0.84691857	0.42838527	1.97700207	0.05373137	.
SexMale	-0.02680873	0.41179332	-0.06510238	0.94835965	
Visit_GroupV2	0.08181284	0.34745933	0.23546020	0.81484132	
AgeAtVisit	0.03429443	0.03130286	1.09556855	0.27866925	
APOE4_INDEX	0.14441929	0.28398372	0.50854776	0.61337100	
Metabolic_Index	0.16244658	0.16925311	0.95978493	0.34191609	

-----

201. Results for hsa-miR-23b-3p

Covariate	Beta	SE	t	p	Signif
(Intercept)	4.10135697	3.55167839	1.15476586	0.25342929	

Covariate	Beta	SE	t	p	Signif
GroupCI	1.50370290	0.65612041	2.29180935	0.02596786	*
SexMale	-0.03883131	0.65556042	-0.05923376	0.95299142	
Visit_GroupV2	-0.22613528	0.36103637	-0.62635043	0.53380634	
AgeAtVisit	0.06151864	0.04916683	1.25122244	0.21641497	
APOE4_INDEX	-0.14204452	0.45504157	-0.31215725	0.75616075	
Metabolic_Index	0.31790258	0.25803340	1.23202105	0.22344531	

-----

202. Results for hsa-miR-941

Covariate	Beta	SE	t	p	Signif
(Intercept)	2.44707773	2.05121986	1.1929866	0.238353403	
GroupCI	0.66591200	0.38610357	1.7246978	0.090592256	.
SexMale	-0.16935552	0.37862790	-0.4472875	0.656547099	
Visit_GroupV2	-0.09167928	0.23883555	-0.3838594	0.702666232	
AgeAtVisit	0.03046558	0.02829878	1.0765688	0.286700892	
APOE4_INDEX	0.29813634	0.26261029	1.1352805	0.261524920	
Metabolic_Index	0.51196952	0.15327206	3.3402664	0.001564527	**

-----

203. Results for hsa-miR-3143

Covariate	Beta	SE	t	p	Signif
(Intercept)	5.475420117	1.97000603	2.7793926	0.007581310	**
GroupCI	0.534187419	0.36677708	1.4564362	0.151333607	
SexMale	-0.318320298	0.36053031	-0.8829224	0.381375332	
Visit_GroupV2	-0.335078558	0.24220902	-1.3834273	0.172493371	
AgeAtVisit	-0.004452216	0.02697246	-0.1650652	0.869537963	
APOE4_INDEX	0.357778448	0.24927982	1.4352483	0.157253641	
Metabolic_Index	0.471524096	0.14568131	3.2366822	0.002115298	**

-----

204. Results for hsa-miR-203a-3p

Covariate	Beta	SE	t	p	Signif
(Intercept)	3.70371372	2.79611526	1.3245926	0.19117211	
GroupCI	0.97145462	0.52679251	1.8440934	0.07094321	.
SexMale	-0.25718517	0.51532124	-0.4990774	0.61985662	
Visit_GroupV2	-0.16831983	0.28648751	-0.5875294	0.55942340	

Covariate	Beta	SE	t	p	Signif
AgeAtVisit	0.02220855	0.03868765	0.5740475	0.56844208	
APOE4_INDEX	0.07239935	0.35728588	0.2026370	0.84021962	
Metabolic_Index	0.49362488	0.20851219	2.3673670	0.02172053	*

-----

205. Results for hsa-miR-375-3p

Covariate	Beta	SE	t	p	Signif
(Intercept)	7.158030997	2.68045467	2.6704540	0.009960311	**
GroupCI	0.944100278	0.50475916	1.8703975	0.066794135	.
SexMale	-1.100780059	0.49906373	-2.2056904	0.031635222	*
Visit_GroupV2	-0.659311145	0.35786963	-1.8423222	0.070862147	.
AgeAtVisit	0.004358304	0.03718337	0.1172111	0.907122990	
APOE4_INDEX	0.125470796	0.34660845	0.3619958	0.718752840	
Metabolic_Index	0.500217106	0.19786644	2.5280543	0.014393877	*

-----

206. Results for hsa-miR-323b-3p

Covariate	Beta	SE	t	p	Signif
(Intercept)	1.709594097	2.18524849	0.78233396	0.43783175	
GroupCI	0.909831689	0.41673838	2.18322029	0.03390307	*
SexMale	0.007517701	0.40775318	0.01843689	0.98536617	
Visit_GroupV2	0.441162688	0.27015713	1.63298556	0.10896598	
AgeAtVisit	0.053822188	0.03031824	1.77524130	0.08215050	.
APOE4_INDEX	0.366488341	0.28216239	1.29885609	0.20015145	
Metabolic_Index	-0.058189784	0.16303437	-0.35691729	0.72270623	

-----

207. Results for hsa-miR-409-3p

Covariate	Beta	SE	t	p	Signif
(Intercept)	1.97745751	2.03414514	0.97213196	0.336412124	
GroupCI	1.06381313	0.39311037	2.70614368	0.009715384	**
SexMale	0.03334585	0.37918092	0.08794179	0.930330496	
Visit_GroupV2	0.26559197	0.31912705	0.83224524	0.409860655	
AgeAtVisit	0.05828057	0.02800158	2.08133163	0.043381694	*
APOE4_INDEX	0.20374636	0.26343225	0.77342981	0.443491739	
Metabolic_Index	-0.17632982	0.15348283	-1.14885698	0.256954212	

-----

208. Results for hsa-miR-1180-3p

Covariate	Beta	SE	t	p	Signif
(Intercept)	1.01313985	1.8095510	0.5598847	0.578207055	
GroupCI	0.52697813	0.3468244	1.5194380	0.135326404	
SexMale	0.14124117	0.3379068	0.4179885	0.677852039	
Visit_GroupV2	-0.56529863	0.2493164	-2.2673948	0.027988461	*
AgeAtVisit	0.05736818	0.0250239	2.2925357	0.026379493	*
APOE4_INDEX	0.61973235	0.2362308	2.6234186	0.011689286	*
Metabolic_Index	0.39055815	0.1366731	2.8576084	0.006332706	**

-----

209. Results for hsa-miR-4448

Covariate	Beta	SE	t	p	Signif
(Intercept)	-2.4177476	4.7489342	-0.5091137	0.61276329	
GroupCI	1.5606285	0.8935472	1.7465540	0.08644675	.
SexMale	-0.4156217	0.8829079	-0.4707419	0.63973631	
Visit_GroupV2	-0.5933308	0.3107125	-1.9095810	0.06154860	.
AgeAtVisit	0.1103081	0.0660269	1.6706534	0.10062348	
APOE4_INDEX	0.9949531	0.6161269	1.6148509	0.11221845	
Metabolic_Index	0.8687240	0.3518359	2.4691173	0.01676576	*

-----

210. Results for hsa-miR-8485

Covariate	Beta	SE	t	p	Signif
(Intercept)	9.08091386	3.85519819	2.3554986	0.022225859	*
GroupCI	1.08052506	0.72673463	1.4868220	0.142979330	
SexMale	-0.84706035	0.71639869	-1.1823868	0.242322867	
Visit_GroupV2	0.03838271	0.36206173	0.1060115	0.915972941	
AgeAtVisit	-0.03085254	0.05354435	-0.5762053	0.566912224	
APOE4_INDEX	0.85936359	0.49669827	1.7301522	0.089415271	.
Metabolic_Index	0.80954493	0.28576014	2.8329526	0.006506077	**

-----

211. Results for hsa-miR-598-3p

Covariate	Beta	SE	t	p	Signif
(Intercept)	4.39950869	2.03184254	2.1652803	0.03529465	*
GroupCI	0.81431356	0.38644119	2.1072121	0.04027379	*
SexMale	-0.34204505	0.37507926	-0.9119274	0.36629990	
Visit_GroupV2	0.52920740	0.28367535	1.8655389	0.06813668	.
AgeAtVisit	0.02221336	0.02783621	0.7980026	0.42874381	
APOE4_INDEX	0.29364464	0.26105361	1.1248442	0.26616949	
Metabolic_Index	-0.17479759	0.15110563	-1.1567907	0.25300167	

-----

212. Results for hsa-miR-3138

Covariate	Beta	SE	t	p	Signif
(Intercept)	1.35269562	1.93519642	0.6989966	0.48780272	
GroupCI	0.67329247	0.37341218	1.8030812	0.07742120	.
SexMale	-0.27158367	0.36223898	-0.7497362	0.45693735	
Visit_GroupV2	0.25229267	0.31416620	0.8030548	0.42575543	
AgeAtVisit	0.05395409	0.02654959	2.0322005	0.04747872	*
APOE4_INDEX	0.46989100	0.25385920	1.8509907	0.07009992	.
Metabolic_Index	0.18257896	0.14733727	1.2391907	0.22107750	

-----

213. Results for hsa-miR-139-3p

Covariate	Beta	SE	t	p	Signif
(Intercept)	5.611781172	2.02932639	2.76534184	0.007974224	**
GroupCI	0.673157506	0.38283104	1.75836708	0.084879170	.
SexMale	-0.042276561	0.36590944	-0.11553832	0.908486844	
Visit_GroupV2	0.192000541	0.29943693	0.64120528	0.524353225	
AgeAtVisit	0.002649664	0.02769384	0.09567698	0.924164204	
APOE4_INDEX	-0.211985332	0.25301357	-0.83784176	0.406155183	
Metabolic_Index	0.401009676	0.15250235	2.62953114	0.011367372	*

-----

214. Results for hsa-miR-140-3p

Covariate	Beta	SE	t	p	Signif
(Intercept)	6.646481949	2.41777442	2.7490083	0.008448918	**

Covariate	Beta	SE	t	p	Signif
GroupCI	0.894363726	0.45042940	1.9855803	0.052917575	.
SexMale	0.129040791	0.43539269	0.2963779	0.768244156	
Visit_GroupV2	-0.342432425	0.33005662	-1.0374960	0.304800851	
AgeAtVisit	0.004316850	0.03311291	0.1303676	0.896830730	
APOE4_INDEX	-0.133923707	0.30012029	-0.4462334	0.657473035	
Metabolic_Index	-0.004133547	0.17811790	-0.0232068	0.981583331	

-----

215. Results for hsa-miR-589-5p

Covariate	Beta	SE	t	p	Signif
(Intercept)	4.805402799	1.91083449	2.5148190	0.01508010	*
GroupCI	0.615824876	0.36039924	1.7087297	0.09353701	.
SexMale	-0.396758994	0.35328641	-1.1230520	0.26663662	
Visit_GroupV2	0.248603682	0.24507779	1.0143868	0.31515212	
AgeAtVisit	0.009084709	0.02625361	0.3460365	0.73072848	
APOE4_INDEX	0.161026493	0.24417596	0.6594691	0.51254024	
Metabolic_Index	0.288941746	0.14233092	2.0300701	0.04754283	*

-----

216. Results for hsa-miR-151a-5p

Covariate	Beta	SE	t	p	Signif
(Intercept)	14.23222487	1.53116583	9.2950251	1.712671e-12	***
GroupCI	-0.53632970	0.28312190	-1.8943420	6.394968e-02	.
SexMale	0.31889474	0.28753529	1.1090630	2.726899e-01	
Visit_GroupV2	0.34132661	0.17500629	1.9503677	5.672876e-02	.
AgeAtVisit	-0.00575173	0.02158179	-0.2665085	7.909397e-01	
APOE4_INDEX	-0.27988173	0.20388173	-1.3727651	1.759293e-01	
Metabolic_Index	-0.27334457	0.11289507	-2.4212269	1.912561e-02	*

-----

217. Results for hsa-miR-28-3p

Covariate	Beta	SE	t	p	Signif
(Intercept)	11.26411075	2.14690136	5.2466829	1.811114e-06	***
GroupCI	-0.71649535	0.39216190	-1.8270397	7.228101e-02	.
SexMale	0.45280268	0.39445774	1.1479118	2.552084e-01	
Visit_GroupV2	0.92188178	0.36557094	2.5217589	1.413448e-02	*

Covariate	Beta	SE	t	p	Signif
AgeAtVisit	-0.02086906	0.03037021	-0.6871557	4.944268e-01	
APOE4_INDEX	-0.14003012	0.27281830	-0.5132725	6.094993e-01	
Metabolic_Index	-0.27938297	0.15614038	-1.7893063	7.821994e-02	.

-----

218. Results for hsa-miR-652-3p

Covariate	Beta	SE	t	p	Signif
(Intercept)	3.62543563	2.09810022	1.7279611	0.09018145	.
GroupCI	0.75884075	0.39595427	1.9164859	0.06104105	.
SexMale	-0.12395964	0.38548702	-0.3215663	0.74912476	
Visit_GroupV2	0.47604343	0.28875000	1.6486352	0.10550671	
AgeAtVisit	0.03395421	0.02880999	1.1785571	0.24416556	
APOE4_INDEX	0.25493264	0.26761043	0.9526259	0.34537264	
Metabolic_Index	-0.03352815	0.15530425	-0.2158869	0.82995620	

-----

219. Results for hsa-miR-4516

Covariate	Beta	SE	t	p	Signif
(Intercept)	4.745109031	2.14893859	2.2081176	0.0319359303	*
GroupCI	0.466503538	0.40435707	1.1536921	0.2542106236	
SexMale	-0.036494284	0.39183696	-0.0931364	0.9261741438	
Visit_GroupV2	0.144518232	0.34587146	0.4178380	0.6778869939	
AgeAtVisit	0.004096518	0.02924391	0.1400811	0.8891689219	
APOE4_INDEX	0.187646311	0.27284087	0.6877500	0.4948459856	
Metabolic_Index	0.587815984	0.16313493	3.6032502	0.0007322184	***

-----

220. Results for hsa-miR-4433b-3p

Covariate	Beta	SE	t	p	Signif
(Intercept)	4.00963374	2.14342576	1.8706660	0.06740494	.
GroupCI	0.93502248	0.40045946	2.3348743	0.02371572	*
SexMale	-0.18381662	0.38660736	-0.4754607	0.63658373	
Visit_GroupV2	0.81881887	0.33938633	2.4126454	0.01964781	*
AgeAtVisit	0.03911370	0.02935342	1.3325092	0.18889258	
APOE4_INDEX	-0.04367828	0.26622850	-0.1640631	0.87036028	
Metabolic_Index	-0.27462994	0.15687986	-1.7505748	0.08631488	.

DE miRNAs in CI vs NC: Common to both MAs and NHWs
Adjusted for the covariables in NHW

221. Results for hsa-miR-6515-5p

Covariate	Beta	SE	t	p	Signif
(Intercept)	5.826539203	1.72324947	3.3811351	0.0010302190	**
GroupCI	1.186702557	0.32941577	3.6024461	0.0004925836	***
SexMale	-0.293711951	0.31218810	-0.9408173	0.3490603336	
Visit_GroupV2	0.383281421	0.30573281	1.2536483	0.2128859762	
AgeAtVisit	-0.003444033	0.02330983	-0.1477502	0.8828366979	
APOE4_INDEX	0.078097015	0.21834669	0.3576744	0.7213390559	
Metabolic_Index	0.142442265	0.13055405	1.0910597	0.2778623490	

-----

222. Results for hsa-miR-199a-5p

Covariate	Beta	SE	t	p	Signif
(Intercept)	4.22795726	2.10775717	2.0059034	0.0501526817	.
GroupCI	1.59370805	0.40404970	3.9443367	0.0002437516	***
SexMale	-0.43397014	0.39481094	-1.0991847	0.2768184727	
Visit_GroupV2	0.37339191	0.26613268	1.4030292	0.1666308062	
AgeAtVisit	0.01285281	0.02906341	0.4422333	0.6601789522	
APOE4_INDEX	0.09166351	0.27419253	0.3343035	0.7395132521	
Metabolic_Index	0.08386769	0.15773126	0.5317126	0.5972198151	

-----

223. Results for hsa-let-7i-3p

Covariate	Beta	SE	t	p	Signif
(Intercept)	3.29773511	2.04737082	1.61071706	0.1133314371	
GroupCI	1.42280428	0.39056879	3.64290313	0.0006247606	***
SexMale	-0.13289261	0.37897285	-0.35066526	0.7272644871	
Visit_GroupV2	-0.30110391	0.22702214	-1.32631960	0.1905640530	
AgeAtVisit	0.02121508	0.02831251	0.74931818	0.4570618851	
APOE4_INDEX	0.02542386	0.26405889	0.09628103	0.9236696761	
Metabolic_Index	0.27989267	0.15329942	1.82579081	0.0736604120	.

-----

224. Results for hsa-miR-4665-5p

Covariate	Beta	SE	t	p	Signif
(Intercept)	4.80258160	1.77568709	2.7046328	0.0093465275	**
GroupCI	1.17759785	0.33641472	3.5004349	0.0009924974	***
SexMale	-0.13427881	0.32746878	-0.4100507	0.6835358477	
Visit_GroupV2	0.13734064	0.24979739	0.5498082	0.5849201622	
AgeAtVisit	-0.00505579	0.02440532	-0.2071593	0.8367342152	
APOE4_INDEX	0.13919054	0.22835695	0.6095305	0.5449571271	
Metabolic_Index	0.47595091	0.13436428	3.5422427	0.0008751121	***

-----

225. Results for hsa-let-7g-3p

Covariate	Beta	SE	t	p	Signif
(Intercept)	2.50799841	1.88308697	1.3318548	0.188793030	
GroupCI	1.29157516	0.35801801	3.6075704	0.000700156	***
SexMale	-0.27964784	0.34986074	-0.7993119	0.427793724	
Visit_GroupV2	-0.03046094	0.21791623	-0.1397828	0.889378771	
AgeAtVisit	0.02751088	0.02606024	1.0556650	0.296065797	
APOE4_INDEX	0.21711325	0.24326268	0.8925054	0.376288112	
Metabolic_Index	0.36556960	0.14111736	2.5905360	0.012444339	*

-----

226. Results for hsa-miR-106a-5p

Covariate	Beta	SE	t	p	Signif
(Intercept)	2.88289848	1.80838030	1.5941882	0.1170001512	
GroupCI	1.19606404	0.34225403	3.4946675	0.0009837649	***
SexMale	-0.24643588	0.33614841	-0.7331163	0.4668043532	
Visit_GroupV2	0.08984589	0.20691134	0.4342241	0.6659356383	
AgeAtVisit	0.02033322	0.02501901	0.8127111	0.4201168250	
APOE4_INDEX	0.26144865	0.23350387	1.1196759	0.2680355675	
Metabolic_Index	0.40162309	0.13498200	2.9753825	0.0044438675	**

-----

227. Results for hsa-miR-1294

Covariate	Beta	SE	t	p	Signif
(Intercept)	3.96114155	1.96133473	2.0196153	0.049087306	*

Covariate	Beta	SE	t	p	Signif
GroupCI	1.09982974	0.37659584	2.9204511	0.005333128	**
SexMale	0.14893655	0.36387640	0.4093053	0.684156263	
Visit_GroupV2	-0.24193762	0.28054814	-0.8623747	0.392816372	
AgeAtVisit	0.01621866	0.02691645	0.6025559	0.549671437	
APOE4_INDEX	0.45015900	0.25560334	1.7611624	0.084650036	.
Metabolic_Index	0.35726254	0.14923487	2.3939616	0.020668125	*

-----

228. Results for hsa-miR-98-3p

Covariate	Beta	SE	t	p	Signif
(Intercept)	3.89915655	1.85962370	2.0967449	0.040945232	*
GroupCI	1.18781070	0.35278444	3.3669589	0.001443891	**
SexMale	-0.29107431	0.34564112	-0.8421287	0.403609207	
Visit_GroupV2	0.15258292	0.22083983	0.6909212	0.492717270	
AgeAtVisit	0.01078003	0.02567929	0.4197946	0.676381457	
APOE4_INDEX	0.13406703	0.24015616	0.5582494	0.579092805	
Metabolic_Index	0.33664929	0.13920947	2.4182931	0.019166259	*

-----

229. Results for hsa-miR-7-1-3p

Covariate	Beta	SE	t	p	Signif
(Intercept)	5.316153444	1.74783965	3.0415567	0.003748234	**
GroupCI	1.048571337	0.33313957	3.1475437	0.002779615	**
SexMale	-0.146042124	0.32451945	-0.4500258	0.654644321	
Visit_GroupV2	0.336053523	0.24956877	1.3465367	0.184224887	
AgeAtVisit	-0.009346228	0.02398889	-0.3896066	0.698488346	
APOE4_INDEX	0.355889886	0.22592527	1.5752549	0.121528299	
Metabolic_Index	0.236288888	0.13105202	1.8030160	0.077436429	.

-----

230. Results for hsa-miR-21-3p

Covariate	Beta	SE	t	p	Signif
(Intercept)	3.47169867	1.77499304	1.9558942	0.056019568	.
GroupCI	1.11317891	0.33744784	3.2988178	0.001783905	**
SexMale	-0.28065010	0.33028856	-0.8497119	0.399495060	
Visit_GroupV2	0.09922955	0.22934442	0.4326661	0.667099233	

Covariate	Beta	SE	t	p	Signif
AgeAtVisit	0.01580623	0.02448422	0.6455679	0.521481276	
APOE4_INDEX	0.23599915	0.22941439	1.0287025	0.308520686	
Metabolic_Index	0.34335881	0.13322912	2.5772055	0.012925224	*

-----

231. Results for hsa-miR-320e

Covariate	Beta	SE	t	p	Signif
(Intercept)	4.724480786	1.70864876	2.7650392	0.007945950	**
GroupCI	1.029051408	0.32440474	3.1721220	0.002584159	**
SexMale	0.093556525	0.31398213	0.2979677	0.766959925	
Visit_GroupV2	0.108298733	0.21955196	0.4932715	0.623975865	
AgeAtVisit	0.006762638	0.02348747	0.2879253	0.774591572	
APOE4_INDEX	0.033572188	0.21816336	0.1538856	0.878318119	
Metabolic_Index	0.276002652	0.12876897	2.1433940	0.036960136	*

-----

232. Results for hsa-miR-6877-5p

Covariate	Beta	SE	t	p	Signif
(Intercept)	3.21994062	1.88942557	1.7041902	0.094315227	.
GroupCI	1.07864639	0.35768002	3.0156741	0.003960315	**
SexMale	-0.12168242	0.35112878	-0.3465464	0.730330629	
Visit_GroupV2	0.10737588	0.20478725	0.5243290	0.602277949	
AgeAtVisit	0.01591497	0.02613595	0.6089301	0.545222127	
APOE4_INDEX	0.42264980	0.24471980	1.7270764	0.090090768	.
Metabolic_Index	0.33334434	0.14080756	2.3673753	0.021670885	*

-----

233. Results for hsa-miR-7113-5p

Covariate	Beta	SE	t	p	Signif
(Intercept)	2.81460527	2.21180389	1.2725383	0.209092833	
GroupCI	1.25425629	0.42411084	2.9573785	0.004734841	**
SexMale	-0.09786801	0.41184318	-0.2376342	0.813140073	
Visit_GroupV2	-0.34403434	0.30140897	-1.1414204	0.259155394	
AgeAtVisit	0.02778830	0.03049015	0.9113861	0.366483775	
APOE4_INDEX	0.27662625	0.28776558	0.9612903	0.341051835	
Metabolic_Index	0.41679955	0.16753314	2.4878633	0.016244670	*

-----

234. Results for hsa-let-7f-1-3p

Covariate	Beta	SE	t	p	Signif
(Intercept)	3.60957875	1.98668866	1.8168819	0.075275518	.
GroupCI	1.16678443	0.37730470	3.0924195	0.003254593	**
SexMale	-0.25893438	0.36804989	-0.7035306	0.485012481	
Visit_GroupV2	0.13245397	0.24144609	0.5485861	0.585748833	
AgeAtVisit	0.01544213	0.02742343	0.5631000	0.575901808	
APOE4_INDEX	0.18056542	0.25566575	0.7062558	0.483331267	
Metabolic_Index	0.37153142	0.14931065	2.4883115	0.016237434	*

-----

235. Results for hsa-miR-381-3p

Covariate	Beta	SE	t	p	Signif
(Intercept)	3.42743337	2.00631216	1.7083251	0.093675623	.
GroupCI	1.18701495	0.38016202	3.1223923	0.002958088	**
SexMale	-0.49441996	0.37302969	-1.3254172	0.190958314	
Visit_GroupV2	-0.10843653	0.23068467	-0.4700639	0.640319594	
AgeAtVisit	0.02019747	0.02771636	0.7287199	0.469516608	
APOE4_INDEX	0.31997107	0.25856598	1.2374833	0.221592737	
Metabolic_Index	0.27496228	0.14926327	1.8421296	0.071292094	.

-----

236. Results for hsa-miR-19a-3p

Covariate	Beta	SE	t	p	Signif
(Intercept)	4.293419473	2.15799925	1.9895371	0.051806724	.
GroupCI	1.252100825	0.40775377	3.0707277	0.003363518	**
SexMale	-0.541896807	0.40066876	-1.3524808	0.181957049	
Visit_GroupV2	0.023305867	0.20195976	0.1153986	0.908564791	
AgeAtVisit	0.008333633	0.02988086	0.2788954	0.781409152	
APOE4_INDEX	0.136582344	0.27826374	0.4908377	0.625564990	
Metabolic_Index	0.391006286	0.16085999	2.4307243	0.018481058	*

-----

237. Results for hsa-let-7a-3p

Covariate	Beta	SE	t	p	Signif
(Intercept)	5.640007794	2.32783145	2.4228592	0.01877700	*
GroupCI	1.343710452	0.43868190	3.0630633	0.00341114	**
SexMale	-0.238620309	0.42765386	-0.5579753	0.57916367	
Visit_GroupV2	0.246746457	0.22786320	1.0828710	0.28366983	
AgeAtVisit	-0.007834785	0.03219448	-0.2433580	0.80864874	
APOE4_INDEX	-0.169220213	0.29665433	-0.5704289	0.57074913	
Metabolic_Index	0.260893289	0.17266037	1.5110201	0.13660432	

-----

238. Results for hsa-miR-1303

Covariate	Beta	SE	t	p	Signif
(Intercept)	2.92017436	1.97649367	1.4774519	0.145631905	
GroupCI	1.07428195	0.37276369	2.8819383	0.005747160	**
SexMale	-0.18307900	0.36606821	-0.5001226	0.619114785	
Visit_GroupV2	-0.18883227	0.20206636	-0.9345062	0.354395162	
AgeAtVisit	0.02076487	0.02736863	0.7587105	0.451477252	
APOE4_INDEX	0.27153360	0.25479203	1.0657068	0.291517000	
Metabolic_Index	0.47568688	0.14737402	3.2277527	0.002170193	**

-----

239. Results for hsa-miR-3613-5p

Covariate	Beta	SE	t	p	Signif
(Intercept)	3.19593055	2.02114562	1.5812471	0.119830020	
GroupCI	1.10762283	0.38079274	2.9087289	0.005309944	**
SexMale	-0.40788295	0.37487124	-1.0880615	0.281539446	
Visit_GroupV2	-0.19704842	0.21562367	-0.9138534	0.364973228	
AgeAtVisit	0.01888515	0.02796072	0.6754171	0.502375939	
APOE4_INDEX	0.21419547	0.26000807	0.8238032	0.413779435	
Metabolic_Index	0.44863021	0.15025735	2.9857456	0.004290386	**

-----

240. Results for hsa-miR-192-5p

Covariate	Beta	SE	t	p	Signif
(Intercept)	7.23145890	2.06344467	3.5045567	0.00102596	**

Covariate	Beta	SE	t	p	Signif
GroupCI	0.95778266	0.38578592	2.4826791	0.01672108	*
SexMale	0.20085755	0.36740825	0.5466876	0.58721618	
Visit_GroupV2	-0.27506127	0.31447875	-0.8746577	0.38627079	
AgeAtVisit	-0.02075158	0.02806375	-0.7394444	0.46336654	
APOE4_INDEX	0.09869086	0.25562624	0.3860748	0.70120810	
Metabolic_Index	0.23780717	0.15349846	1.5492479	0.12812509	

-----

241. Results for hsa-miR-3611

Covariate	Beta	SE	t	p	Signif
(Intercept)	2.20322301	1.91703480	1.1492869	0.255753263	
GroupCI	1.03756424	0.36336282	2.8554496	0.006182972	**
SexMale	-0.25738135	0.35689388	-0.7211705	0.474069565	
Visit_GroupV2	0.14150737	0.23470494	0.6029160	0.549213533	
AgeAtVisit	0.03153836	0.02652573	1.1889724	0.239910081	
APOE4_INDEX	0.26975032	0.24786733	1.0882851	0.281541771	
Metabolic_Index	0.38625029	0.14361617	2.6894625	0.009624590	**

-----

242. Results for hsa-miR-7847-3p

Covariate	Beta	SE	t	p	Signif
(Intercept)	3.665623182	2.08769582	1.7558224	0.085230086	.
GroupCI	1.066170762	0.39318100	2.7116538	0.009147598	**
SexMale	-0.330243640	0.38721091	-0.8528779	0.397785608	
Visit_GroupV2	0.025656120	0.24685519	0.1039319	0.917638313	
AgeAtVisit	0.009775104	0.02882827	0.3390805	0.735966103	
APOE4_INDEX	0.279976626	0.26853814	1.0425954	0.302140109	
Metabolic_Index	0.521270802	0.15560629	3.3499340	0.001542264	**

-----

243. Results for hsa-miR-885-5p

Covariate	Beta	SE	t	p	Signif
(Intercept)	1.98270330	1.73488386	1.1428450	0.2585322148	
GroupCI	0.93996772	0.32700823	2.8744466	0.0059242566	**
SexMale	-0.31128670	0.32217456	-0.9662051	0.3385802485	
Visit_GroupV2	0.04716245	0.22441588	0.2101565	0.8343974575	

Covariate	Beta	SE	t	p	Signif
AgeAtVisit	0.03238316	0.02398126	1.3503529	0.1829683561	
APOE4_INDEX	0.37024133	0.22412800	1.6519191	0.1048026346	
Metabolic_Index	0.53748445	0.13023948	4.1268933	0.0001386971	***

-----

244. Results for hsa-miR-3928-3p

Covariate	Beta	SE	t	p	Signif
(Intercept)	4.499069411	1.74046542	2.584980636	0.01287327	*
GroupCI	0.771872742	0.33061364	2.334666941	0.02384528	*
SexMale	-0.002725565	0.32153853	-0.008476637	0.99327227	
Visit_GroupV2	0.325913828	0.27108366	1.202262905	0.23523188	
AgeAtVisit	0.002597596	0.02377374	0.109263251	0.91345483	
APOE4_INDEX	0.522934908	0.22580492	2.315870249	0.02493791	*
Metabolic_Index	0.273239916	0.13047031	2.094268975	0.04160682	*

-----

245. Results for hsa-miR-195-5p

Covariate	Beta	SE	t	p	Signif
(Intercept)	7.66577014	2.0558500	3.7287594	0.0004614327	***
GroupCI	0.98898175	0.3826557	2.5845209	0.0124761096	*
SexMale	-0.47454230	0.3732006	-1.2715476	0.2089621541	
Visit_GroupV2	-0.09305790	0.2576943	-0.3611174	0.7194151593	
AgeAtVisit	-0.03026618	0.0280742	-1.0780782	0.2857726026	
APOE4_INDEX	0.03884860	0.2582525	0.1504287	0.8809846980	
Metabolic_Index	0.27824506	0.1512360	1.8398075	0.0712740754	.

-----

246. Results for hsa-miR-2278

Covariate	Beta	SE	t	p	Signif
(Intercept)	4.5943405211	1.77794070	2.58407973	0.012712593	*
GroupCI	0.7386783443	0.33542266	2.20223149	0.032259434	*
SexMale	-0.0704385573	0.32899461	-0.21410246	0.831332520	
Visit_GroupV2	-0.1074840314	0.23014641	-0.46702459	0.642498577	
AgeAtVisit	0.0007663955	0.02440732	0.03140024	0.975074588	

Covariate	Beta	SE	t	p	Signif
APOE4_INDEX	0.5574396881	0.22880954	2.43626068	0.018421533	*
Metabolic_Index	0.3608289326	0.13251896	2.72284762	0.008872052	**

-----

247. Results for hsa-miR-3149

Covariate	Beta	SE	t	p	Signif
(Intercept)	2.89936139	2.37870718	1.2188812	0.22838606	
GroupCI	1.11240437	0.44779116	2.4842035	0.01624027	*
SexMale	-0.39631415	0.44161591	-0.8974182	0.37362916	
Visit_GroupV2	-0.07983042	0.25648240	-0.3112511	0.75685328	
AgeAtVisit	0.02386614	0.03294282	0.7244716	0.47202120	
APOE4_INDEX	0.28033748	0.30650198	0.9146351	0.36460204	
Metabolic_Index	0.41508043	0.17670189	2.3490435	0.02265349	*

-----

248. Results for hsa-miR-376b-3p

Covariate	Beta	SE	t	p	Signif
(Intercept)	2.25507813	2.02418534	1.1140670	0.270412638	
GroupCI	1.00655689	0.38397440	2.6214166	0.011478602	*
SexMale	-0.25491460	0.37565404	-0.6785887	0.500430531	
Visit_GroupV2	-0.34239156	0.21484828	-1.5936435	0.117123405	
AgeAtVisit	0.03577701	0.02802167	1.2767621	0.207401941	
APOE4_INDEX	0.13736055	0.26081928	0.5266503	0.600693076	
Metabolic_Index	0.45242524	0.15171719	2.9820301	0.004362846	**

-----

249. Results for hsa-miR-32-5p

Covariate	Beta	SE	t	p	Signif
(Intercept)	4.680799971	2.18518105	2.1420651	0.03682237	*
GroupCI	1.017933064	0.40924167	2.4873641	0.01606552	*
SexMale	-0.466748676	0.39898585	-1.1698377	0.24732307	
Visit_GroupV2	-0.009189751	0.25122406	-0.0365799	0.97095800	
AgeAtVisit	0.011181056	0.03008705	0.3716235	0.71166128	
APOE4_INDEX	0.136824303	0.27708020	0.4938076	0.62349132	
Metabolic_Index	0.277901588	0.16142462	1.7215564	0.09100816	.

-----

250. Results for hsa-let-7c-5p

Covariate	Beta	SE	t	p	Signif
(Intercept)	7.65094834	1.62480220	4.7088491	1.610819e-05	***
GroupCI	0.87962650	0.29692820	2.9624216	4.429378e-03	**
SexMale	-0.37265203	0.29635808	-1.2574384	2.136677e-01	
Visit_GroupV2	-0.47805231	0.18685591	-2.5584008	1.316708e-02	*
AgeAtVisit	0.04884857	0.02258726	2.1626608	3.473073e-02	*
APOE4_INDEX	0.12517977	0.20655578	0.6060337	5.468725e-01	
Metabolic_Index	0.09273223	0.11784959	0.7868693	4.345829e-01	

-----

251. Results for hsa-miR-627-5p

Covariate	Beta	SE	t	p	Signif
(Intercept)	3.82472097	2.20929866	1.7311924	0.08929491	.
GroupCI	0.92893440	0.41799389	2.2223636	0.03059336	*
SexMale	0.25158294	0.40658369	0.6187728	0.53874347	
Visit_GroupV2	0.08339107	0.25053875	0.3328470	0.74057617	
AgeAtVisit	0.01947763	0.03059243	0.6366814	0.52710066	
APOE4_INDEX	0.25737730	0.28249709	0.9110795	0.36642062	
Metabolic_Index	0.17228896	0.16398336	1.0506490	0.29823525	

-----

252. Results for hsa-miR-19b-3p

Covariate	Beta	SE	t	p	Signif
(Intercept)	7.07375910	2.62336550	2.6964444	0.009362945	**
GroupCI	1.37845788	0.48663845	2.8326119	0.006505922	**
SexMale	-0.83368749	0.47595954	-1.7515932	0.085603785	.
Visit_GroupV2	0.19598695	0.32871770	0.5962166	0.553557652	
AgeAtVisit	-0.00468614	0.03613261	-0.1296928	0.897298316	
APOE4_INDEX	-0.39372318	0.32680367	-1.2047697	0.233621471	
Metabolic_Index	0.22980218	0.19218071	1.1957609	0.237089631	

-----

253. Results for hsa-miR-651-5p

Covariate	Beta	SE	t	p	Signif
(Intercept)	4.3843780	1.9610527	2.2357268	0.029758807	*
GroupCI	0.7599980	0.3704630	2.0514815	0.045354775	*
SexMale	-0.3588850	0.3623946	-0.9903155	0.326679434	
Visit_GroupV2	-0.2916677	0.2355239	-1.2383782	0.221226696	
AgeAtVisit	0.0106719	0.0269561	0.3958992	0.693825219	
APOE4_INDEX	0.2652281	0.2512598	1.0555928	0.296112417	
Metabolic_Index	0.4662719	0.1468651	3.1748313	0.002538697	**

-----

254. Results for hsa-miR-760

Covariate	Beta	SE	t	p	Signif
(Intercept)	5.266195252	2.17989702	2.41580002	0.01897041	*
GroupCI	0.853272421	0.40868144	2.08786683	0.04134724	*
SexMale	-0.159452499	0.39901359	-0.39961672	0.69095124	
Visit_GroupV2	0.686686238	0.27603018	2.48772157	0.01584400	*
AgeAtVisit	-0.002224672	0.02986752	-0.07448465	0.94088884	
APOE4_INDEX	0.315730617	0.27717068	1.13911981	0.25947801	
Metabolic_Index	0.045737584	0.15978427	0.28624585	0.77574077	

-----

255. Results for hsa-miR-431-3p

Covariate	Beta	SE	t	p	Signif
(Intercept)	3.84940454	1.83139596	2.10189638	0.04062641	*
GroupCI	0.71576262	0.35344893	2.02508074	0.04822029	*
SexMale	-0.03222768	0.33982348	-0.09483654	0.92482410	
Visit_GroupV2	0.03560717	0.28201648	0.12625920	0.90003362	
AgeAtVisit	0.02322710	0.02502192	0.92826991	0.35773177	
APOE4_INDEX	0.30164629	0.23718301	1.27178708	0.20933848	
Metabolic_Index	0.12664209	0.13914941	0.91011591	0.36713244	

-----

256. Results for hsa-miR-5010-5p

Covariate	Beta	SE	t	p	Signif
(Intercept)	6.563135441	2.05373632	3.1957050	0.002471765	**
GroupCI	0.830783405	0.39130140	2.1231291	0.038945444	*
SexMale	0.357055528	0.37489456	0.9524159	0.345675287	

Covariate	Beta	SE	t	p	Signif
Visit_GroupV2	0.126755889	0.29900334	0.4239280	0.673519154	
AgeAtVisit	-0.008640017	0.02801134	-0.3084472	0.759081716	
APOE4_INDEX	0.390543845	0.26181458	1.4916810	0.142350365	
Metabolic_Index	-0.195178344	0.15192758	-1.2846801	0.205092122	

-----

257. Results for hsa-let-7a-5p

Covariate	Beta	SE	t	p	Signif
(Intercept)	15.237112666	1.72983947	8.8083969	6.952528e-12	***
GroupCI	0.496224847	0.32313848	1.5356414	1.306969e-01	
SexMale	-0.241714415	0.32076414	-0.7535581	4.545176e-01	
Visit_GroupV2	-0.359750988	0.14836601	-2.4247535	1.883363e-02	*
AgeAtVisit	0.009555258	0.02413177	0.3959618	6.937553e-01	
APOE4_INDEX	0.246049074	0.22343706	1.1012008	2.758871e-01	
Metabolic_Index	-0.016682257	0.12816417	-0.1301632	8.969402e-01	

258. Results for hsa-miR-181a-5p

Covariate	Beta	SE	t	p	Signif
(Intercept)	5.459969067	2.21798079	2.4616846	0.01712645	*
GroupCI	0.623184677	0.41168396	1.5137454	0.13604898	
SexMale	0.093131908	0.39966769	0.2330234	0.81664352	
Visit_GroupV2	-0.138246229	0.30215496	-0.4575342	0.64916278	
AgeAtVisit	0.008809665	0.03047725	0.2890570	0.77366871	
APOE4_INDEX	0.224997389	0.27725587	0.8115153	0.42071015	
Metabolic_Index	0.331068801	0.16398348	2.0189156	0.04858250	*

-----

259. Results for hsa-miR-376c-3p

Covariate	Beta	SE	t	p	Signif
(Intercept)	2.47323942	2.52288676	0.9803212	0.33136100	
GroupCI	1.11177207	0.47151419	2.3578762	0.02208514	*
SexMale	-0.58319787	0.46408635	-1.2566581	0.21436261	
Visit_GroupV2	-0.06814769	0.27017068	-0.2522394	0.80182718	
AgeAtVisit	0.05899400	0.03487892	1.6913942	0.09660728	.
APOE4_INDEX	-0.20784409	0.31887157	-0.6518113	0.51732669	

Covariate	Beta	SE	t	p	Signif
Metabolic_Index	0.02922055	0.18502332	0.1579290	0.87511015	
